# The Basal Transcription Complex Component TAF3 Transduces Changes in Nuclear Phosphoinositides into Transcriptional Output

**DOI:** 10.1016/j.molcel.2015.03.009

**Published:** 2015-05-07

**Authors:** Yvette Stijf-Bultsma, Lilly Sommer, Maria Tauber, Mai Baalbaki, Panagiota Giardoglou, David R. Jones, Kathy A. Gelato, Jason van Pelt, Zahid Shah, Homa Rahnamoun, Clara Toma, Karen E. Anderson, Philip Hawkins, Shannon M. Lauberth, Anna-Pavlina G. Haramis, Daniel Hart, Wolfgang Fischle, Nullin Divecha

**Affiliations:** 1 The Inositide Laboratory, Centre for Biological Sciences, Highfield Campus, University of Southampton, Southampton SO171BJ, UK; 2 The Inositide Laboratory, the CRUK Manchester Institute, the University of Manchester, Wilmslow Road, Manchester M204BX, UK; 3 Laboratory of Chromatin Biochemistry, Max Planck Institute for Biophysical Chemistry, Am Fassberg 11, 37077 Göttingen, Germany; 4 University of California, San Francisco, Mail Code 3120, Smith Cardiovascular Research Building, 555 Mission Bay Boulevard, South San Francisco, CA 94158-9001, USA; 5 Institute of Biology (IBL), Leiden University, Sylvius Laboratory, Sylviusweg 72, 2333 BE Leiden, the Netherlands; 6 Division of Biological Sciences, Department of Molecular Biology, University of California, San Diego, La Jolla, CA 92093, USA; 7 Signaling Laboratory, The Babraham Institute, Cambridge, Cambridgeshire CB22 3AT, UK

## Abstract

Phosphoinositides (PI) are important signaling molecules in the nucleus that influence gene expression. However, if and how nuclear PI directly affects the transcriptional machinery is not known. We report that the lipid kinase PIP4K2B regulates nuclear PI5P and the expression of myogenic genes during myoblast differentiation. A targeted screen for PI interactors identified the PHD finger of TAF3, a TATA box binding protein-associated factor with important roles in transcription regulation, pluripotency, and differentiation. We show that the PI interaction site is distinct from the known H3K4me3 binding region of TAF3 and that PI binding modulates association of TAF3 with H3K4me3 in vitro and with chromatin in vivo. Analysis of TAF3 mutants indicates that TAF3 transduces PIP4K2B-mediated alterations in PI into changes in specific gene transcription. Our study reveals TAF3 as a direct target of nuclear PI and further illustrates the importance of basal transcription components as signal transducers.

## Highlights

• PIP4K2B regulates nuclear PI5P and myogenic gene expression during differentiation
• A screen identifies 17 of 32 PHD fingers interacting with phosphoinositides
• The basal transcription component TAF3 interacts strongly with phosphoinositides
• TAF3 transduces changes in nuclear phosphoinositides into transcriptional output

## Graphical Abstract

**Figure:**
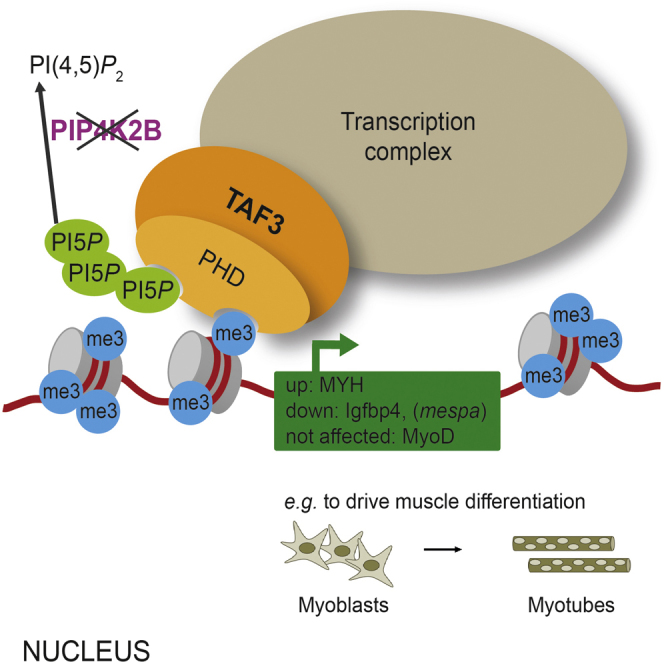

## Teaser

Bultsma et al. show that the basal transcriptional complex protein TAF3 directly binds phosphoinositides and transduces changes in nuclear phosphoinositides into differential transcriptional output that affects myoblast differentiation. The lipid kinase PIP4K2B, phosphoinositides, and TAF3 form a conserved nuclear signaling pathway that selectively regulates transcription.

## Introduction

Pre-initiation complex (PIC) formation of eukaryotic type II RNA polymerase requires the general transcription factor TFIID. TFIID contains the TATA box binding protein (TBP) and TBP-associated factors (TAFs) and nucleates the PIC on core promoters by binding to the TATA box (Burley and Roeder, 1996; Thomas and Chiang, 2006). Recent studies have demonstrated that tissue-specific and selective gene transcription can be imposed by core promoter components (Hochheimer and Tjian, 2003). For instance, TAF3 regulates specific gene transcription as part of the large canonical TFIID PIC, and of a developmentally regulated smaller transcription complex with TBP and the TBP-like protein TRF3 (Deato and Tjian, 2007, 2008; Hart et al., 2009; Maston et al., 2012). These non-canonical complexes are involved in the regulation of transcriptional programs during differentiation (Hart et al., 2009; Deato and Tjian, 2007).

How TAF3 regulates gene expression is incompletely understood. Its plant homeodomain (PHD) finger mediates the interaction with histone H3 trimethylated at lysine 4 (H3K4me3) (Vermeulen et al., 2007), which is required to regulate selective gene expression in response to stress signaling (Vermeulen et al., 2007; Lauberth et al., 2013). However, chromatin association studies have shown that while TAF3 and TFIID complexes are constitutively associated with H3K4me3, knockdown of TAF3 does not strongly affect constitutive gene expression (Lauberth et al., 2013; Liu et al., 2011), suggesting that other induced regulators may influence TAF3’s role as a transducer of cellular inputs to control differential transcriptional outputs.

Phosphoinositides (PI) are a family of seven signaling lipids regulating a wide variety of cellular processes including cell survival, proliferation, adhesion, and ion channel function (van den Bout and Divecha, 2009). PI are interconverted by the action of kinases and phosphatases, and degraded by phospholipases (Maffucci, 2012). The presence of different PI-modulating enzymes in subcellular compartments generates compartment-specific PI profiles that underpin organelle identity and function. PI and PI-modulating enzymes are present in the nuclear membrane, as well as within the nucleus (Shah et al., 2013; Divecha et al., 1991; Lindsay et al., 2006; Watt et al., 2004). In the latter, they are associated with interchromatin domains and affect nuclear functions. For example, the level of phosphatidylinositol 5-phosphate (PI5P) in chromatin is increased in response to stress signaling (Jones et al., 2006) and affects p53 acetylation and gene transcription (Gozani et al., 2003).

PIP4K2A and 2B are isoforms within the PIP4K family of lipid kinases that phosphorylate and remove nuclear PI5P (Rameh et al., 1997; Bultsma et al., 2010; Wang et al., 2010; Clarke et al., 2007; Jones et al., 2006). PIP4K2A and 2B are deregulated in human cancers (Jude et al., 2014; Keune et al., 2013; Emerling et al., 2013), leading to expression changes of genes involved in cell-cycle progression, epithelial-mesenchymal transition (EMT), reactive oxygen accumulation and metabolism, ultimately affecting tumor growth. However, how PIP4K and PI5P directly influence specific gene transcription is yet unknown.

Nuclear PI regulate processes through their interaction with proteins containing PI-interacting domains (Lewis et al., 2011; Bidlingmaier and Liu, 2007; Bidlingmaier et al., 2011; Jungmichel et al., 2014) and their identification has linked nuclear PI to protein folding, DNA and chromatin regulation (Lewis et al., 2011), mRNA splicing and polyadenylation (Lewis et al., 2011; Mellman et al., 2008), and allosteric regulation of histone binding modules (Gelato et al., 2014). The PHD finger of Inhibitor of growth protein 2 (ING2) was one of the first convincingly shown to interact with nuclear PI, including PI5P (Gozani et al., 2003). PHD-fingers are present predominantly in nuclear proteins (Bienz, 2006) and are often mutated or deleted in human diseases (Musselman and Kutateladze, 2009). They are zinc-finger motifs facilitating protein/protein and protein/ligand interactions, for example by recognizing specific histone modifications (Pena et al., 2006a; Wysocka et al., 2006). The interaction of PHD fingers with both PI and histone tails suggests possible direct cross talk between a lipid signaling pathway and chromatin.

PIP4K2B is highly expressed in muscle tissue and, in this study, we show that it regulates the expression of muscle-specific genes as well as the extent of myoblast differentiation. We also show that the PHD finger is a common motif for PI binding, and that the basal transcription component TAF3 is a strong interactor of PI, particularly PI5P. Detailed studies revealed a pathway linking the PIP4K2B signal transduction pathway directly to the regulation of TAF3 and gene expression.

## Results

### The Nuclear PI Kinase PIP4K2B Regulates Myoblast Differentiation

**Figure 1:**
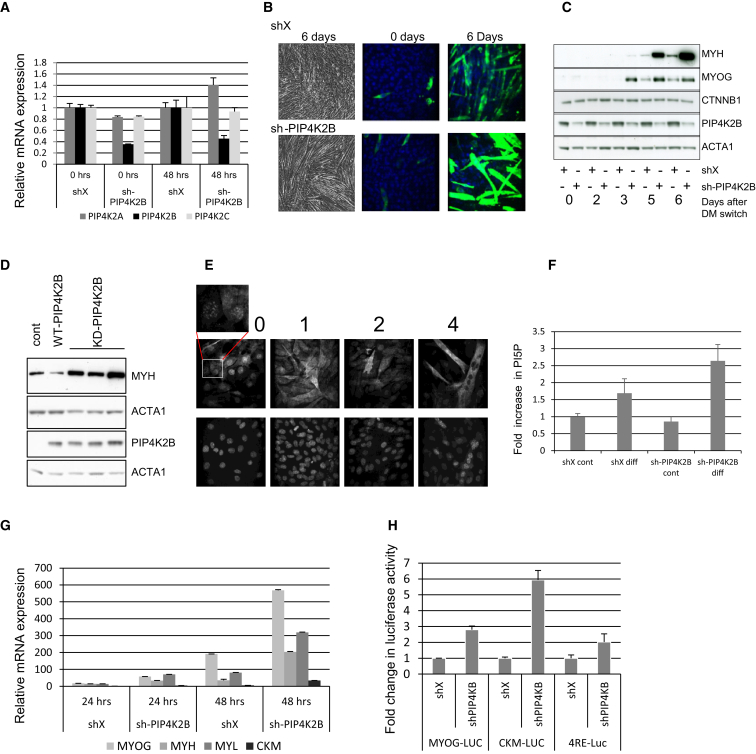
PIP4K2B Regulates Myogenic Differentiation in C2C12 Myoblasts (A) Control (shX) and PIP4K2B knockdown (sh-PIP4K2B) C2C12 cells were differentiated for 0 hr or 48 hr and expression of PIP4K isoforms was determined by QRT-PCR. (B) shX or sh-PIP4K2B C2C12 cells were differentiated for the indicated times, fixed, stained with an anti-MYH antibody (right four images), and myotube formation was depicted with by brightfield microscopy (left images). (C) shX or sh-PIP4K2B C2C12 cells were differentiated for the times shown and the levels of proteins indicated were determined by immunoblotting. (D) Control (Cont), wild-type PIP4K2B (WT-PIP4K2B), or kinase inactive PIP4K2B (KD-PIP4K2B) overexpressing C2C12 cells were differentiated for 4 days and lysed. Levels of proteins indicated were determined by immunoblotting. (E) C2C12 cells overexpressing PIP4K2B were fixed at the indicated times (top). Top: localization of PIP4K2B as assessed by antibody staining. Bottom: nuclear staining (DAPI). The inset shows a higher magnification of the cells in the square. (F) shX or sh-PIP4K2B C2C12 cells were differentiated for 2 days before isolation of nuclei by hypotonic lysis. PI5P levels were measured using a specific mass assay. (G) shX or sh-PIP4K2B C2C12 cells were differentiated for the times indicated (hours) and expression levels of indicated genes were determined with QRT-PCR. (H) shX or sh-PIP4K2B C2C12 cells were transfected with luciferase constructs driven by the MYOG, CKM, or a synthetic MRF promoter (4RE) and differentiated for 48 hr. Luciferase activity was measured and normalized to firefly luciferase driven by a CMV promoter. The values in (A), (F), (G), and (H) show fold changes compared to shX sample and represent the mean of triplicates +SD. See also Figure S1.

Because PIP4K2B is highly expressed in muscle tissue, we analyzed the role of PIP4K2B in myoblast differentiation using the C2C12 cell model. C2C12 myoblast cells can exit the cell cycle, synthesize muscle specific proteins, such as myogenin (MYOG) and myosin heavy chain (MYH), and finally fuse to form large multinucleate myotubes, resembling the differentiation of primary muscle myoblasts (Asp et al., 2011). We generated C2C12 cell lines stably depleted of PIP4K2B expression, without strongly affecting expression of the 2A or 2C PIP4K-isoforms (Figure 1A).

Although PIP4K2B knockdown did not significantly alter cell-cycle progression during differentiation (Figure S1A), it strongly increased myotube formation (Figure 1B) and significantly increased the myoblast fusion index (shX = 4.2 ± 1.2 [SD] and sh-PIP4K2B = 10.59 ± 3.0 [SD], p < 0.000001 Student’s t test). PIP4K2B depletion increased the expression of late (MYH) (Figure 1B) and (early (MYOG) (Figure 1C) myogenic markers. Overexpression of wild-type kinase active PIP4K2B suppressed MYH expression whereas kinase-inactive mutants of PIP4K2B increased the expression of MYH in a similar manner to the knockdown of PIP4K2B (Figure 1D). These data show that PIP4K2B kinase activity is important in controlling the expression of muscle-specific proteins and the extent of C2C12 myoblasts differentiation.

PIP4K2B localizes in the nucleus in many cell types (Bultsma et al., 2010; Jones et al., 2006; Richardson et al., 2007; Boronenkov et al., 1998; Ciruela et al., 2000) and we thus assessed its localization in C2C12 cells undergoing differentiation. In 88% of undifferentiated C2C12 myoblasts, PIP4K2B was nuclear and showed defined punctate nuclear staining (Figure 1E and 0 days). After 1 day of differentiation, PIP4K2B staining became diffuse throughout the cell and after 4 days when myotubes were formed, PIP4K2B was excluded from the nuclei (Figure 1E, day 4). Loss of endogenous nuclear PIP4K2B was also observed by immunoblotting (Figure S1B). Correlating with decreased nuclear PIP4K2B during differentiation, we observed a 1.7-fold increase in nuclear PI5P levels. Depletion of PIP4K2B further increased nuclear PI5P levels (Figure 1F). Knockdown of PIP4K2B did not alter the nuclear levels of total PIP (predominantly PI4P) or PIP_2_ (predominantly PI(4,5)P_2_) as assessed by mass spectrometry. A small decrease in both phosphatidylserine and phosphatidylinositol was observed (Figure S1C).

Because PIP4K2B is nuclear in undifferentiated cells, we postulated that it might affect myoblast differentiation by regulating gene expression. Knockdown of PIP4K2B increased the expression of early and late myogenic marker genes including MYOG, MYH, myosin light chain (MYL), and muscle creatine kinase (CKM) (Figures 1G and S1D). Increased expression of myogenic markers was observed with three different sh-RNA constructs targeting PIP4K2B (Figure S1E) and all three increased myotube formation (Figure S1F). The increased expression of MYH and MYL could be partially rescued by overexpression of a PIP4K2B-PIP4K2A fusion protein (Figure S1G). Increased myogenic gene expression was not solely a consequence of increased numbers of cells undergoing differentiation as they were increased in isolated myotubes (Yoshida et al., 1998) depleted of PIP4K2B (Figure S1H). Increased myogenic transcription was implicated using luciferase expression driven by endogenous promoters of MYOG, CKM or from a synthetic MRF promoter (4RE) which revealed increased transcriptional activity upon PIP4K2B depletion (Figure 1H).

These data are consistent with a role for PIP4K2B in regulating nuclear PI5P levels that affect the expression of muscle-specific genes during myogenic differentiation.

### PHD Finger Containing Proteins as Potential Downstream Targets of PIP4K2B

**Figure 2:**
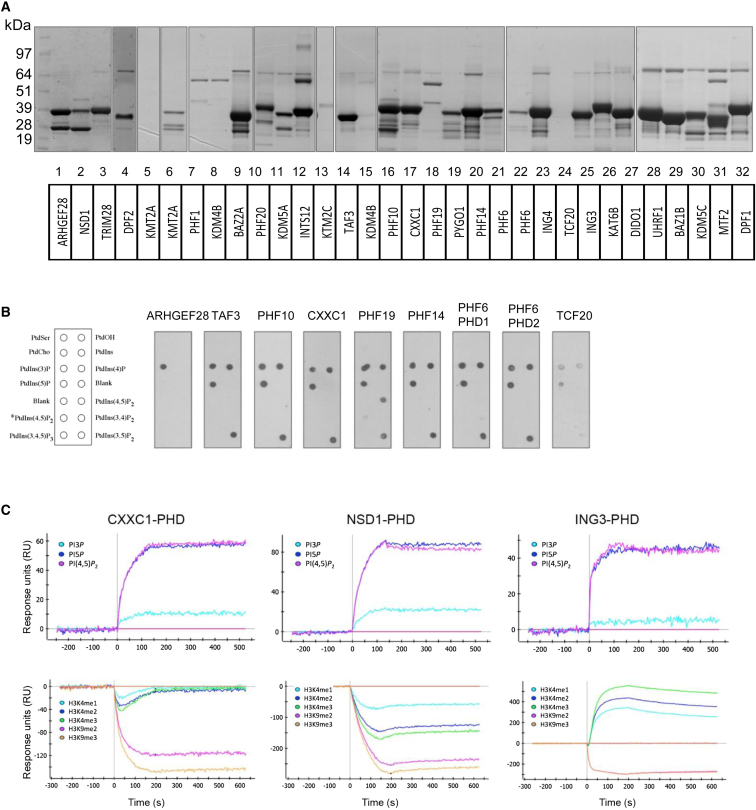
A Small-Scale Screen Identifies PHD Fingers as PI Interactors (A) 32 PHD finger sequences were PCR-amplified, cloned into pGEX-4T1, and expressed and purified as GST-fusion proteins. Proteins were analyzed by SDS-PAGE and Coomassie blue staining. Numbers correspond to numbers in Table S1. (B) Lipid dot blots (schematic left) were probed with GST-PHD fingers as indicated and interactions were visualized using an anti-GST antibody. (C) GST-PHD fingers were analyzed for interaction with PI (top) and histone H3 peptides (bottom) by SPR. Positive controls are shown in Figure S2. The PI-PHD finger interaction was dependent on the presence of Zn in the analyte buffer, which could not be replaced by magnesium. ING3 strongly interacts with H3K4me3 peptide, whereas both CXXC1 and NSD1 interact best with unmodified H3 (as shown by negative SPR responses after referencing to H3-unmodified peptide). See also Figure S2 and Table S1.

We reasoned that PHD finger containing proteins may be plausible downstream targets to couple changes in nuclear PI to gene expression (Gozani et al., 2003; Ndamukong et al., 2010). We cloned 32 different human PHD fingers and expressed them as GST fusion proteins in bacteria. Purified proteins (Figure 2A) were tested for interaction with PI using lipid dot blots (Figure 2B) and surface plasmon resonance (SPR) (Figures 2C and S2), together identified 17 PHD fingers as interactors of PI (Table S1). The specificity of PI interaction differed between the two lipid interaction assays, presumably reflecting differing presentation of the lipids.

PHD fingers also interact with methylated (Peña et al., 2006b; Shi et al., 2006) and non-methylated histone H3 tails (Rajakumara et al., 2011). Only three of the 32 PHD fingers preferentially interacted with a methylation modified H3 peptide (TAF3, ING3, and DIDO1), whereas many of the others preferred unmodified H3 peptide, which in some cases was reduced by methylation of the peptide at either K4 or K9 (Figure 2C and Table S1). For example, CXXC1 bound preferentially to the unmodified H3 peptide, while methylation of K9 but not K4 decreased its interaction (Figure 2C).

### The PHD Finger of the Basal Transcription Complex Component TAF3 Interacts with PI

**Figure 3:**
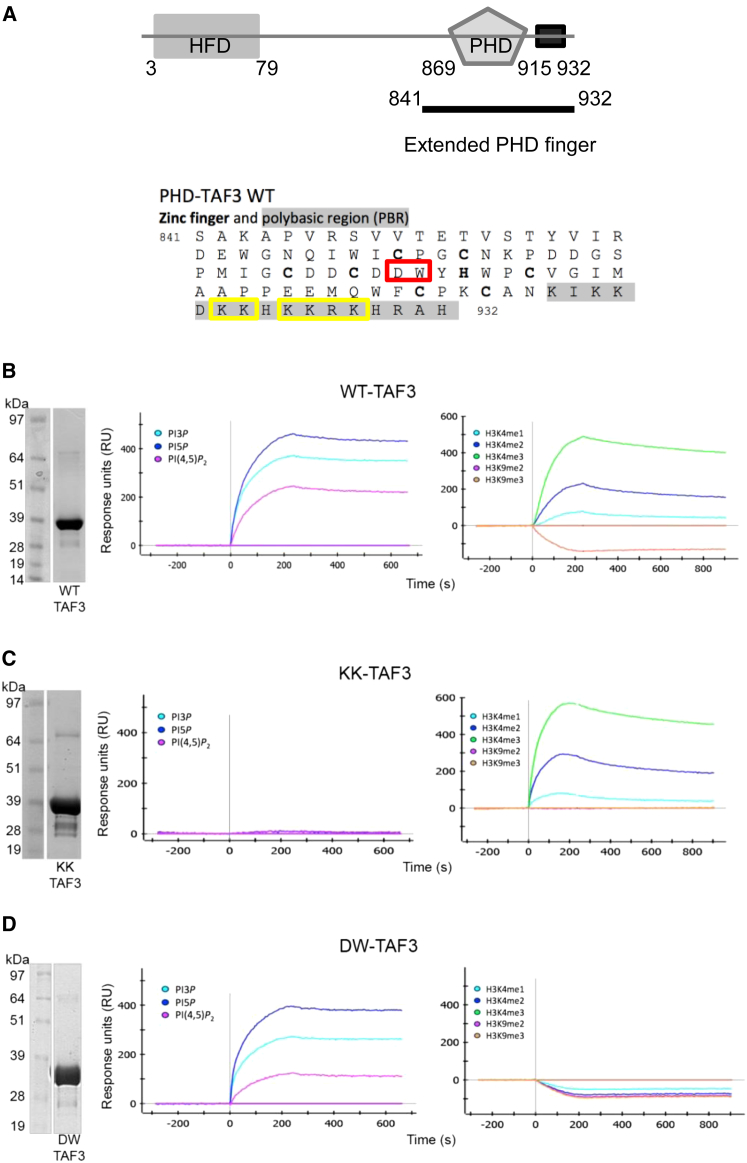
The Extended PHD Finger of TAF3 Mediates Its Interaction with PI (A) Cartoon depicting the structure of TAF3 including the extended PHD. Amino acids in bold are required for the interaction with zinc, those boxed in red are required for the interaction with H3K4me3 and mutated in the DW-TAF3 mutant, and those boxed in yellow are mutated in the KK-TAF3 mutant to attenuate interaction with PI. (B–D) Interaction of WT (B), KK mutant (C), and DW mutant (D) TAF3 PHD finger with PI (left) and modified histone peptides (right) assessed with SPR. See also Figure S3.

Among the 17 PI interacting PHD fingers, we identified TAF3 (Figure 3A), a component of the basal transcription complex that interacts with H3K4me3 (Vermeulen et al., 2007) (Figure 3B) and is a regulator of C2C12 myoblast differentiation and muscle-specific gene transcription (Deato et al., 2008; Deato and Tjian, 2007).

We first determined how PI interacted with the PHD finger of TAF3. Mutation of conserved cysteine residues for zinc coordination ablated interaction with PI, demonstrating the requirement for an intact PHD finger (Figure S3A). Alignment of PI interacting PHD finger sequences suggested that a polybasic region (PBR) at the C terminus may be important for the interaction with PI (data not shown), which was confirmed by deletion of the PBR (Figures S3B–S3E). Combinatorial mutagenesis of lysine 922, 923, 925, 926 and 928, and arginine 927 completely ablated PI interaction of PHD-TAF3, while maintaining the specificity and interaction of the PHD finger with H3K4me3 peptide (Figures 3C and S3B–S3E). In contrast, mutagenesis of D890 and W891 residues disrupted the H3K4me3 interaction (Figure 3D, right) but did not significantly alter its interaction with PI (Figure 3D, left).

These data show that the PHD finger of TAF3 interacts independently with PI and histone methylation marks enabling the generation of PHD-TAF3 mutants that only maintain H3K4me3 (KK-TAF3) or PI interaction (DW-TAF3), which we used to further study the relationship between PI5P and TAF3 during myoblast differentiation.

### PI Interaction Regulates TAF3-Dependent Gene Expression during Myoblast Differentiation

**Figure 4:**
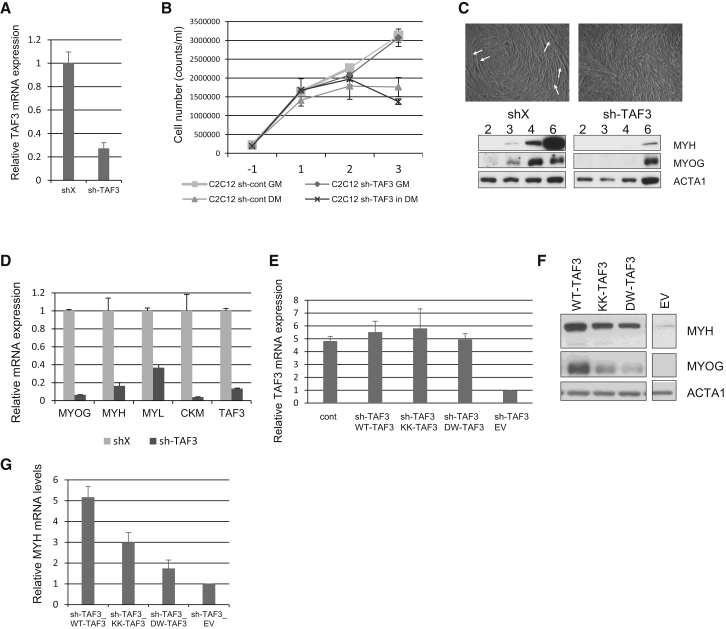
TAF3-Mediated Gene Expression during Differentiation Requires the PI and H3K4me3 Interaction Sites (A) A combination of two sh-constructs targeting the 3′UTR of mouse TAF3 were used to generate C2C12 cells with stable TAF3 knockdown, and assessed for TAF3 expression compared to shX with QRT-PCR. (B) Proliferation of shX or sh-TAF3 C2C12 cells in growth (GM) or differentiation medium (DM). The graph shows cell numbers and days of differentiation, and data represent the mean of triplicates + SD. (C) shX or sh-TAF3 C2C12 cells were differentiated for 6 days and myotubes were depicted with brightfield microscopy. Cell lysates from cells differentiated for the days indicated were analyzed for protein expression by immunoblotting. (D) shX or sh-TAF3 C2C12 cells were differentiated for 4 days and gene expression changes determined by QRT-PCR as indicated. (E) TAF3-depleted C2C12 cells (sh-TAF3) were reconstituted with wild-type TAF3 (WT-TAF3), or mutants that only maintain H3K4me3 (KK-TAF3) or PI interaction (DW-TAF3) respectively, or with empty vector (EV). Control cells were not depleted of TAF3 (cont). TAF3 expression was determined by QRT-PCR. (F) TAF3-depleted C2C12 cells were reconstituted with the indicated constructs and were differentiated for 4 days and levels of proteins indicated were determined by immunoblotting. (G) TAF3-depleted C2C12 cells (sh-TAF3) were reconstituted as indicated and were differentiated for 4 days and MYH expression was determined by QRT-PCR. The values in (A), (D), (E), and (G) show fold changes compared to control sample (shX for A and D; shTAF3-EV for E and G) and represent the mean of triplicates +SD. See also Figure S4.

Stable knockdown of TAF3 in C2C12 cells (Figures 4A and S4) did not alter their cell growth or the cessation of proliferation upon differentiation (Figure 4B), but severely attenuated C2C12 myoblast differentiation (Deato and Tjian, 2007) and expression of MYOG and MYH (Figure 4C). As expected, mRNA levels of specific myogenic markers (MYOG, CMK, MYL, and MYH) were reduced in TAF3 knockdown cells (Figures 4D and S4A).

TAF3-depleted cells were rescued using either murine wild-type (WT) TAF3 or mutants that only maintain H3K4me3 (KK-TAF3) or PI interaction (DW-TAF3), which were all similarly expressed (Figure 4E). These cell lines were differentiated and the expression of myogenic markers was determined. As expected, the expression of both MYH and MYOG was rescued by WT TAF3 (Figure 4F), whereas their expression was compromised by rescue with either KK-TAF3 or DW-TAF3 (Figures 4F and 4G). Late-stage myotube formation was also compromised in both the KK- and the DW-TAF3 mutant (Figure S4B).

These data show that PHD finger interactions with both PI and H3K4me3 are required for TAF3 to drive proper muscle-specific gene transcription during differentiation of C2C12 cells.

### PIP4K2B Regulates Specific Gene Expression through the PI Interacting Site of TAF3

**Figure 5:**
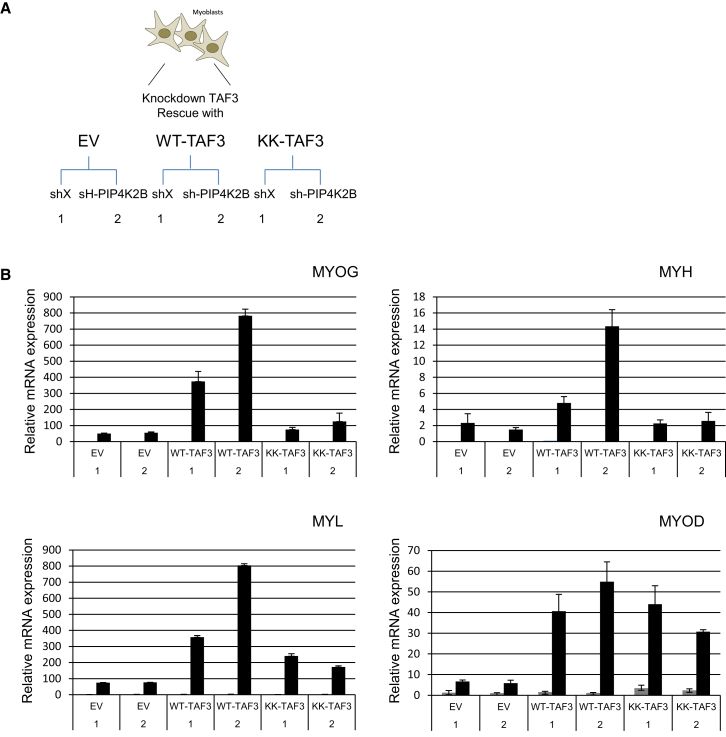
PIP4K2B Increases Gene Expression during Differentiation through the PI Interaction Site of TAF3 (A) Scheme depicting the cell types generated and used to study the relationship between PIP4K2B and TAF3. (B) C2C12 cells depicted in Figure 5A were differentiated and gene expression determined by QRT-PCR as indicated. Expression of early myogenic markers (MYOG, MYOD) was assessed after 2 days of differentiation, whereas late myogenic marker expression (MYH, CKM) was assessed at 4 days. The full time course is shown in Figure S5B. 1 indicates control knockdown (shX) and 2 indicates knockdown of PIP4K2B (sh-PIP4K2B) in the respective TAF3 rescue cell lines. The data represent fold changes compared to the 0 hr shX sample (not shown) and represent the mean of triplicates + SD. See also Figure S5.

We next explored the specific relationship between PIP4K2B expression and the TAF3-PI interaction site in the regulation of muscle differentiation. Specifically, we analyzed if myogenic genes that are regulated by PIP4K2B knockdown require the PI interaction site of TAF3. TAF3-depleted cells were rescued with either WT or KK-TAF3 and were additionally depleted of PIP4K2B (Figures 5A and S5A). QRT-PCR and immunoblotting showed that WT and KK-TAF3 (Figures S5A and S5B) were expressed to similar levels. After differentiation the expression levels of MYOG, MYH and MYL were rescued by the expression of WT-TAF3 and were further increased when PIP4K2B was depleted (Figure 5B, bars 3 and 4). KK-TAF3-expressing cells showed attenuated expression of myogenic markers that were not further induced by PIP4K2B depletion (Figures 5B, S5C, and S5D). In contrast, expression of the myogenic factor MYOD, which is dependent on TAF3 expression, was similarly expressed in both WT and KK-TAF3 rescued cells (Figures 5B, S5C, and S5D). These data directly link TAF3-PI interaction to the regulation of the expression of a subset of myogenic markers.

### PIP4K2B Positively and Negatively Regulates TAF3-Mediated Gene Expression

To gain further insight into the relationship between PIP4K2B and transcriptional regulation by TAF3, we carried out microarray gene expression analysis of the cell lines outlined in Figure 5A, which were either differentiated for 2 days or treated with etoposide for seven hours. Etoposide treatment increased nuclear PI5P in other cell types (Jones et al., 2006), allowing the study of TAF3-PI interaction in a setting unrelated to differentiation (accession number GSE66353). Principal component (PC) analysis (Figure S6A) of the top 500 most variable genes showed that the biological triplicate arrays for each genotype were highly coherent and that the variation within the data sets could cluster C2C12 cells based on their specific genotype. Gene set enrichment analysis (GSEA) (Subramanian et al., 2005) indicated that gene expression programmes upregulated either during myoblast differentiation (GSE19968) (van Oevelen et al., 2010) or by MYOD overexpression were highly enriched in cells with PIP4K2B depletion and in cells expressing WT-TAF3 compared to KK-TAF3 (Figure S6B). However, depletion of PIP4K2B or expression of KK-TAF3 did not affect all aspects of differentiation because genes that are normally downregulated during differentiation (van Oevelen et al., 2010) were not enriched (data not shown).

**Figure 6:**
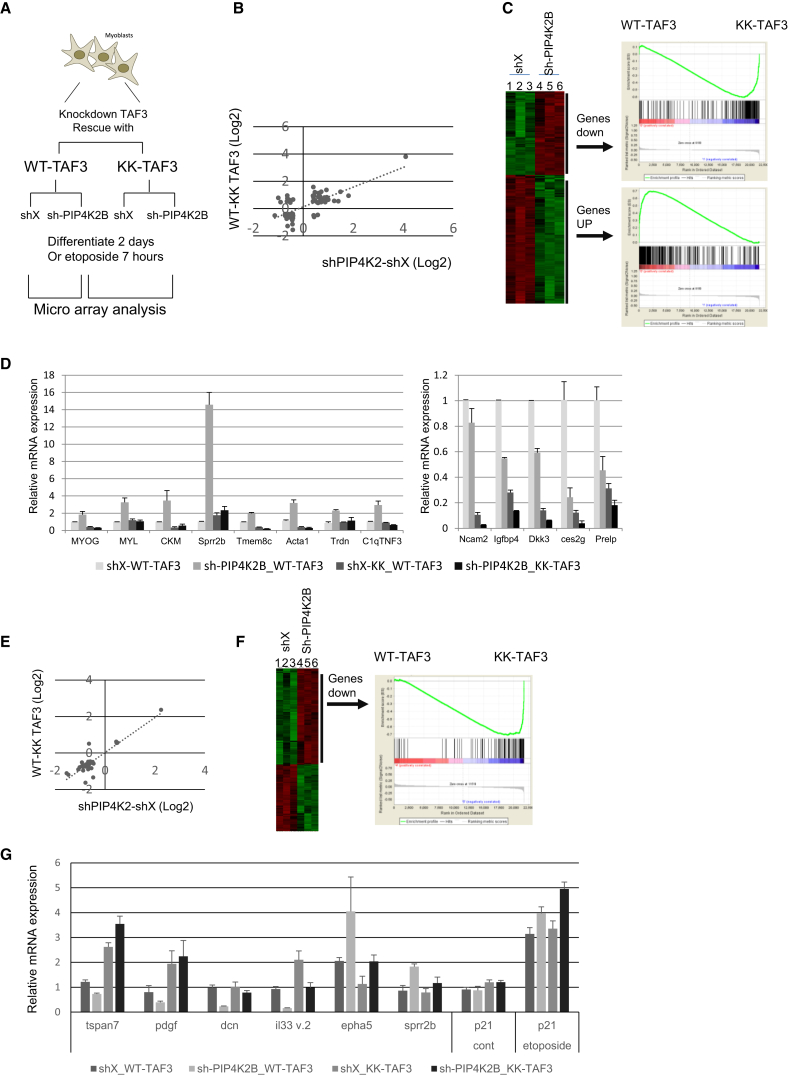
PIP4K2B and TAF3 Coordinate Both Positive and Negative Regulation of Gene Expression during Differentiation and Etoposide Treatment (A) Scheme depicting cells used for microarray gene expression analysis. Comparisons were aimed at identifying genes regulated by PIP4K2B knockdown and PI-TAF3 interaction during differentiation or etoposide treatment of C2C12 cells. (B) Correlation plot of changes in the expression of the 63 overlapping genes (Figure S6C) regulated by PIP4K2B depletion and expression of KK-TAF3 after differentiation for 2 days. (C) Left: heatmap showing expression of genes in shX (1, 2, and 3) and sh-PIP4K2B (4, 5, and 6) knockdown C2C12 cells differentiated for 2 days. Defined gene sets that were up- or downregulated were used for GSEA to probe a ranked list of gene expression changes between WT-TAF3- and KK-TAF3-expressing cells. GSEA demonstrated that genes upregulated upon PIP4K2B knockdown (increased nuclear PI5P) are highly enriched in WT-TAF3 (interacts with PI5P) expressing cells (normalized enrichment score 3.22 and FWER p = 0.0 with 156 of 335 enriched) whereas downregulated genes are highly enriched in KK-TAF3 (does not interact with PI5P) (normalized enrichment score −3.21 FWER p = 0.0 with 112 of 215 genes enriched). (D) Gene expression was assessed by QRT-PCR as indicated in C2C12 cells depicted in Figure 6A after differentiation for 2 days. (E) Correlation plot of changes in the expression of the 30 overlapping genes (Figure S6D) regulated by PIP4K2B depletion and expression of KK-TAF3 after etoposide treatment. (F) Left: heatmap showing expression of genes in triplicate shX (1, 2, and 3) and sh-PIP4K2B (4, 5, and 6) C2C12 cells after etoposide treatment. A gene set that was decreased upon PIP4K2B depletion was used for GSEA to probe a ranked list of gene expression changes between WT-TAF3- and KK-TAF3-expressing cells. Genes downregulated by PIP4K2B depletion (increased nuclear PI5P) are more highly expressed in cells expressing KK-TAF3 compared to WT-TAF3. (G) Gene expression was assessed by QRT-PCR as indicated in C2C12 cells depicted in Figure 6A after etoposide treatment for 7 hr. The values in (D) and (G) represent fold changes compared to the shX-WT-TAF3 sample and represent the mean of triplicates +SD. See also Figure S6.

Comparative expression analysis showed that 550 genes changed upon depletion of PIP4K2B (p < 0.05, >1.4-fold difference), of which 331 were upregulated. 202 genes were differentially expressed between WT and KK-TAF3 rescued C2C12 cells (p < 0.05, 1.4-fold cut off) (Figure S6C). Sixty-three genes were deregulated in both conditions (Figure S6B) and analysis of changes in the overlapping genes showed that those that are increased by PIP4K2B depletion are decreased in KK-expressing cells. Conversely, genes downregulated when PIP4K2B is depleted are more highly expressed in KK-TAF3 rescue cells (Figure 6B). GSEA also demonstrated that genes upregulated upon PIP4K2B knockdown (increase in nuclear PI5P) were highly enriched in WT-TAF3 expressing cells, whereas those downregulated in PIP4K2B knockdown cells were highly enriched in KK-TAF3 cells. These data suggest that PIP4K2B regulates gene transcriptional output by changing the levels of nuclear PI, which modulates TAF3 function by directly interacting with the PBR domain of TAF3.

To verify the microarray analysis we analyzed gene expression by QRT-PCR. Another six genes (CKM, Sprr2b, Tmem8C, Acta1,Trdn, and C1qTNF3) showed similar patterns of expression as MYOG, MYH, and MYL (Figures 5B and 6D). Their upregulation when PIP4K2B was depleted was suppressed in KK-TAF3 rescue cells (Figure 6D). We also identified another group of genes (Ncam2, Igfbp4, Dkk3, Ces2g, and Prelp) whose expression was partially decreased in PIP4K2B knockdown cells and strongly decreased in KK-TAF3 cells (Figure 6D).

In etoposide-treated cells, 236 genes were differentially expressed in PIP4K2B-depleted cells, whereas 127 genes were differentially expressed between WT-TAF3 and KK-TAF3 rescue cells, with a highly significant overlap of 30 genes (Figure S6D). These 30 genes again highlighted a co-regulated response. However, most of the genes in the overlap were downregulated in PIP4K2B depleted cells and correspondingly upregulated in KK-TAF3 rescue cells (Figure 6E). GSEA of the genes downregulated by PIP4K2B knockdown (increase in PI5P) showed that they were more highly expressed in KK-TAF3 compared to WT-TAF3 rescue cells (normalized enrichment score −1.408, FWER p = 0.05 with 72 of 151 genes enriched) (Figure 6F). However, there was no enrichment of genes upregulated by PIP4K2B knockdown (data not shown). QRT-PCR showed a decrease in expression of Tspan7, Pdgf, IL33 v.2, and Decorin (Dcn) in response to PIP4K2B depletion in WT-TAF3 rescue cells, which was suppressed in KK-TAF3 rescue cells. EpH5 and Sprr2b expression increased upon PIP4K2B knockdown, which was suppressed in KK-TAF3 rescue cells. Expression of p21 was not effected by any of the conditions but as expected was upregulated in response to etoposide (Figure 6G).

The simplest interpretation of these data is that PIP4K2B-mediated changes in nuclear PI can stimulate and repress the expression of specific genes through the TAF3-PI interaction site.

### PI Interaction Modulates TAF3 PHD Finger Interaction with H3K4me3

How might TAF3-PI interaction mediate transcriptional regulation? TAF3 regulates gene expression through its interaction with H3K4me3 (Vermeulen et al., 2007), the myogenic transcription factor MyoD (Deato et al., 2008) and the TFIID complex. However, interaction with these transcriptional coponents was not altered in the KK-TAF3 mutant. Full-length WT-TAF3 and KK-TAF3 showed similar interactions with H3K4me3 peptides, while DW-TAF3 as expected was compromised in this interaction (Figure S7A). WT-TAF3, KK-TAF3, and DW-TAF3 similarly interacted with TAF10 (Figure S7B), which mediates the association of TAF3 with the canonical TFIID complex. Finally, both WT-TAF3 and KK-TAF3 interacted similarly with MyoD (Figure S7C) as assessed with co-immunoprecipitation (coIP).

**Figure 7:**
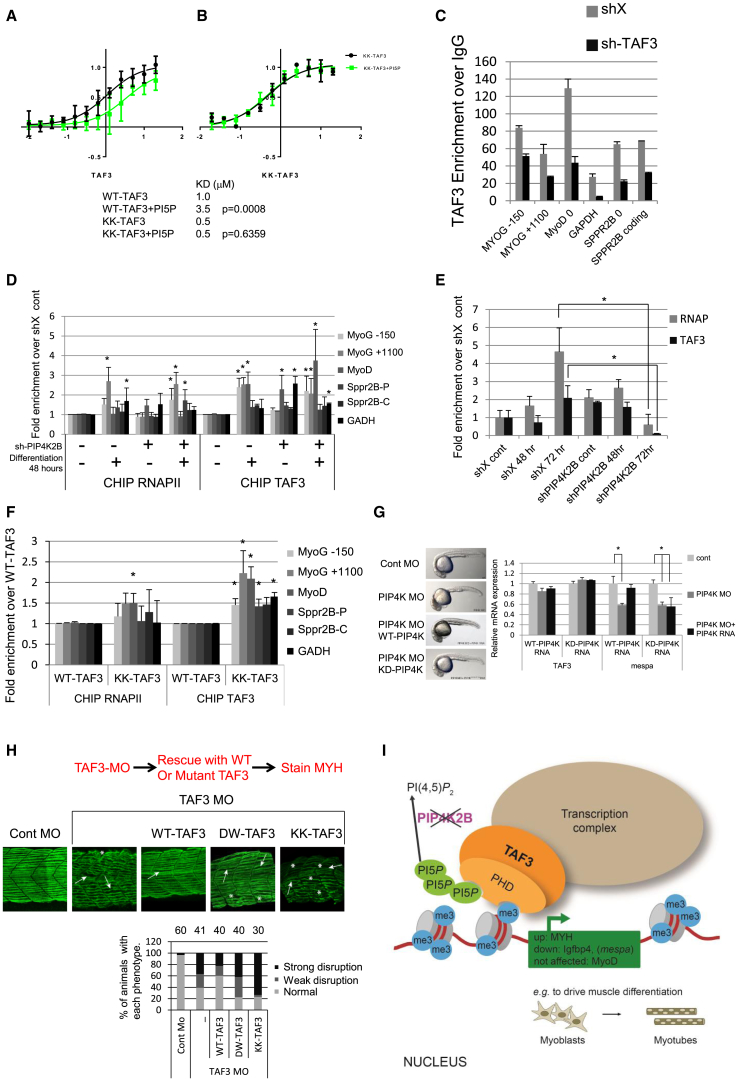
PI Modulates the Interaction of TAF3 PHD Finger with H3K4me3 and the Role of a Conserved PIP4K/PI5P/TAF3 in Zebrafish (A and B) Interaction of increasing concentrations of WT-TAF3 (A) and KK-TAF3 (B) PHD finger with fluorescent H3K4me3 peptide in the absence (black line) and presence (green line) of PI5P. The table indicates apparent KD values for the interactions. PI5P significantly (p = 0.0008) changed the KD of the interaction of WT-TAF3 but not KK-TAF3 PHD finger with H3K4me3 peptide. (C) TAF3 ChIP analysis at the genes indicated of shX or sh-TAF3 C2C12 cells. The data are shown as enrichment over the IgG control and represent the mean of triplicates + SD. (D) RNAPII and TAF3 ChIP analysis at the genes indicated of control (−) or sh-PIP4K2B (+) cells before and after differentiation for 48 hr. Data are presented as mean +SEM (RNAPII n = 5 and TAF3 n = 2). The data were analyzed by a one way ANOVA with a post Hoc Dunnets test to compare conditions to the control sample (undifferentiated SHX). ^∗^p < 0.05. (E) RNAPII and TAF3 ChIP analysis at the promoter of the MYH gene of shX or shPIP4K2B C2C12 cells before and after differentiation for 48 hr and 72 hr. Data are presented as means +SEM (n = 2). Data are presented as mean +SEM (RNAPII n = 5 and TAF3 n = 2). The data were analyzed with one-way ANOVA. ^∗^p < 0.05. (F) C2C12 cells depicted in Figure 6A were analyzed by ChIP for the presence of RNAPII and TAF3 at the genomic regions indicated after differentiation for 48 hr. The data are fold changes over the WT-TAF3 sample and are presented as means +SEM (n = 2). The data were analyzed with one-way ANOVA. ^∗^p < 0.05. (G) Zebrafish embryos injected with control MO or PIP4K targeting MO. PIP4K MO injected embryos were also co-injected with RNA encoding the wild-type human PIP4K2A (WT-PIP4K) or the kinase inactive enzyme (KD-PIP4K). Embryos were collected 24 hr post-fertilization. Left: representative images are shown. Right: QRT-PCR analysis of mRNA isolated from injected embryos for TAF3 or *mespa* (a direct TAF3 target) expression. The data represent fold changes compared to control and represent the mean of triplicates +SD, and were normalized to GAPDH. (H) Zebrafish embryos injected with control MO or TAF3 targeting MO. TAF3 MO injected embryos were also co-injected with RNA encoding human WT-TAF3, or mutant TAF3 constructs unable to interact with PI (KK-TAF3) and methylated histone H3 (DW-TAF3). Embryos were collected 24 hr post-fertilization and stained using F59 (MYHC). Representative images of the disruption of the myosin filament architecture by the indicated injections are shown. The severity of the phenotypes was categorized into strong and weak and presented graphically. The number of injected embryos is indicated above each graph. (I) Schematic showing that interaction of TAF3 with nuclear PI regulated by PIP4K2B modulates transcriptional output. PIP4K2B phosphorylates and regulates the levels of nuclear PI5P and a small pool of PI(4,5)P_2_. Knockdown of PIP4K2B increases PI5P levels that interact with and regulate TAF3 transcriptional complexes. PI interaction with TAF3 can lead to both upregulation and downregulation of specific genes, which eventually affects cell fates such as myoblast differentiation. See also Figure S7.

Using fluorescence polarization, we next tested if PI modulates the conformation of the PHD finger. Measurements with labeled H3K4me3 peptide showed that WT-TAF3 PHD finger interacted with H3K4me3 (KD ≈1 μM, Figure 7A) but as expected not with non-methylated peptide (data not shown). Addition of a 5-fold molar excess of PI5P increased the KD to 3.5 μM (Figure 7A). In contrast, the KK-TAF3 PHD finger exhibited a KD of 0.5 μM and showed no change in KD upon addition of PI5P (Figure 7B). Interestingly, PI(4,5)P_2_ which also interacted with TAF3 by SPR, also modulates the interaction of the PHD finger with H3K4me3 (Figure S7D). These data suggest that PI interaction can modulate the interaction of TAF3 with H3K4me3.

To determine if this occurs in vivo, we assessed the promoter occupancy of endogenous TAF3 using chromatin immunoprecipitation (ChIP). Upon differentiation, TAF3 was highly enriched at PIP4K2B regulated genes (MYOG and Sprr2b) and other genes (MYOD and GAPDH), and knockdown of TAF3 validated antibody specificity (Figure 7C). Differentiation modestly increased both RNA polymerase II (RNAPII) and TAF3 occupancy at the MYOG promoter and gene body as well as at the MyoD promoter. Occupancy of RNAPII and TAF3 after differentiation was not altered by PIP4K2B knockdown (Figure 7D). There was a modest significant increase in TAF3 occupancy at the MyoD and GAPDH promoter in undifferentiated conditions suggesting that nuclear PI might stimulate TAF3 binding in the absence of differentiation (Figure 7D). TAF3 and RNAPII occupancy was also determined at later points of differentiation at both the MYH (Figure 7E) and MYOG promoter (Figure S7E). RNAPII and TAF3 occupancy increased during differentiation and there was a modest increase in TAF3 binding in PIP4K2B depleted undifferentiated cells. Strikingly, 72 hr after differentiation, TAF3 occupancy was strongly decreased in PIP4K2B-depleted cells at both the MYH and the MYOG promoter, suggesting that PI negatively regulates chromatin interaction of TAF3 in vivo. To determine if PI interaction alone could regulate TAF3 in vivo, we assessed the occupancy of KK-TAF3 compared to WT-TAF3. KK-TAF3 occupancy was significantly increased at all genomic regions (Figure 7F), directly linking TAF3 chromatin occupancy with PI interaction.

### PIP4K Regulates TAF3-Mediated Gene Expression in Zebrafish

To determine whether a PIP4K/PI/TAF3 pathway is evolutionarily conserved, we analyzed its function in Mespa expression and on muscle morphology in zebrafish (*Danio rerio*). Mespa is an essential transcription factor in primitive hematopoiesis initiation and is a direct target of TAF3. TAF3 binds the promoter of the *mespa* gene and TAF3 depletion reduces *mespa* expression and attenuates hematopoiesis (Hart et al., 2009). Alignment of the sequence of *taf3* from different organisms showed that residues within the PBR region that are crucial for PI interaction are highly conserved (Figure S7F). Depletion of PIP4K in zebrafish embryos (Elouarrat et al., 2013) led to a developmental phenotype that was suppressed by the co-injection of mRNA coding for a kinase active PIP4K but not a kinase inactive enzyme (Figure S7G), suggesting that regulation of PI underlies in part the phenotypic defect of PIP4K depletion. At 1 day post fertilization (dpf), depletion of PIP4K did not induce gross morphological abnormalities (Figure 7G) nor did they affect TAF3 expression levels. PIP4K depletion however, significantly decreased the expression of *mespa*. The decrease in *mespa* was rescued by the co-expression of kinase active PIP4K but not by the inactive PIP4K, strongly suggesting that *mespa* expression is regulated by changes in PI (Figure 7G). The expression of early hematopoiesis markers (*scl* and *lmo2*) was also decreased in PIP4K-depleted zebrafish (Figure S7H) in line with decreased Mespa function.

Although TAF3 has been implicated in C2C12 myoblast differentiation, its role in muscle development in zebrafish is not known. We therefore depleted zebrafish TAF3 and assessed myosin filament architecture by immunostaining. TAF3 knockdown led to a significant disruption of myofibril alignment, somite boundaries, and shape as monitored by MYH staining. The phenotype could be rescued by co-injection of human WT-TAF3 (Figure 7H) but not by the mutants that only maintain H3K4me3 (KK-TAF3) or PI interaction (DW-TAF3). In fact, expression of the KK-TAF3 appeared to enhance the disorganization of myosin filaments. A similar disorganization of myofibril alignment was observed upon depletion of PIP4K in zebrafish (Figure S7I). Furthermore, mutation of *mespa* leads to muscle phenotypes that resemble those for TAF3-depleted embryos (D.H., unpublished data). These data support a role for a PIP4K/TAF3/mespa pathway in the regulation of zebrafish muscle development and strongly implicate nuclear PI in transcriptional regulation during development in vivo.

## Discussion

Levels of nuclear PI change in response to cell-cycle progression (Clarke et al., 2001), differentiation (Divecha et al., 1995), and in response to stress signaling (Jones et al., 2006) in both animals and plants (Meijer et al., 2001; Ndamukong et al., 2010) and are transduced into functional outputs by their interaction with proteins. Their interactors have implicated roles for nuclear PI in regulating histone modification (Ndamukong et al., 2010), chromatin binding (Gozani et al., 2003; Jones et al., 2006; Gelato et al., 2014), mRNA polyadenylation (Mellman et al., 2008), and topoisomerase activity (Lewis et al., 2011). Here, we show that PIP4K2B modulates C2C12 differentiation and muscle-specific gene expression. Detailed analysis revealed a pathway that directly links PIP4K2B and nuclear PI to the regulation of TAF3, a component of the basal transcription complex.

The effect of PIP4K2B knockdown on C2C12 myoblast differentiation is notable because PIP4K2B is highly expressed in muscle tissue. C2C12 cells resemble activated satellite like cells that are poised to differentiate to repair muscle after injury. This might implicate the PIP4K2B/TAF3 pathway in regulating satellite cell function and muscle biology in vivo. In zebrafish, we show that compromising the interaction of TAF3 with either H3K4me3 or PI and depleting PIP4K affects muscle fiber alignment in vivo. We suggest that this may occur through the regulation of *mespa* because both TAF3 and PIP4K regulate its expression and mutating *mespa* confers a similar phenotype (D.H., unpublished data).

Previous studies in human cells demonstrated that while 43% (11,000 genes) of protein-coding genes are bound by TAF3, the transcription of only 119 genes is affected by TAF3 knockdown, whereas after doxorubicin treatment thousands of genes are affected (Lauberth et al., 2013). A similar effect was observed during differentiation (Liu et al., 2011). This strongly suggests that signal-induced regulation of TAF3 is essential for TAF3 to modulate gene transcription. While changes in H3K4me3 are clearly important for TAF3 signaling, we propose that changes in nuclear PI are another such relevant signal. Our studies in C2C12 cells reveal at least four different groups of genes that are regulated by the PI interaction site of TAF3: group 1 increase on PIP4K2B depletion and is suppressed by loss of the TAF3 PI interaction site (e.g., MYOG); group 2 is downregulated by PIP4K2B depletion and increased by loss of the PI interaction site (e.g., Tspan7); group 3 is partly decreased by PIP4K2B depletion and more strongly suppressed by loss of the PI interaction site (e.g., Igfbp4), and group 4 is not regulated by PIP4K2B depletion but is regulated by PI interaction. A simple hypothesis that might give account for these groups is that 1 and 2 are regulated by PI5P, the substrate of PIP4K2B, whereas group 3 is positively regulated by PI(4,5)P_2_, the product of PIP4K2B activity. Because the pool of PI(4,5)P_2_ regulated by PIP4K2B is likely to be small compared to total nuclear PI(4,5)P_2_ this would likely require additional mechanisms to target TAF3 to this small pool such as its interaction with PIP4K2B. Group 4 is likely to be regulated by other nuclear PI that are not influenced by PIP4K2B depletion such as PI4P. Groups 1 and 2 suggest that nuclear PI can both positively and negatively affect TAF3-mediated gene expression through a single interaction site.

PI modulates the interaction of TAF3 PHD finger with H3K4me3 in vitro and in vivo. However, chromatin occupancy cannot be the sole driver of PI-regulated gene expression because KK-TAF3 has increased occupancy at promoters but actually shows decreased transcriptional output. The interaction of TAF3 with H3K4me3 is also affected by other histone signals such as H3R2 methylation, H3K9 acetylation (Vermeulen et al., 2007), and H3T3 phosphorylation (Varier et al., 2010), and PI interaction may differentially affect how TAF3 interacts with these combinatorial modifications. The observed switch between predominantly negative regulation of TAF3-mediated transcription (during etoposide treatment) to both positive and negative regulation during myoblast differentiation might implicate a role for PI in regulating different TAF3 complexes. Myoblast differentiation switches TAF3-mediated transcription from the canonical TFIID complex to a simpler TAF3/TRF3 complex (Deato and Tjian, 2007). TAF3 also regulates transcription through modulating chromatin looping. Thus, positive and negative effects of nuclear PI on gene transcription might reflect their differential influence on different TAF3 complexes. Furthermore, we suggest that nuclear PI exist as proteolipid signaling platforms (Shah et al., 2013; Blind et al., 2012) that function as organizing centers to recruit chromatin regulators and other enzymes that coordinate pathway specific gene transcription. Therefore, TAF3-mediated changes in transcriptional output in response to changes in PI may depend on the context of other PI interacting chromatin regulators. Nuclear PI platforms may also explain why not all TAF3-dependent genes are influenced by nuclear PI. For example, MyoD is strongly regulated by TAF3 but its expression levels are not influenced by either PIP4K2B depletion or by loss of the PI interaction site on TAF3. This group of genes may not be localized within a nuclear PI platform and thus might not be influenced by TAF3-PI interaction.

PIP4K2B depletion deregulated more genes than the loss of the PI interaction site of TAF3, suggesting that nuclear PI can also regulate gene expression independently of TAF3. 17 of 32 PHD fingers showed PI interaction, implicating nuclear PI in regulation and interpretation of histone modifications (e.g., PHF6, NSD1, MYST4, BAZ1B, TAF3, CXXC1, ING3, ING4, and UHRF1). Furthermore, PI-PHD finger interaction may explain the requirement for developmental switches in PHD finger proteins (Lessard et al., 2007) as well as the role of mutations in cancers. For example, PHF6 is mutated in the human disease Börjeson–Forssman–Lehman syndrome (Lower et al., 2002) and in human leukemia (Van Vlierberghe et al., 2010). In both conditions, nonsense mutations downstream of the second PHD finger have been found that are unlikely to disrupt the PHD finger structure but would delete a PBR. We predict that these mutations would also disrupt interaction with phosphoinositides observed in this study.

In conclusion, this study reveals a pathway linking the regulation of nuclear PI directly to a core promoter component of the transcriptional machinery and further highlights how TAF3 can act to transduce signaling inputs to modulate transcriptional output.

## Experimental Procedures

### C2C12 Maintenance and Differentiation

C2C12 cells were routinely cultured in F12-HAMS medium in 10% fetal calf serum. RNAi knockdown was accomplished using either retroviral (pRetrosuper) or lentiviral transduction (PLKO1 or 2). Cells overexpressing kinase active or inactive PIP4K2B were generated by retroviral transduction (pBabe); 300,000 cells were plated in six-well plates, and the next day differentiation was initiated by switching the cells into F12-HAMS medium containing 2% horse serum. Medium was replaced every 2 days. Cells were washed free of medium and either used in immunofluorescence studies, lysed in SDS-loading buffer for analysis by western blotting or RNA was isolated using RNAeasy and used for QRT-PCR and microarray analysis (accession number GSE66353).

### Phosphoinositide Analysis

Nuclei were isolated from control and differentiated cells and PI5P was analyzed as previously described (Jones et al., 2013) and its levels were normalized to the total amount of nuclear phospholipid phosphate. Other PIs were measured using mass spectrometry (Clark et al., 2011).

PHD fingers were identified using the SMART module and fragments were amplified with Phusion polymerase using specific primers (available on request) and cloned in frame with GST (pGEX-4T1). Proteins were purified and assessed for lipid interaction and for interaction with histone tails. Purified WT and mutant TAF3 PHD finger was also analyzed for interaction with H3K4me3 in the presence and absence of different PI using fluorescence polarization (Gelato et al., 2014).

### ChIP Analysis

TAF3 and RNAPII ChIP assays were performed either as described elsewhere (Lauberth et al., 2007) or using the Diagenode high cell ChIP kit. Immunocomplexes were eluted, crosslinks reversed, and DNA was purified using DNA spin columns. qPCR was performed to measure relative amounts of ChIP DNA (primer details available on request).

### Zebrafish Studies

Zebrafish were handled in compliance with local animal welfare regulations and were maintained according to standard protocols (http://zfin.org). The culture was approved by the local animal welfare committee of the University of Leiden and all protocols adhered to the international guidelines specified by the EU Animal Protection Directive 2010/63/EU or using standard methods at the UCSF CVRI zebrafish facility conducted in conformity with UCSF IACUC and AAALAC guidelines.

The use of the morpholino (3.5 ng MO2) and characterization of the phenotypes associated with knockdown of PIP4K has been previously described (Elouarrat et al., 2013). RNA was isolated using RNA easy kit (QIAGEN) and analyzed by qRT-PCR using suitable primers (available on request). Morpholinos designed to inhibit translation of zebrafish TAF3 and PIP4K have been previously described (Elouarrat et al., 2013; Hart et al., 2009). Morpholinos were microinjected into one cell stage WT zebrafish embryos, 3.5 ng per embryo of PIP4K MO, and 2.5 ng per embryo of TAF3 MO. One nanoliter of 50 ng/ul working solutions of DNA constructs were injected per embryo in rescue experiments.

Immunohistochemistry was performed in whole embryos according to standard protocols. Antibodies used were mouse anti-MyHC F59 (1:30, DSHB), mouse anti-MyHC, MF-20 (1:20, DSHB), and Alexa fluor 488 goat anti-mouse IgG (Life Technologies, 1:400) (secondary). Microscopy on fixed and stained zebrafish was carried out using a Leica DM 5500 microscope.

## Author Contributions

S.-B.Y. performed the initial PHD interaction screen, experiments to determine the role of PIP4K2B and its relationship with TAF3 in myoblast differentiation, and the TAF3 ChIP. L.S. performed SPR analysis of PHD fingers, characterized the interaction of TAF3 with PI and its role in differentiation, and edited the manuscript. M.T., K.A.G., Z.H., and W.F. analyzed the interaction of TAF3 with H3K4me3. M.B. and D.H. analyzed the role of TAF3 in Zebrafish, and P.G., J.v.P., S.-B.Y., and A.-P.G.H. analyzed the role of PIP4K in mespa regulation. H.R., C.T., and S.M.L. provided the TAF3 antibody and carried out the ChIP experiments. K.E.A., D.R.J., and P.H. analyzed nuclear PtdIns levels. The laboratories of W.F., A.-P.G.H., D.H., and S.M.L., contributed equally to this study. N.D. carried out the initial PHD screens, devised and analyzed the experiments, and wrote the manuscript.

## Accession Numbers

The Gene Expression Omnibus accession number for the microarray expression data is GSE66353.

## Supplemental Information

Document S1. Supplemental Experimental Procedures, Figures S1–S7, and Table S1

Document S2. Article plus Supplemental Information
