# Supplementary material for: The Basal Transcription Complex Component TAF3 Transduces Changes in Nuclear Phosphoinositides into Transcriptional Output
Source: Mol Cell. 2015 May 7;58(3):453–67. doi: 10.1016/j.molcel.2015.03.009 (PMC4429956; doi:10.1016/j.molcel.2015.03.009)
Supplement: Document S1. Supplemental Experimental Procedures, Figures S1–S7, and Table S1 [file mmc1.pdf]

Molecular Cell, Volume *58*

Supplemental Information

The Basal Transcription Complex Component TAF3 Transduces Changes in Nuclear Phosphoinositides into Transcriptional Output

Yvette Stijf-Bultsma, Lilly Sommer, Maria Tauber, Mai Baalbaki, Panagiota Giardoglou, David R. Jones, Kathy A. Gelato, Jason van Pelt, Zahid Shah, Homa Rahnamoun, Clara Toma, Karen E. Anderson, Philip Hawkins, Shannon M. Lauberth, Anna-Pavlina G. Haramis, Daniel Hart, Wolfgang Fischle, and Nullin Divecha

figure S1 A

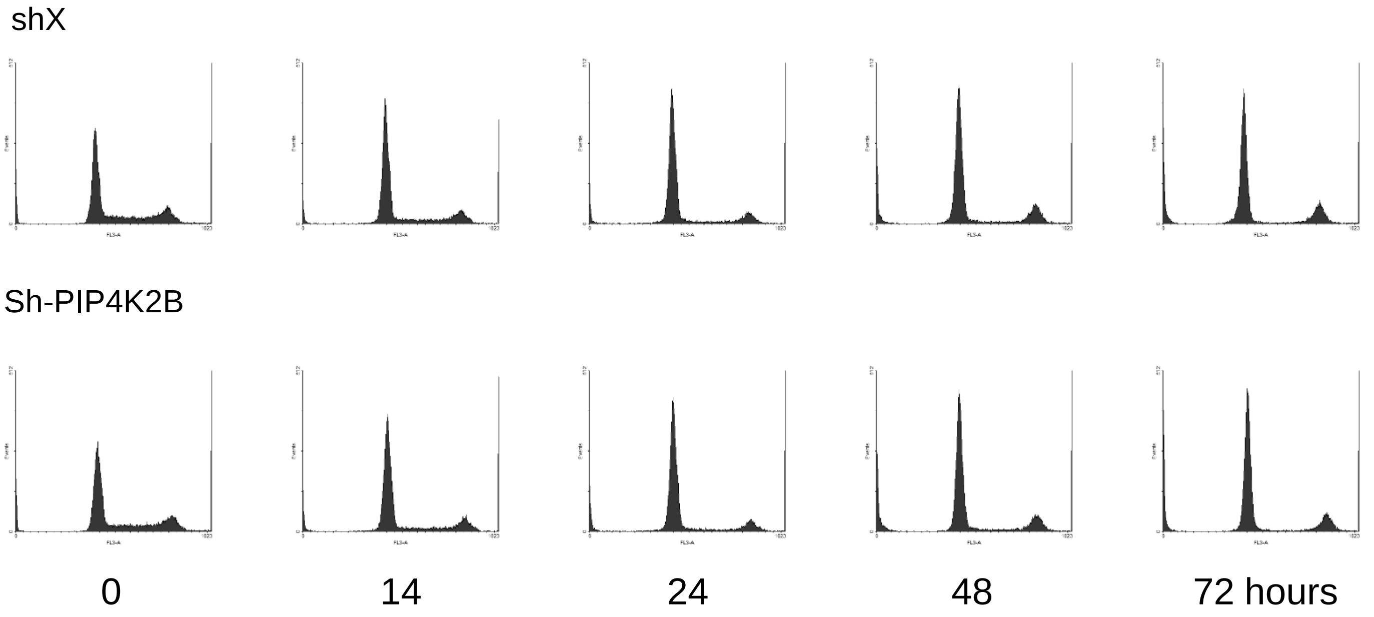

figure S1 B

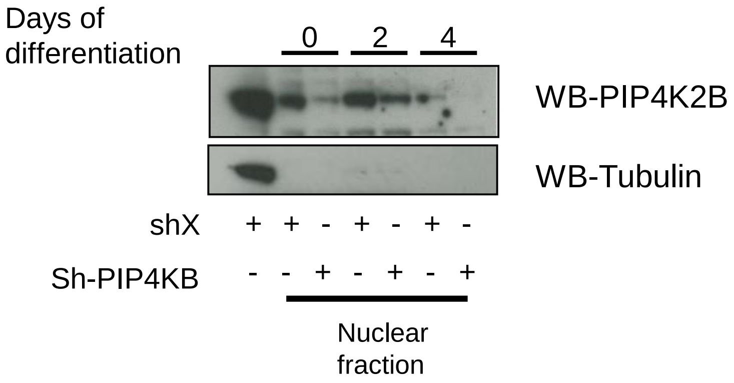

figure S1 C

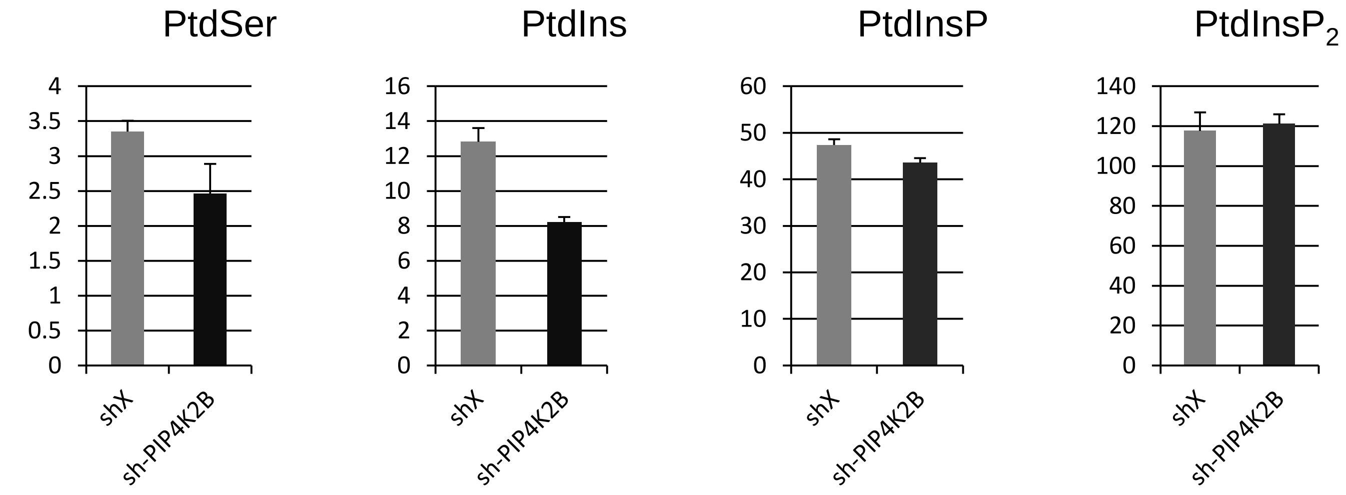

figure S1 D

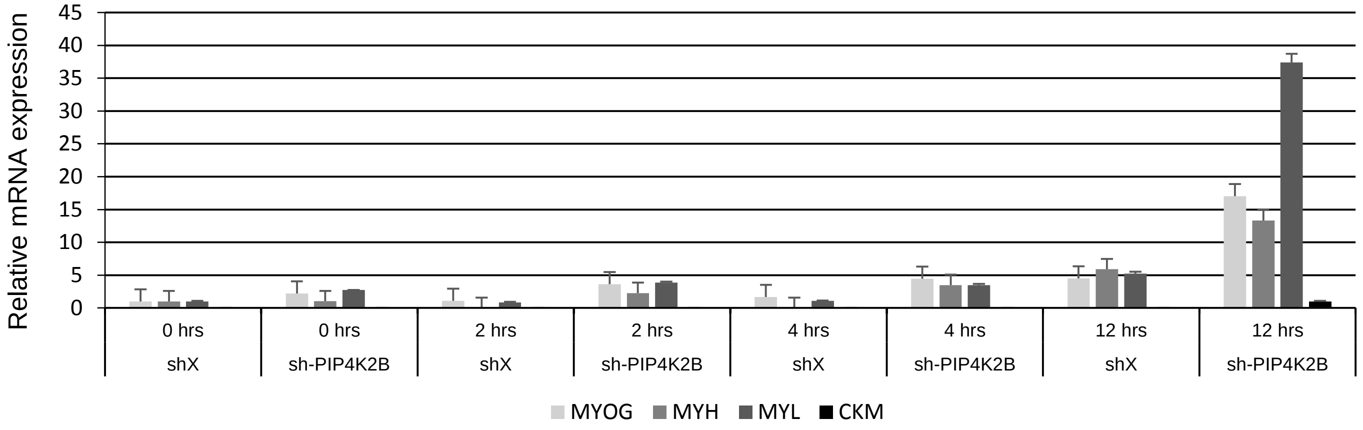

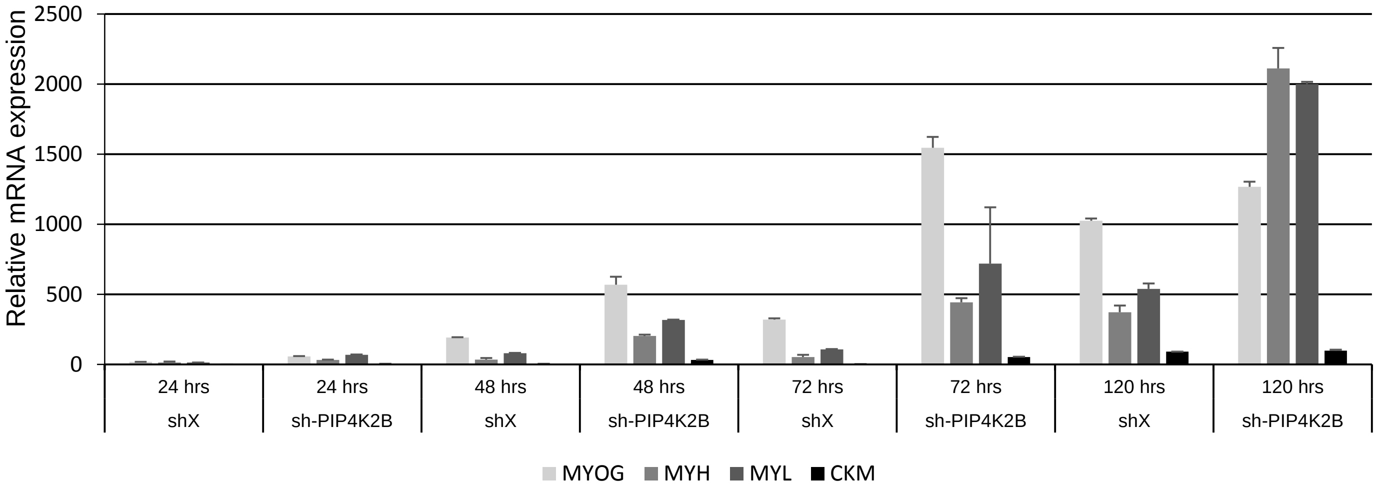

figure S1 E

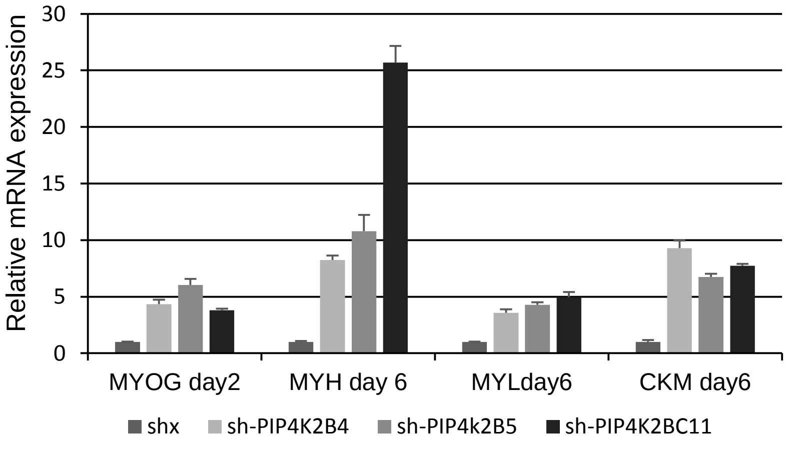

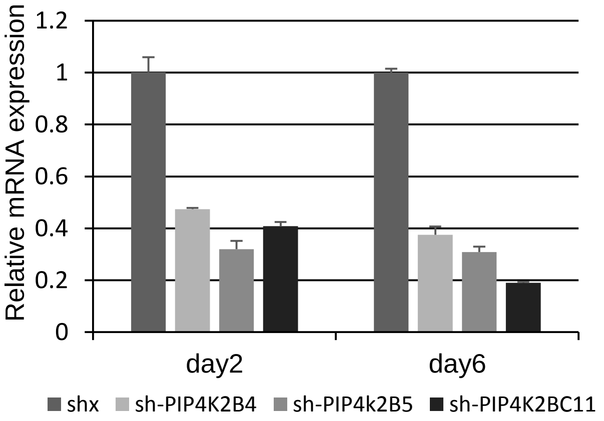

figure S1 F

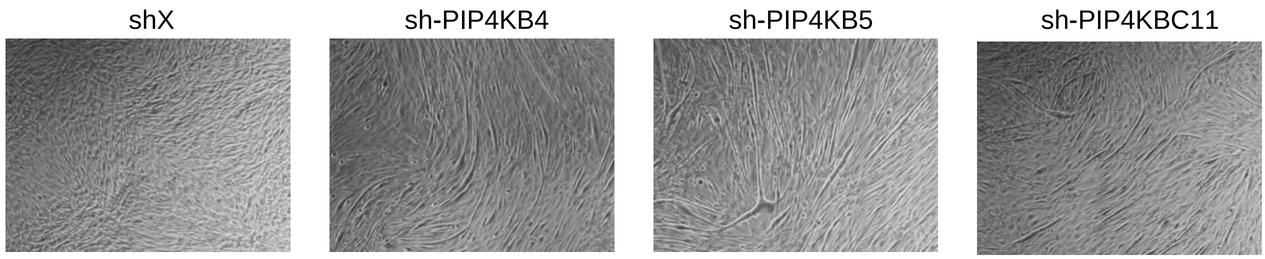

figure S1 G

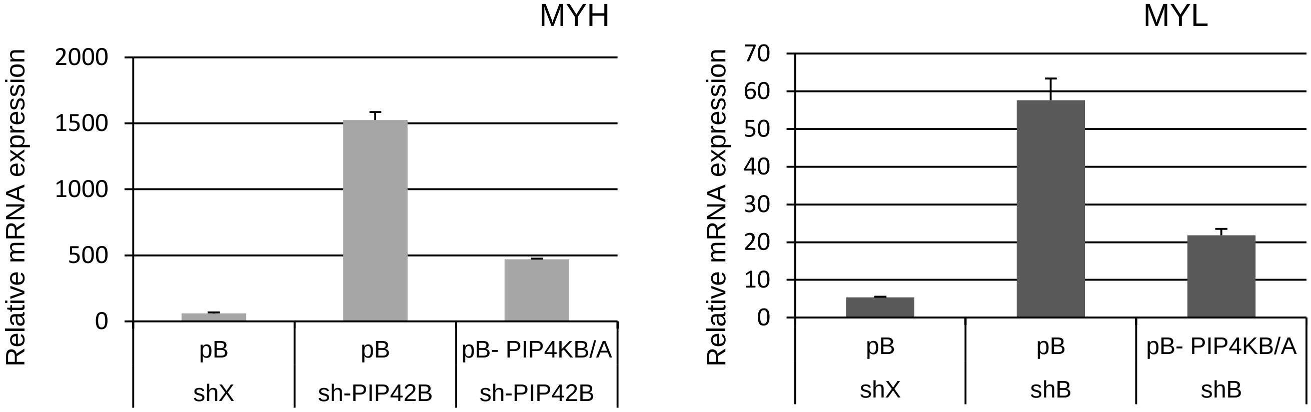

figure S1 H

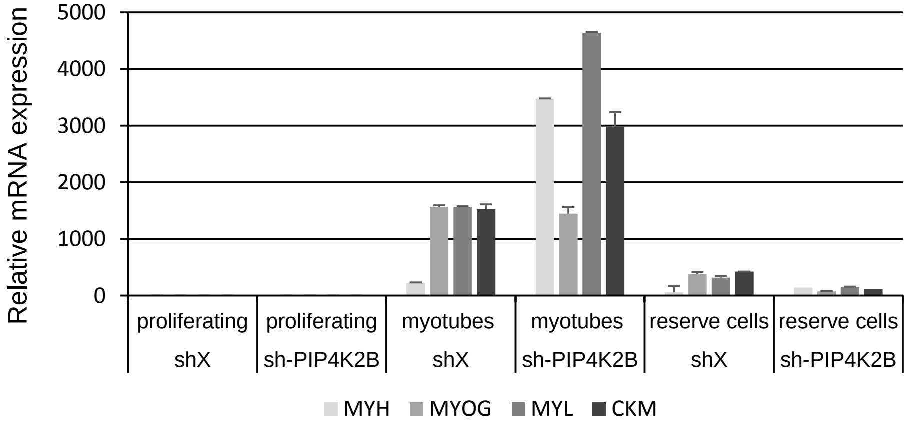

Figure S2

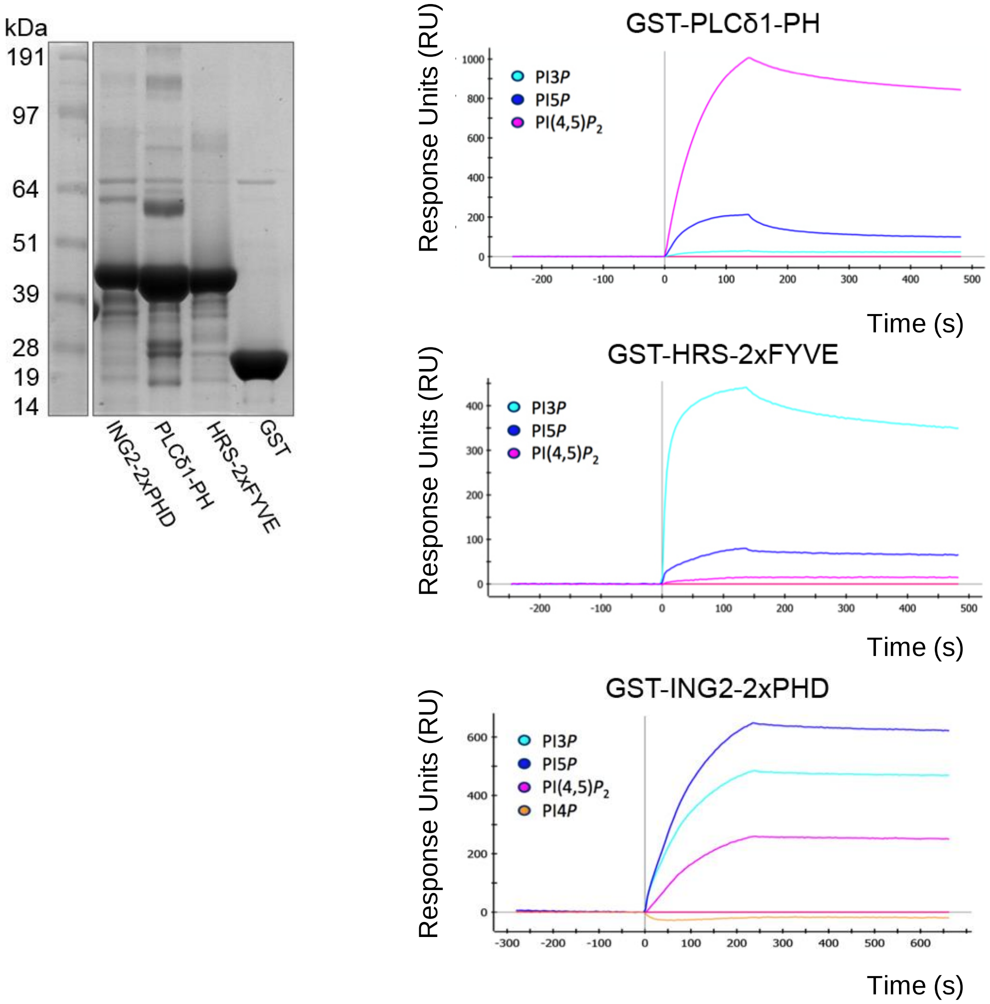

kDa
191
97
64
51
39
28
19
14
ING2-2xPHD
PLCδ1-PH
HRS-2xFYVE
GST
GST-PLCδ1-PH
Response Units (RU)
PI3P
PI5P
PI(4,5)P2
Time (s)
GST-HRS-2xFYVE
Response Units (RU)
PI3P
PI5P
PI(4,5)P2
Time (s)
GST-ING2-2xPHD
Response Units (RU)
PI3P
PI5P
PI(4,5)P2
PI4P
Time (s)

Figure S2A

	PHD-TAF3	PBR
Zinc-finger mutant (C2/H5/C4/C7)	SAKAPVRSVVTETVSTYVIRDEWGNQIWICPGSNKPDDGSPMIGCDDSDDWYSWPCVGIMAAPP EEMQWFCPKSAN	KIKKDKKHKKRKHRAH

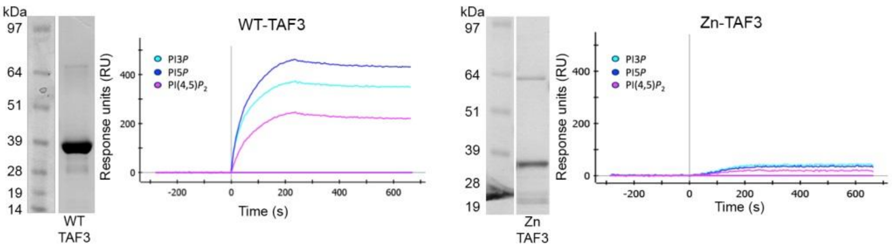

Figure S3 B

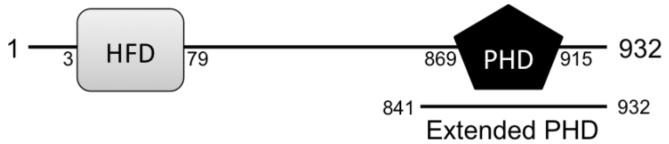

PHD-TAF3 WT

Zinc finger and polybasic region (PBR)

841 S A K A P V R S V V T E T V S T Y V I R
D E W G N Q I W I **C** P G **C** N K P D D G S
P M I G **C** D D **C** D D W Y **H** W P **C** V G I M
A A P P E E M Q W F **C** P K **C** A N K I K K
D K K H K K R K H R A H 932

Figure S3 C

Deletion 1

841 S A K A P V R S V V T E T V S T Y V I R
D E W G N Q I W I C P G C N K P D D G S
P M I G C D D C D D W Y H W P C V G I M
A A P P E E M Q W F C P K C A N 916

Deletion 2

841 S A K A P V R S V V T E T V S T Y V I R
D E W G N Q I W I C P G C N K P D D G S
P M I G C D D C D D W Y H W P C V G I M
A A P P E E M Q W F C P K C A N K I K K 920

Deletion 3

841 S A K A P V R S V V T E T V S T Y V I R
D E W G N Q I W I C P G C N K P D D G S
P M I G C D D C D D W Y H W P C V G I M
A A P P E E M Q W F C P K C A N K I K K
D K K H 924

Deletion 4

841 S A K A P V R S V V T E T V S T Y V I R
D E W G N Q I W I C P G C N K P D D G S
P M I G C D D C D D W Y H W P C V G I M
A A P P E E M Q W F C P K C A N K I K K
D K K H K K R K 928

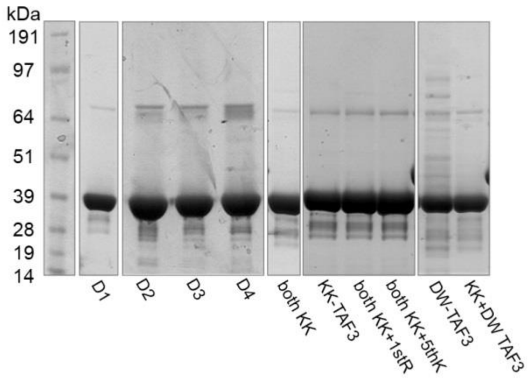

both KK

841 S A K A P V R S V V T E T V S T Y V I R
D E W G N Q I W I C P G C N K P D D G S
P M I G C D D C D D W Y H W P C V G I M
A A P P E E M Q W F C P K C A N K I K K
D A A H A A R K H R A H 932

both KK+5th K

841 S A K A P V R S V V T E T V S T Y V I R
D E W G N Q I W I C P G C N K P D D G S
P M I G C D D C D D W Y H W P C V G I M
A A P P E E M Q W F C P K C A N K I K K
D A A H A A R A H R A H 932

both KK+1st R

841 S A K A P V R S V V T E T V S T Y V I R
D E W G N Q I W I C P G C N K P D D G S
P M I G C D D C D D W Y H W P C V G I M
A A P P E E M Q W F C P K C A N K I K K
D A A H A A A K H R A H 932

PI binding mutant (KK-TAF3)

both KK+1stR+5thK

841 S A K A P V R S V V T E T V S T Y V I R
D E W G N Q I W I C P G C N K P D D G S
P M I G C D D C D D W Y H W P C V G I M
A A P P E E M Q W F C P K C A N K I K K
D A A H A A A A H R A H 932

Histone binding mutant (DW-TAF3)

D890A/W891A

841 S A K A P V R S V V T E T V S T Y V I R
D E W G N Q I W I C P G C N K P D D G S
P M I G C D D C D A A Y H W P C V G I M
A A P P E E M Q W F C P K C A N K I K K
D K K H K K R K H R A H 932

PI- and Histone binding mutant (KK+DW TAF3)

both KK+1stR+5thK+D890A/W891A

841 S A K A P V R S V V T E T V S T Y V I R
D E W G N Q I W I C P G C N K P D D G S
P M I G C D D C D A A Y H W P C V G I M
A A P P E E M Q W F C P K C A N K I K K
D A A H A A A A H R A H 932

Figure S3 D

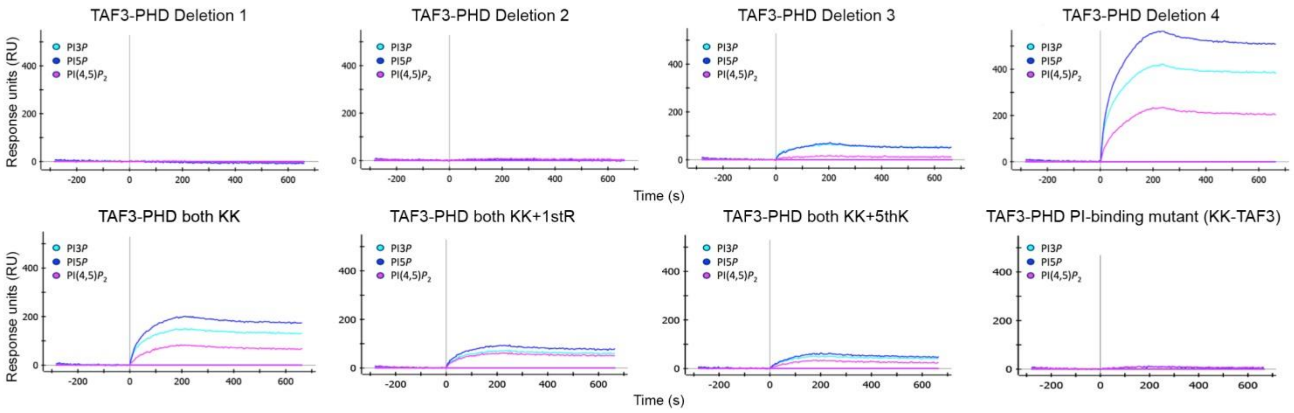

Figure S3 E

GST-PHD-TAF3 proteins

Sequence description	Binding to phosphoinositides (as tested by SPR)	Binding to methylated histone peptides (as tested by SPR)
	referenced to PC	referenced to unmodified H3 (methylation specific binding to H3 peptides)
PHD wild-type	✓✓✓ (very good binding)	✓✓✓ binding to H3K4me3, followed by H3K4me2 and H3K4me1
PHD Deletion 1	✗ (no binding)	✓✓✓
PHD Deletion 2	✗	✓✓✓
PHD Deletion 3	✓ (binding)	✓✓✓
PHD Deletion 4	✓✓✓	✓✓✓
PHD 1st KK mt	✓✓✓	✓✓✓
PHD 2nd KK mt	✓✓✓	✓✓✓
PHD both KK mt	✓✓ (good binding)	✓✓✓
PHD 1stKK+4thK mt	✓✓	✓✓✓
PHD 2nd KK+1stK mt	✓✓	✓✓✓
PHD both KK+5thK mt	✓	✓✓✓
PHD both KK+1stR mt	✓	✓✓✓
PHD both KK+5thK+1stR mt = PI-binding mutant	✗	✓✓✓
PHD M882A	✓✓✓	✗ (no binding)
PHD D886A	✓✓✓	✗
PHD D890A/W891A = Histone-binding mutant	✓✓✓	✗
PHD both KK+5thK+1stR+D890A/W891A = PI- and Histone-binding mutant	✗	✗

Figure S4 A

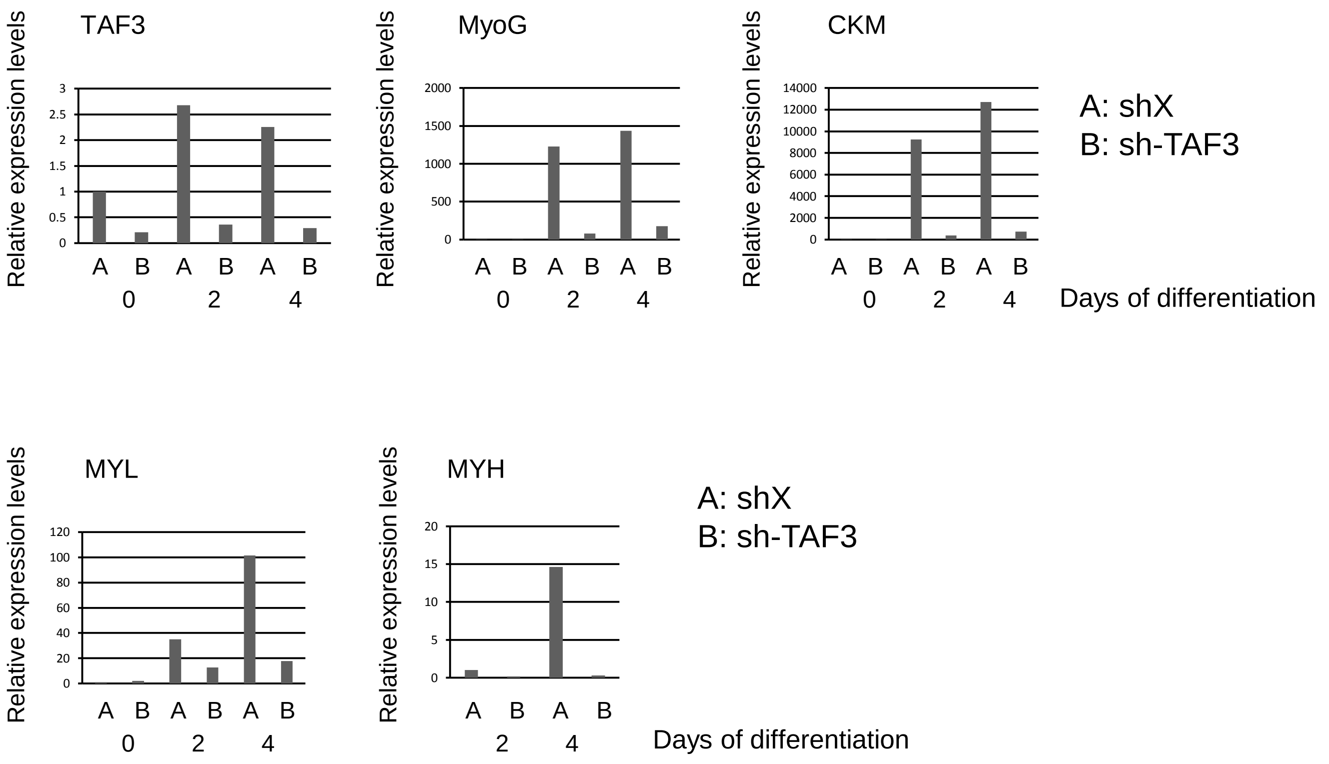

Figure S4 B

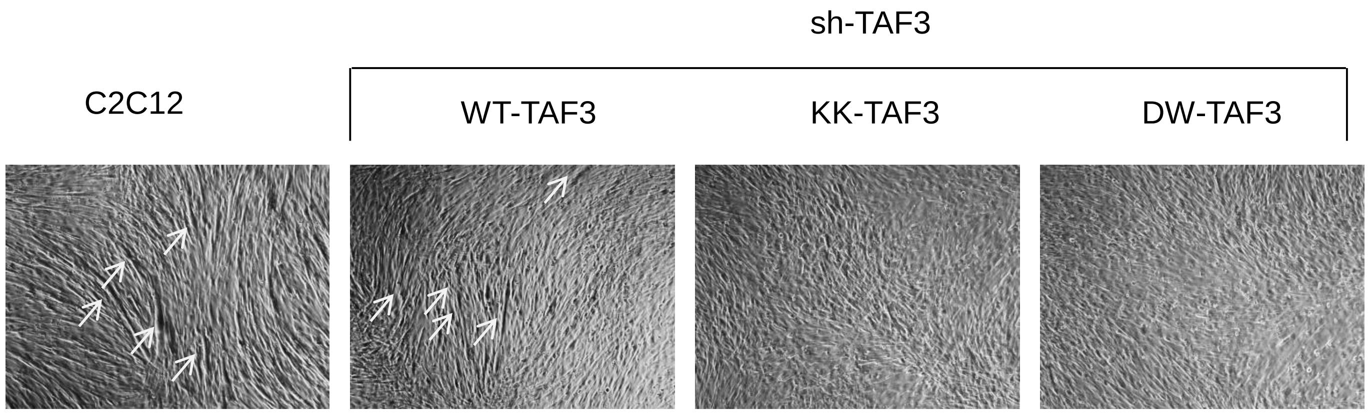

figure S5 A

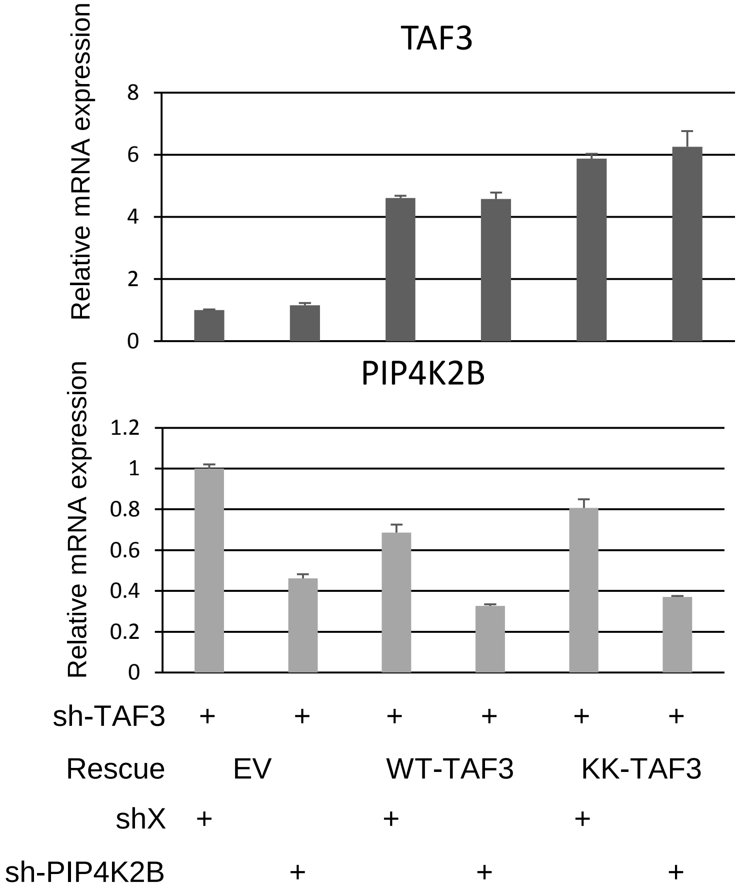

figure S5 B

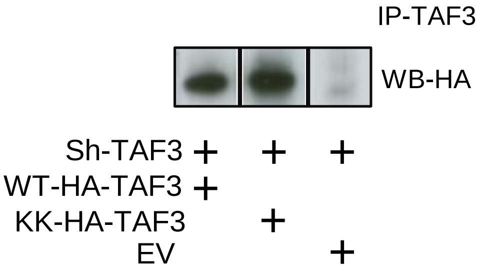

figure S5 C

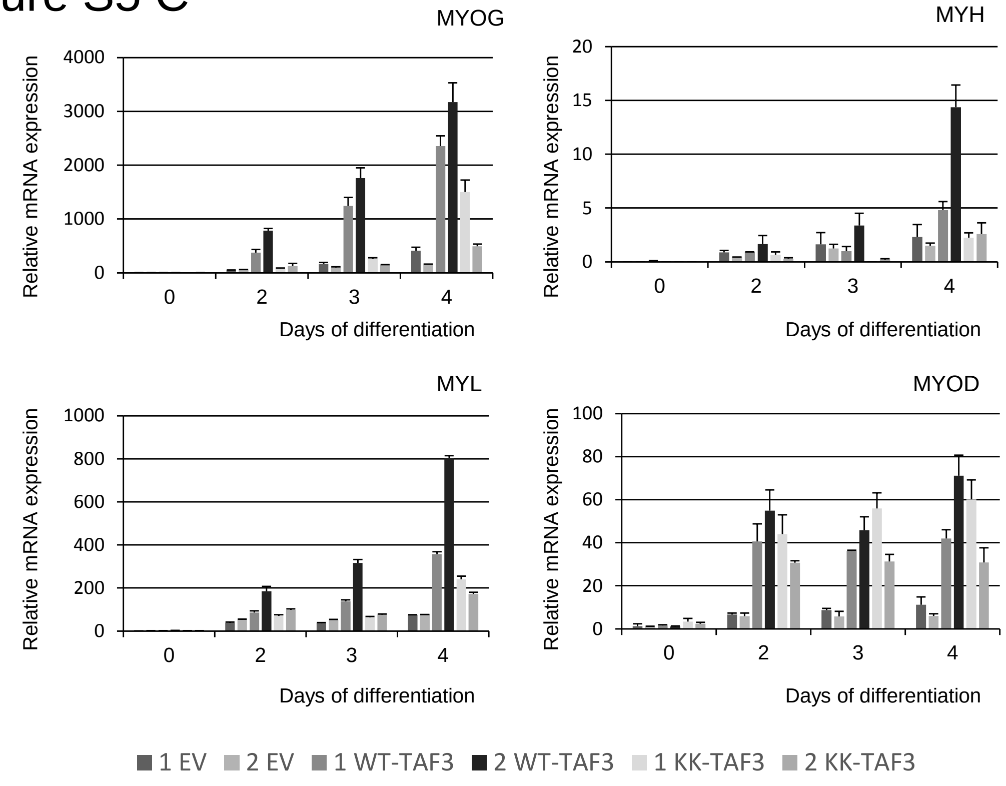

figure S5 D

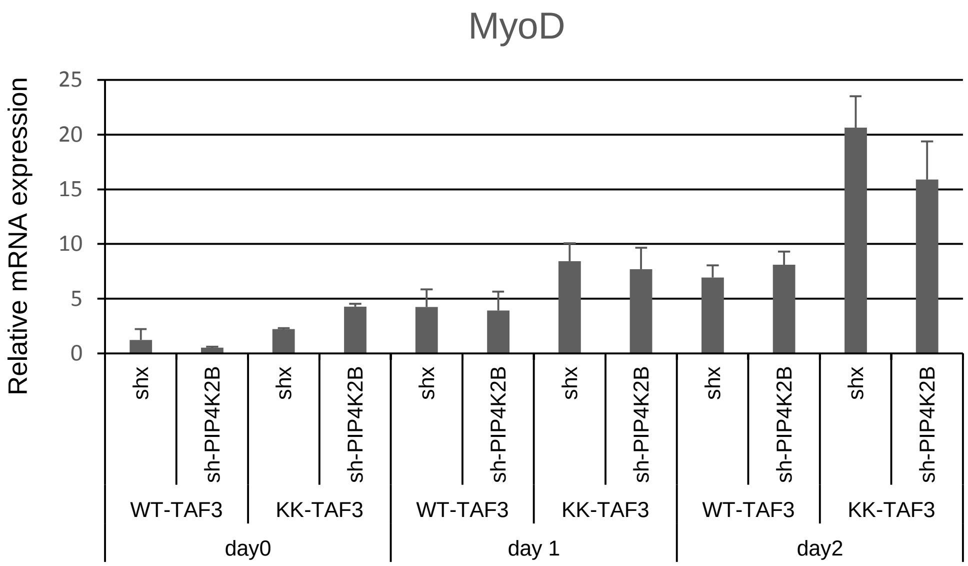
MyoD
Relative mRNA expression
25
20
15
10
5
0
shx
sh-PIP4K2B
WT-TAF3
KK-TAF3
day0
day 1
day2

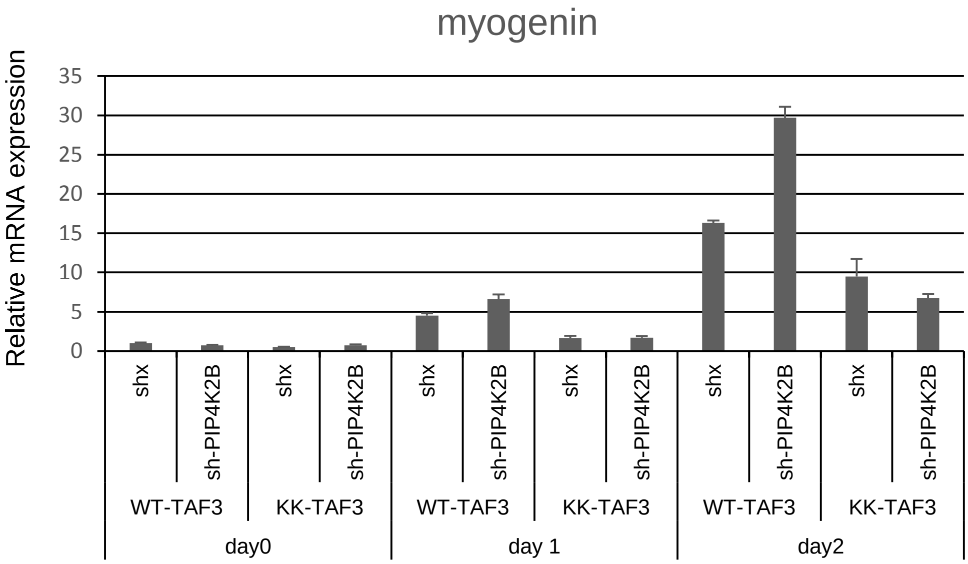
myogenin
Relative mRNA expression
35
30
25
20
15
10
5
0
shx
sh-PIP4K2B
WT-TAF3
KK-TAF3
day0
day 1
day2

figure S6A

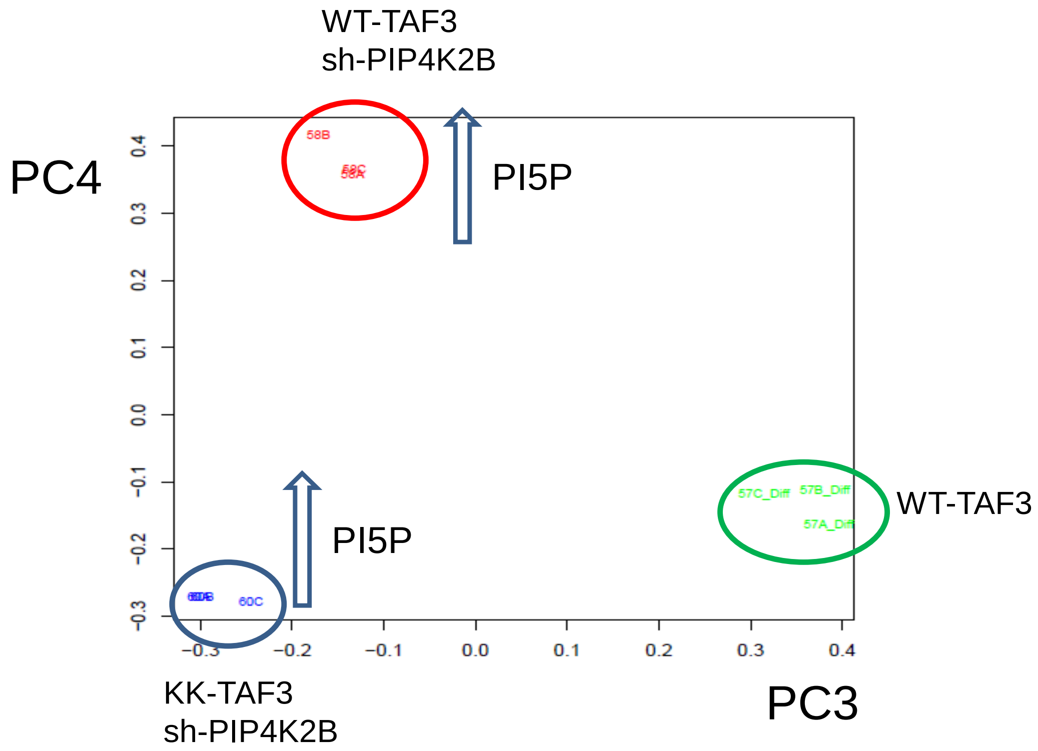

figure S6B

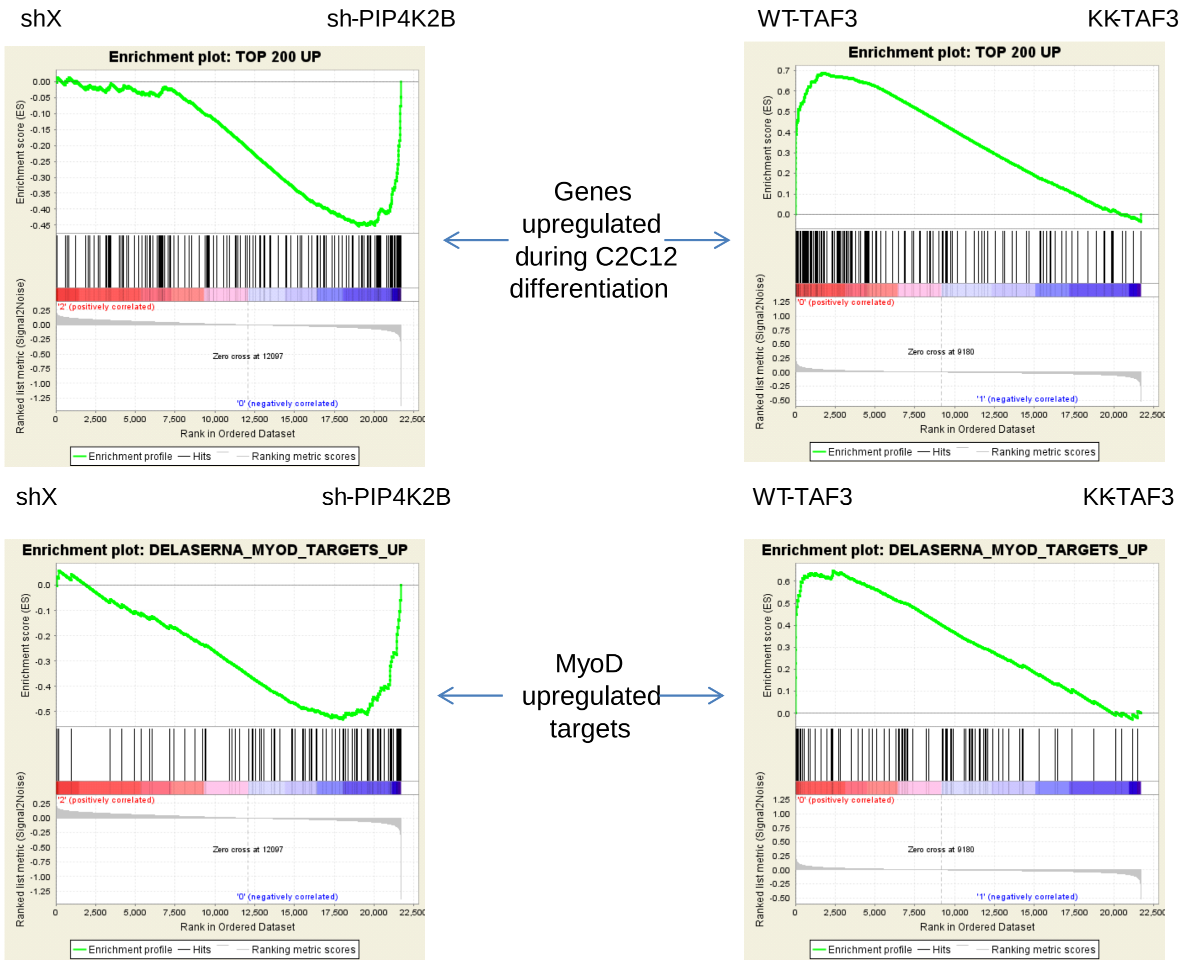

figure S6 C

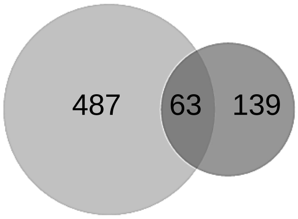

shX v sh-PIP4K2B WT-TAF3 v KK-TAF3

Myoblast differentiation

figure S6 D

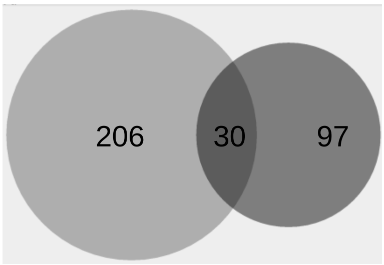

shX v sh-PIP4K2B WT-TAF3 v KK-TAF3

Etoposide

figure S7 A

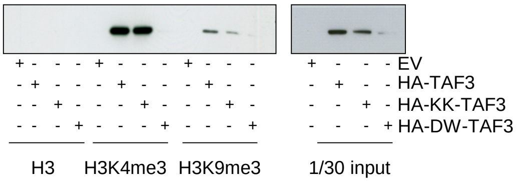

figure S7 B

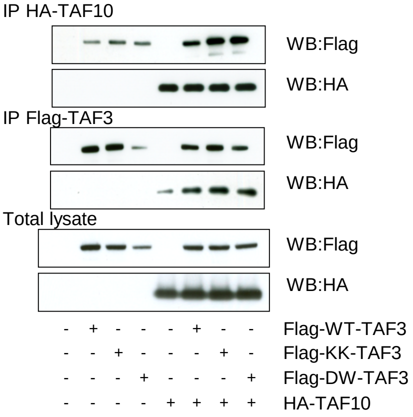

figure S7 C

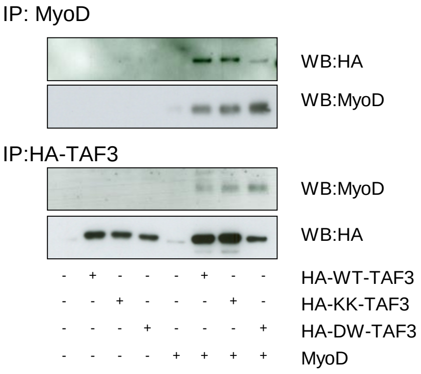

figure S7 D

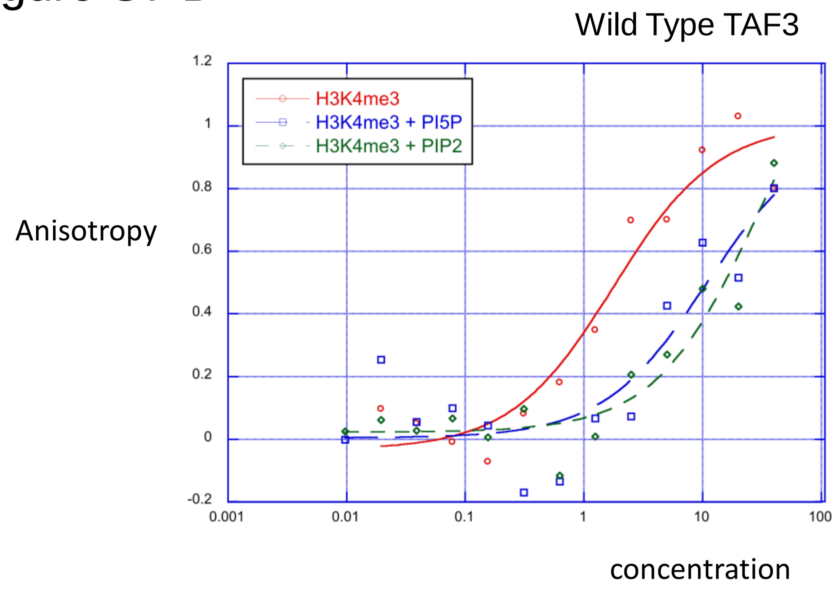

figure S7 E

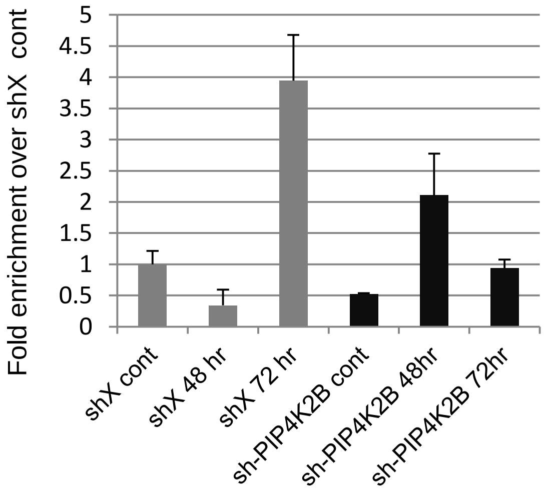

figure S7 F

PBR of TAF3

Danio rerio	882 AGKKKDKKTKKRKRKAH 898
Mus Musculus	915 ANKKKDKKHKKRKHRAH 932
Homo sapiens	913 ANKKKDKKHKKRKHRAH 929
Xenopus laevis	913 ESKKKDKKHKKRKHKAH 929

figure S7 G

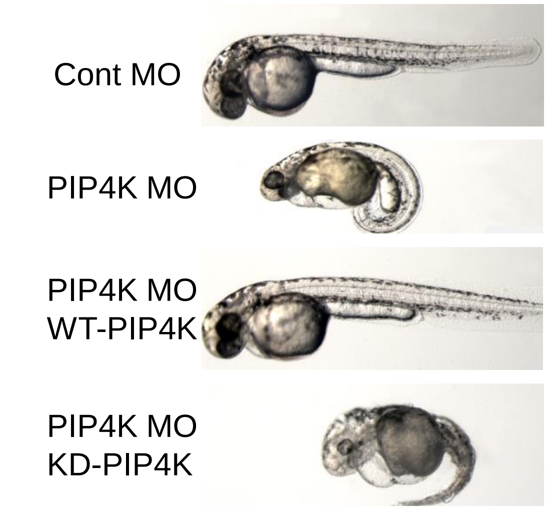

figure S7 H

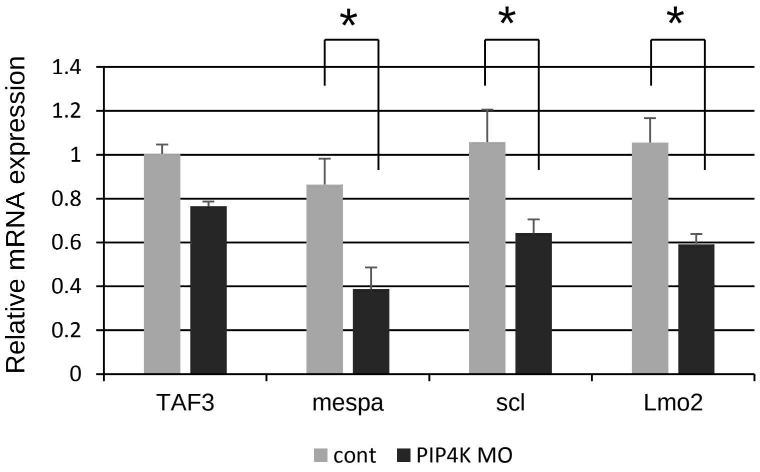

figure S7 I

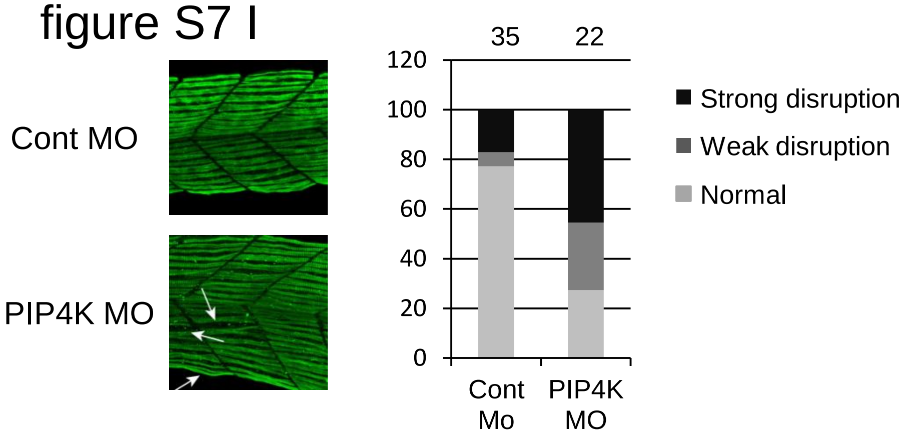

Table S1 Characterisation of the interaction of PHD fingers with phosphoinositides and modified histone tails

Column1	gene name	Function	Domain	expression	interaction with lipid blot	lipid SPR	Preferential Histone tail SPR
1	ARHGEF28	Rho guanine nucleotide exchange factor (GEF) 28	phorbal/dag		x	2	H3 unmodified
2	NSD1	Histone methyltransferase. Preferentially methylates 'Lys-36' of histone H3 and 'Lys-20' of histone H4	PHD4			1	H3 unmodified
3	TRIM28	transcriptional repressor	PHD			0	0
4	DPF2	transcriptional regulation in the BRG1 complex	PHD1 and PH2			0	0
5	KMT2A	Histone methyltransferase	PHD1,2 3	Low		0	H4K4me3
6	KMT2A	Histone methyltransferase	PHD4	Low		0	0
7	PHF1	component of a histone H3 lysine-27 (H3K27)-specific methyl- transferase complex	PHD1,2			0	0
8	KDM4B	Histone demethylase that specifically demethylates 'Lys-9' and 'Lys-36' residues of histone H3	PHD1,2			0	0
9	BAZ2A	Essential component of the NoRC (nucleolar remodeling complex) complex	PHD			1	H3 unmodified
10	PHF20	component of the MOF histone acetyltransferase protein complex	PHD			0	0
11	KDM5A	Histone demethylase that specifically demethylates 'Lys-4' of histone H3	PHD1			0	0
12	INTS12	subunit of the Integrator complex, which associates with the C-terminal domain of RNA polymerase II	PHD			1	H3 unmodified
13	KTM2C	myeloid/lymphoid or mixed-lineage leukemia (MLL) family. Histone methyltransferase. Methylates 'Lys-4' of histone H3	PHD4,5,6	Low		0	0
14	TAF3	Component of the basal transcription complex TFIID	PHD		x	2	H3K4me3
15	KDM4B	Histone demethylase that specifically demethylates 'Lys-9' of histone H3	PHD2	Low		0	0
16	PHF10	component of the neural progenitors-specific chromatin remodeling complex (npBAF complex)	PHD1,2		x	2	H3 unmodified
17	CXXC1	interacts specificity with unmethylated CpG motifs and is a component of the SET1A and B methyltransferase complex	PHD		x	1	equal H3 and H3K4me1,2,3 inhibited by K9 di and trimethylation
18	PHF19	Polycomb group (PcG) that specifically binds histone H3 trimethylated at 'Lys-36' (H3K36me3)	PHD1,2	Low	x	0	0
19	PYGO1	involved inWNT signalling	PHD			0	0
20	PHF14	unknown	PHD2		x	2	H3 unmodified
21	PHF6	mutated in Borjeson-Forssman-Lehmann syndrome (BFLS) and in AML and All	PHD1		x	2	H3 unmodified
22	PHF6	mutated in Borjeson-Forssman-Lehmann syndrome (BFLS) and in AML and All	PHD2	Low	x	0	0
23	ING4	Component of the HBO1 complex which has a histone H4-specific acetyltransferase activity	PHD			1	H3 unmodified
24	TCF20	Transcriptional activator stimulates the activity of various transcriptional activators such as JUN, SP1, PAX6 and ETS1	PHD (ATYPICAL)	Low	x	0	0
25	ING3	a tumor suppressor protein that can interact with TP53 and is a component of the NuA4 histone acetyltransferase (HAT) complex	PHD			1	H3K4me3
26	KAT6B	Histone acetyltransferase component of the MOZ/MORF protein complex	PHD1,2			2	H3 unmodified
27	DIDO1	Putative transcription factor, weakly pro-apoptotic when overexpressed	PHD			0	H3K4me3
28	UHRF1	member of a subfamily of RING-finger type E3 ubiquitin ligases, binds hemimethylated DNA regulates transcription possibly by impacting DNA methylation and histone modifications.	PHD			1	H3 unmodified inhibited by k4 more than k9 methylation
29	BAZ1B	Atypical tyrosine-protein kinase phosphorylating 'Tyr-142' of histone H2AX and plays a central role in chromatin remodeling	PHD			1	0
30	KDM5C	Histone demethylase that specifically demethylates 'Lys-4' of histone H3	PHD1			0	0
31	MTF2	Polycomb group (PcG) that specifically binds histone H3 trimethylated at 'Lys-36' (H3K36me3)	PHD1,2			0	H3 unmodified
32	DPF1	Belongs to the neuron-specific chromatin remodeling complex (nBAF complex)	PHD1,2			1	H3 unmodified

Supplemental Figure legends.

Figure S1 related to Figure 1

A. shX or sh-PIP4K2B C2C12 cells were differentiated for the number of hours indicated. The cells were fixed and stained with propidium iodide and analysed by flow cytometry.
B. shX or sh-PIP4K2B C2C12 cells were differentiated for the times indicated after which nuclei were isolated and analysed by immunoblotting with the indicated antibodies. The first lane shows the presence of the proteins in a total cell lysate.
C. shX or sh-PIP4K2B C2C12 cells were differentiated for two days after which nuclei were isolated and the mass of lipids was determined by mass spectrometry. The data are normalised to an internal standard and represent the mean of triplicates +SD (arbitrary units).
D. shX or sh-PIP4K2B C2C12 cells were differentiated for the times shown and gene expression levels were determined by QRT-PCR as indicated. The data represent fold changes compared to the 0 hour shX sample and represent the mean of triplicates +SD.
E. shX or sh-PIP4K2B C2C12 cells were differentiated for the times shown and gene expression levels were determined by QRT-PCR as indicated. The graph on the right shows the expression of PIP4K2B in the cell lines at the times indicated after differentiation. The data represent fold changes compared to the shX sample and represent the mean of triplicates +SD.
F. C2C12 were depleted of PIP4K2B using three different targeting constructs and representative images of myotube formation are shown. The knockdown efficiency is shown in Figure S1E.
G. shX or sh-PIP4K2B C2C12 cells were transduced with control vector (PB) or with a rescue vector encoding a fusion protein of PIP4K2B /2A. The cells were differentiated for 48h before expression of MYH and MYL was quantitated by QRT-PCR. The data represent fold changes compared to the shX sample and represent the mean of triplicates +SD.
H. shX or sh-PIP4K2B C2C12 cells were differentiated for six days after which myotubes and reserve cell populations were isolated by limited trypsinisation and replating. RNA was isolated and the expression of the indicated genes was determined by QRT-PCR. The data represent fold changes compared to the proliferating shX sample and represent the mean of triplicates +SD.

Figure S2 related to Figure 2

GST fusion proteins with characterised lipid interaction domains were purified and used to determine their interactions with PI presented on a micellar SPR chip surface. GST-PLCδ1 PH domain interacted strongly with PI(4,5)P_2 and the 2XFYVE domain interacted strongly with PI3P as expected. The 2XPHD finger of ING2 showed interaction in this assay with PI5P, PI3P and PI(4,5)P_2.

Figure S3 related to Figure 3

A. The cysteine zinc finger mutant of the PHD finger of TAF3 was analysed for its interaction with PI. Wild type TAF3 PHD finger interacted strongly with PI (left panel) while the zinc finger mutant interaction was severely compromised (right panel). The zinc finger mutant

was also compromised in its interaction with H3K4me3 (data not shown). Residues in red indicate cysteine or histidine that were converted to serine residues.

B. Cartoon depicting the structure of TAF3 and the sequence of the extended PHD finger including the PBR region (highlighted). Bold residues indicate amino acids that constitute the canonical zinc finger interaction residues.
C. Sequences depicting the deletion mutants and mutants within the PBR used to determine exactly how TAF3 PHD finger interacts with PI. The gels show purified protein of the various mutants.
D. Upper panel shows the interaction of the deletion mutants with PI while the lower panel shows a subset of the PBR point mutants and their interaction with PI.
E. The interaction of various mutants with either PI or with histone peptides is summarised in the table. Increasing number of ticks refers to increased strength of interaction, while an X indicates no interaction.

Figure S4 related to Figure 4

A. Control cells (A) or cells with TAF3 knocked down (B) were differentiated for the days indicated after which RNA was extracted and the expressions of the indicated genes (above) were determined using QRT-PCR. The data represent fold changes compared to the 0 shX (A) sample and represent the mean of triplicates +SD.
B. Wildtype or sh-TAF3 C2C12 cells were rescued with wildtype (WT-TAF3) or mutants which only maintain H3K4me3 (KK-TAF3) or PI interaction (DW-TAF3), respectively. Cells were differentiated for 5 days and analysed by widefield microscopy. Arrows denote easily observable myotubes.

Figure S5 related to Figure 5

A. sh-TAF3 C2C12 cells were rescued with either empty vector,WT- or KK-TAF3 and then transduced with a control sh-RNA (shX) or with an sh-RNA targeting PIP4K2B (sh-PIP4K2B). Cells were then differentiated for 3 days before TAF3 or PIP4K2B expression (as indicated) was assessed by QRT-PCR. The data represent fold changes compared to the EV-shX (EV 1) sample and represent the mean of triplicates +SD.
B. sh-TAF3 C2C12 cells were rescued with empty vector (EV) or with WT- or KK-Ha-tagged TAF3. Cell lysates were immunoprecipitated using an antibody against TAF3 and immunoblotted using an antibody against HA.
C. sh-TAF3 C2C12 cells were rescued with either empty vector (EV), WT- or KK-TAF3 and then transduced with a control shX vector (1) or with a sh-RNA targeting PIP4K2B (2). Cells were differentiated for the days indicated before gene expression levels were assessed by QRT-PCR as indicated. The data represent fold changes compared to the 0 day EV-shX sample and represent the mean of triplicates +SD.
D. sh-TAF3 C2C12 cells were rescued with either WT (wt) or KK-TAF3 (kk) and then transduced with a control RNAi vector (shX) or one targeting PIP4K2B (sh-PIP4K2B). Cells were differentiated for the number of days indicated, and expression levels of MYOD or PIP4K2B were analysed by QRT-PCR. The data represent fold changes compared to the 0 day shX sample and represent the mean of triplicates +SD.

Figure S6 related to Figure 6

A. Principal component analysis of the microarray gene expression data using the top 500 most variable genes. Each point represents a single array from sh-TAF3 C2C12 cells rescued with WT-TAF3 (green), WT-TAF3 and depleted of PIP4K2B (red) or KK-TAF3 and depleted of PIP4K2B (blue) (Figure 6A). The analysis shows a high degree of coherence between biological replicates and clearly shows separation of cells based on their genotype. For example, PC3 describes variation dependent on the expression of PIP4K2B but not dependent on the PI interaction site of TAF3. PC4 defines variability that depends on both expression of PIP4K2B and the interaction of TAF3 with PI.
B. Gene set enrichment analysis (GSEA) was used to identify gene expression programmes that are differentially regulated in two different conditions. Enrichment of gene sets is observed as a polarised presence of members of the gene set in a ranked list of expression differences. A gene set containing genes upregulated after myogenic differentiation of C2C12 cells (top panel) and genes induced by MyoD overexpression (bottom panel) were highly enriched in sh-PIP4K2B compared to shX C2C12 cells (left panel) and in WT- compared to KK-TAF3 C2C12 cells (right panel). This data illustrates that knockdown of PIP4K2B compared to control or expression of WT- compared to KK-TAF3 enhance the myogenic gene expression programme during differentiation.
C. Comparative expression analysis identified genes that were regulated by PIP4K2B knockdown or by the expression of KK- compared to WT-TAF3 in sh-TAF3 C2C12 rescue cells after differentiation for two days. The expression levels of 550 genes changed upon knockdown of PIP4K2B ($p<0.05$ and more than 1.4 fold difference), of which 331 were up regulated. 202 genes were differentially expressed in WT- compared to KK-TAF3 sh-TAF3 C2C12 rescue cells (1.4 fold cut off and $p<0.05$). A highly significant number of 63 genes formed the overlap between PIP4K2B- and KK-regulated gene sets (representation factor 11.9 and $p<3.79e^{-50}$). A representation factor above 1 suggests more than the expected overlap. The probability factor is based on exact hypergeometric probability with normal distribution http://nemates.org/MA/progs/representation.stats.html).
D. Comparative expression analysis identified genes that were regulated by PIP4K2B knockdown or by the expression of KK- compared to WT-TAF3 in sh-TAF3 C2C12 rescue cells after etoposide treatment. 236 genes were differentially expressed upon PIP4K2B knockdown, while 127 genes were differently expressed in WT- compared to KK-TAF3 sh-TAF3 C2C12 rescue cells, with a highly significant overlap of 30 genes between the two gene sets (representation factor 21, $p<2.89e^{-31}$).

Figure S7 related to Figure 7

A. HEK293 cells were transfected with TAF3 PHD finger constructs indicated (right) and lysates were used for affinity chromatography with beads coupled to unmodified histone H3 peptide (H3) or modified by trimethylation at lysine 4 (H3K4me3) or at lysine 9 (H3K4me3). Bound proteins were separated by SDS-PAGE and assessed by immunoblotting using an anti-HA antibody. The right panel depicts the input of the various TAF3 proteins.
B. HEK293 cells were transfected as indicated (bottom) and then immunoprecipitated with the antibodies indicated on the left and bound proteins were assessed using SDS-PAGE and immunoblotting with the antibodies indicated on the right. The lower panel shows the expression of the various proteins in the input lysates.

C. HEK293 cells were transfected as indicated (bottom) and immunoprecipitated with the antibodies shown on the left. Bound proteins were assessed by SDS-PAGE and immunoblotting with the indicated antibodies (right).
D. Increasing concentrations of WT-TAF3 PHD finger was assessed for its interaction with fluorescent H3K4me3 peptide in the absence (red line) and presence of PI5P (blue line) and PI(4,5)P_2 (green line). The data demonstrate that both PI5P and PI(4,5)P_2 decrease the interaction between TAF3-PHD finger and H3K4me3.
E. TAF3 Chip analysis at the promoter of the MYOG gene of shX or sh-PIP4K2B C2C12 cells before and after myoblast differentiation for 48h or 72h. The data is represented as fold enrichment over the shX control and are mean+SEM (n=2).
F. An alignment showing the strong evolutionary conservation of the PBR of the TAF3 PHD finger from various organisms.
G. Zebrafish embryos were injected with either a control morpholino (MO) or a PIP4K targeting morpholino (PIP4K MO) with or without RNA encoding the wild type human PIP4K2A (WT-PIP4K) or the kinase inactive enzyme (KD-PIP4K) and were collected 48h post fertilisation. PIP4K MO induces a developmental phenotype observed in approximately 70% of fish. The rescue was observed in 80% of injected fish with WT-PIP4K RNA but only in approximately 10% injected with the mutant kinase inactive PIP4K RNA.
H. Control zebrafish embryos (cont) or embryos injected with 3.5ng PIP4K MO were collected 24h post fertilisation and assessed for the expression of the TAF3 dependent transcription factor *mespa* and for direct Mespa downstream gene targets *scl* and *lmo2*. The data represent fold changes compared to the control samples and represent the mean +SD of triplicates. The data were normalised to the housekeeping gene GAPDH.
I. Zebrafish embryos were injected with a control- or PIP4K MO. Embryos were collected 24h post fertilisation and stained using F59 (MYHC). Representative images of the disruption of the myosin filament architecture by the indicated injections are shown. The severity of the phenotypes were categorised into strong and weak and presented graphically. The number of injected embryos is indicated above the graph.

Table S1 related to figure 2.

Table S1 summarises the interaction of the various PHD fingers with PI as assessed by either lipid dot blot or SPR analysis as well as their interaction with unmodified and methylated histone h3 tails. Column 1 numbers corresponds to numbers under the gel picture in Figure 2A. Of the 32 proteins tested by lipid dot blots, nine interacted strongly with one or more PI and in general interacted with PI3P, PI4P, PI5P and PI(3,5)P_2 (Figure 2B). SPR confirmed the interaction of six of the nine proteins identified using lipid dot blots and identified an additional 17 PHD fingers as PI interactors. None of the PHD fingers interacted with PI(4,5)P_2 when assessed by lipid dot blots but did when assessed using SPR. The expression of some proteins was very low (Low) indicating that they might be unfolded. Interaction assessed by SPR was characterised as strong (2) medium (1) or weak/non-interacting (0). The interaction of PHD fingers with histone tails was referenced to interaction with unmodified H3 tail peptide. H3 unmodified indicates that the PHD finger interacted more strongly with the unmodified H3 tail compared to methylated tails. H3K4me3 indicates preferential interaction with H3K4me3. 0 indicates that very little or no interaction was observed.

Supplemental experimental methods

Quantitative RT-PCR and Microarray analysis. QRT-PCR was carried out using specific primers (available on request) either using internal probes or using SYBR-Green. In the latter case specificity was determined using melting curves. Changes in expression were determined by the $\delta\delta$CT method with samples normalised to the level of GAPDH. Data are presented as the mean +SD of triplicates and all experiments were carried out at least twice. Global gene expression changes were analysed by microarray hybridisation analysis using an Affymetrix 2.0 mouse chips (CRUK Manchester Institute array facility). Data was normalised and gene expression differences were determined using a 1.4 fold threshold and a $p<0.05$.

sh-RNA mediated knockdown: sh-RNA sequences targeting TAF3 were cloned into pRetroSuper and retroviral particles were generated in either Phoenix-ECO or Plat-E cells and used to infect C2C12 cells in the presence of polybrene (5μg/ml) overnight. Cells were washed free of virus and selected with puromycin (5μg/ml). For rescue experiments full length TAF3 (WT, KK or DW) or PIP4K2B were cloned into pBABE and used to generate retroviral particles in either phoenix-ECO or Plat-E cells. C2C12 cells were infected as above and selected accordingly.

Lentiviral sh-RNA: sh-RNA targeting PIP4K2B or a control sequence (shX) cloned in the pLKO 1 or 2 vector were purchased from SIGMA (St Louis, USA). Viral particles were generated in HEK293FT cells using pLKO-based vectors and plasmids encoding GAG-Pol and VSVG (4:2:1, respectively). C2C12 cells were transduced in the presence of polybrene (5μg/ml) and selected using puromycin (5μg/ml).

Immunofluorescence. Staining was carried out using the appropriate antibodies diluted 1:100 after fixation with 4% formaldehyde. Cells were permeabilised using 0.1% TX-100-PBS and non specific sites were blocked using 3% BSA-PBS. Primary antibody incubation was carried out in the 1% BSA-PBS for 1 hour (h) after which cells were washed and incubated in the appropriate secondary antibody diluted 1:200 in 1% BSA-PBS for 1h. After washing, cells were stained with DAPI and mounted using antifade reagent. Immunofluorescence was visualised using a spinning disk confocal microscope (Olympus IX81).

Luciferase assays. C2C12 cells were transfected with luciferase constructs driven by the indicated promoters together with renilla luciferase driven by the thymidine kinase promoter as a control. Cells were differentiated for two days after which the cells were lysed in passive lysis buffer (Promega) and luciferase activities were analysed using the Stop and Glo reagents (Promega). Firefly luciferase counts (promoter specific) were normalised for the renilla counts (control plasmid).

Cloning and purification of GST fusion proteins. PHD fingers were identified using the SMART module and fragments were amplified with Phusion polymerase using specific primers (available on request). Fragments were T-tailed with Taq polymerase and directly cloned into the Sma1 site (previously A-tailed). Successful cloning was assessed by scoring single colonies for protein production and subsequently each clone was verified by sequencing (PHD finger sequences available on request).

Single bacterial colonies were grown in 10ml LB overnight, diluted 20 fold in the morning and grown for another 3h at 37°C. Fusion proteins were induced by the addition of 50μM IPTG and 10μM $ZnSO_4$ and grown overnight or for 4h at 25°C. Bacterial pellets were washed once in PBS before lysis by sonication in 1% TX-100-PBS containing complete EDTA free Roche protease inhibitors. GST proteins were isolated by incubation with glutathione beads (GE Healthcare) for 2h, washed and eluted using 50mM TRIS pH 8.0, 300mM NaCl, 10mM glutathione, 10mM DTT, 10μM $ZnSO_4$. Protein concentration was assessed using BioRad reagent.

Lipid Dot blots. 50pmoles of PI lipid dissolved in chloroform (2μl) were spotted onto nitrocellulose membranes, which were blocked in 3% BSA-TBS before incubation with GST-proteins (0.2μg/ml diluted in 3% BSA-TBS) for 2h. After washing, blots were incubated with an anti-GST-antibody and interaction was visualised using an anti-mouse antibody conjugated to HRP and enhanced chemiluminescence (Super Signal Pierce).

SPR analysis.
Lipid interaction: The sensor surface was conditioned by vertical injections of 0.5% SDS, followed by 50 mM NaOH and 100 mM HCl at 30 μl/min for 60s after which undecylamine was coupled using NHS/EDC to prepare a hydrophobic capture SPR sensor chip. PI (1nmole) or phosphatidylcholine (1nmole) were sonicated into 100μl of 10mM Tris pH 7.4 and diluted in 50mM acetate pH 5.5. Approximately 500RU units were captured on the SPR chip. If necessary, lipids were reloaded to achieve equal loading levels. Recombinant GST-tagged proteins (analyte) were diluted in the respective SPR buffer to the desired concentration (usually 100nM to 1000nM). Analyte injections were generally performed at a flow rate of 50 μl/min (sometimes 100 μl/min) and 25°C sensor chip temperature. Association of analyte molecules to the lipid-ligands was measured for 120s to 240s and dissociation of analyte molecules was measured for 240s to 600s. Following each analyte injection, the sensor chip surface was regenerated with two injections 50mM NaOH at 100 μl/min for 18s. Analysis was carried out with at least two separate protein purifications, each being analysed by multiple injections.
Histone tail interaction: An NLC (BioRad) sensor surface was conditioned by vertical injections of 1M NaCl, 50mM NaOH, 1M NaCl and 50mM NaOH at 30 μl/min for 60s. Six different types of biotinylated histone peptides were captured onto the NeutrAvidin surface at 30 μl/min for 90s to 200s at 25°C. The sensor surface was post-conditioned with two vertical, followed by two horizontal injections of 10mM HCl at 100 μl/min for 18s. Analyte injection were carried out as described for lipid interaction.

Fluorescence polarization: Experiments were performed in FP buffer (PBS; 10μM $ZnSO_4$) using a HYDEX Chameleon plate reader at 4°C as previously described (Fischle et al., 2008). The association of N-terminally fluorescein-labelled peptides H3 (aa 1-15) unmodified and K4me3 with purified recombinant WT, KK and DW-TAF3 GST-fusion protein was measured in the absence and presence of 5-fold molar excess of PI5P Di-C4. Fluorescence anisotropy (FA) was calculated from measured intensities in the parallel and orthogonal plane. Raw anisotropy data were analyzed implying a one site binding model using the equation $A = A_f + (A_b – A_f)([R]/(K_d – [R]))$ with A, Anisotropy; A_b, anisotropy of bound state; A_f, anisotropy of unbound state; K_d, dissociation constant; [R], protein concentration. Curves were fit by least square fitting. Anisotropy readings were converted to fraction bound using the equation $F_b = (A - A_b)/(A_b - A_f)$; with F_b, fraction bound. Readings from multiple independent (minimum: 3) measurements were averaged after normalisation and blotted. Data was analysed using Prism graph pad 6. Analyses are derived from two separate protein purifications.

CHIP analysis

TAF3 and RNAPII ChIP assays were performed either as described (Lauberth et al., 2007) or using the Diagenode high cell ChIP kit. Cells were reversibly crosslinked in 1% (final) formaldehyde (Sigma) for 10min at room temperature and quenched by adding 125 mM (final) glycine (Sigma). Isolated chromatin was fragmented to an average size of 200-600bp with a biorupter (Diagenode). Precleared chromatin was immunoprecipitated overnight at 4°C and immunocomplexes were

collected with Protein A or G agarose coupled with salmon sperm DNA for 1.5h at 4°C. The immunocomplexes were eluted, crosslinks were reversed at 65 ºC for 4hr, and DNA was purified using QIA-quick spin columns according to manufacturer's instructions. CHiP DNA was analysed using qPCR.

Reference List

Fischle,W., Franz,H., Jacobs,S.A., Allis,C.D., and Khorasanizadeh,S. (2008). Specificity of the chromodomain Y chromosome family of chromodomains for lysine-methylated ARK(S/T) motifs. J. Biol. Chem. *283*, 19626-19635.

Lauberth,S.M., Bilyeu,A.C., Firulli,B.A., Kroll,K.L., and Rauchman,M. (2007). A phosphomimetic mutation in the Sall1 repression motif disrupts recruitment of the nucleosome remodeling and deacetylase complex and repression of Gbx2. J. Biol. Chem. *282*, 34858-34868.
