# Supplementary material for: The Basal Transcription Complex Component TAF3 Transduces Changes in Nuclear Phosphoinositides into Transcriptional Output
Source: Mol Cell. 2015 May 7;58(3):453–67. doi: 10.1016/j.molcel.2015.03.009 (PMC4429956; doi:10.1016/j.molcel.2015.03.009)
Supplement: Document S2. Article plus Supplemental Information [file mmc2.pdf]

Molecular Cell

Article

# The Basal Transcription Complex Component TAF3 Transduces Changes in Nuclear Phosphoinositides into Transcriptional Output

## Graphical Abstract

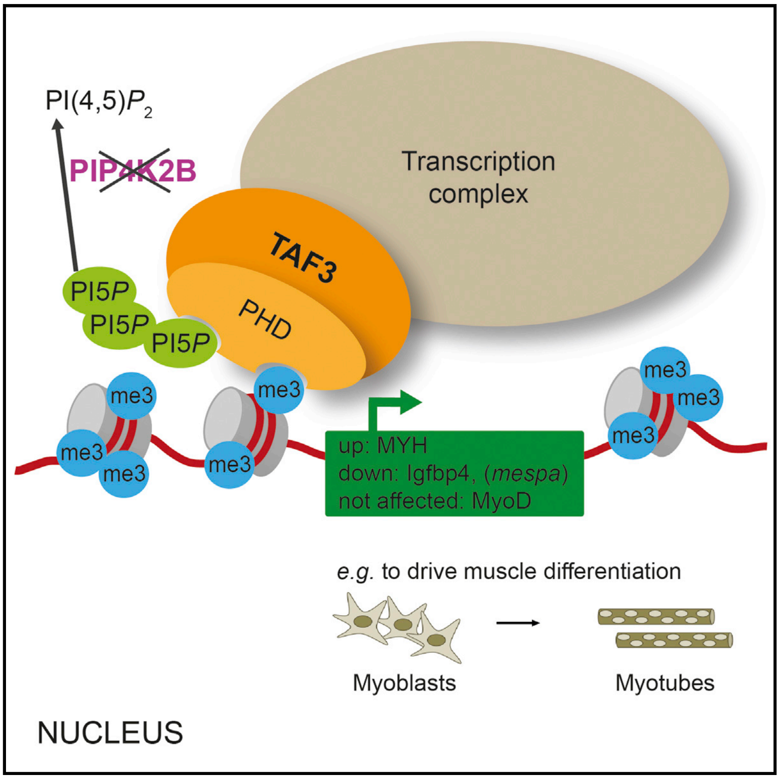


## Highlights

- PIP4K2B regulates nuclear PI5P and myogenic gene expression during differentiation
- A screen identifies 17 of 32 PHD fingers interacting with phosphoinositides
- The basal transcription component TAF3 interacts strongly with phosphoinositides
- TAF3 transduces changes in nuclear phosphoinositides into transcriptional output


## Authors

Yvette Stijf-Bultsma, Lilly Sommer, ..., Wolfgang Fischle, Nullin Divecha

## Correspondence

n.divecha@soton.ac.uk



## In Brief

Bultsma et al. show that the basal transcriptional complex protein TAF3 directly binds phosphoinositides and transduces changes in nuclear phosphoinositides into differential transcriptional output that affects myoblast differentiation. The lipid kinase PIP4K2B, phosphoinositides, and TAF3 form a conserved nuclear signaling pathway that selectively regulates transcription.


## Accession Numbers

GSE66353

Stijf-Bultsma et al., 2015, Molecular Cell *58*, 453–467
May 7, 2015 ©2015 The Authors
http://dx.doi.org/10.1016/j.molcel.2015.03.009

CellPress

# The Basal Transcription Complex Component TAF3 Transduces Changes in Nuclear Phosphoinositides into Transcriptional Output

Yvette Stijf-Bultsma,[1,2,8] Lilly Sommer,[2,8] Maria Tauber,[3] Mai Baalbaki,[4] Panagiota Giardoglou,[5] David R. Jones,[2] Kathy A. Gelato,[3] Jason van Pelt,[5] Zahid Shah,[1] Homa Rahnamoun,[6] Clara Toma,[6] Karen E. Anderson,[7] Philip Hawkins,[7] Shannon M. Lauberth,[6] Anna-Pavlina G. Haramis,[5] Daniel Hart,[4] Wolfgang Fischle,[3] and Nullin Divecha[1,2,*]
[1]The Inositide Laboratory, Centre for Biological Sciences, Highfield Campus, University of Southampton, Southampton SO171BJ, UK
[2]The Inositide Laboratory, the CRUK Manchester Institute, the University of Manchester, Wilmslow Road, Manchester M204BX, UK
[3]Laboratory of Chromatin Biochemistry, Max Planck Institute for Biophysical Chemistry, Am Fassberg 11, 37077 Göttingen, Germany
[4]University of California, San Francisco, Mail Code 3120, Smith Cardiovascular Research Building, 555 Mission Bay Boulevard, South San Francisco, CA 94158-9001, USA
[5]Institute of Biology (IBL), Leiden University, Sylvius Laboratory, Sylviusweg 72, 2333 BE Leiden, the Netherlands
[6]Division of Biological Sciences, Department of Molecular Biology, University of California, San Diego, La Jolla, CA 92093, USA
[7]Signaling Laboratory, The Babraham Institute, Cambridge, Cambridgeshire CB22 3AT, UK
[8]Co-first author
*Correspondence: n.divecha@soton.ac.uk
http://dx.doi.org/10.1016/j.molcel.2015.03.009
This is an open access article under the CC BY license (http://creativecommons.org/licenses/by/4.0/).

## SUMMARY

**Phosphoinositides (PI) are important signaling molecules in the nucleus that influence gene expression. However, if and how nuclear PI directly affects the transcriptional machinery is not known. We report that the lipid kinase PIP4K2B regulates nuclear PI5P and the expression of myogenic genes during myoblast differentiation. A targeted screen for PI interactors identified the PHD finger of TAF3, a TATA box binding protein-associated factor with important roles in transcription regulation, pluripotency, and differentiation. We show that the PI interaction site is distinct from the known H3K4me3 binding region of TAF3 and that PI binding modulates association of TAF3 with H3K4me3 in vitro and with chromatin in vivo. Analysis of TAF3 mutants indicates that TAF3 transduces PIP4K2B-mediated alterations in PI into changes in specific gene transcription. Our study reveals TAF3 as a direct target of nuclear PI and further illustrates the importance of basal transcription components as signal transducers.**

## INTRODUCTION

Pre-initiation complex (PIC) formation of eukaryotic type II RNA polymerase requires the general transcription factor TFIID. TFIID contains the TATA box binding protein (TBP) and TBP-associated factors (TAFs) and nucleates the PIC on core promoters by binding to the TATA box (Burley and Roeder, 1996; Thomas and Chiang, 2006). Recent studies have demonstrated that tissue-specific and selective gene transcription can be imposed by core promoter components (Hochheimer and Tjian, 2003). For instance, TAF3 regulates specific gene transcription as part of the large canonical TFIID PIC, and of a developmentally regulated smaller transcription complex with TBP and the TBP-like protein TRF3 (Deato and Tjian, 2007, 2008; Hart et al., 2009; Maston et al., 2012). These non-canonical complexes are involved in the regulation of transcriptional programs during differentiation (Hart et al., 2009; Deato and Tjian, 2007).

How TAF3 regulates gene expression is incompletely understood. Its plant homeodomain (PHD) finger mediates the interaction with histone H3 trimethylated at lysine 4 (H3K4me3) (Vermeulen et al., 2007), which is required to regulate selective gene expression in response to stress signaling (Vermeulen et al., 2007; Lauberth et al., 2013). However, chromatin association studies have shown that while TAF3 and TFIID complexes are constitutively associated with H3K4me3, knockdown of TAF3 does not strongly affect constitutive gene expression (Lauberth et al., 2013; Liu et al., 2011), suggesting that other induced regulators may influence TAF3's role as a transducer of cellular inputs to control differential transcriptional outputs.

Phosphoinositides (PI) are a family of seven signaling lipids regulating a wide variety of cellular processes including cell survival, proliferation, adhesion, and ion channel function (van den Bout and Divecha, 2009). PI are interconverted by the action of kinases and phosphatases, and degraded by phospholipases (Maffucci, 2012). The presence of different PI-modulating enzymes in subcellular compartments generates compartment-specific PI profiles that underpin organelle identity and function. PI and PI-modulating enzymes are present in the nuclear membrane, as well as within the nucleus (Shah et al., 2013; Divecha et al., 1991; Lindsay et al., 2006; Watt et al., 2004). In the latter, they are associated with interchromatin domains and affect nuclear functions. For example, the level of phosphatidylinositol 5-phosphate (PI5P) in chromatin is increased in response to stress

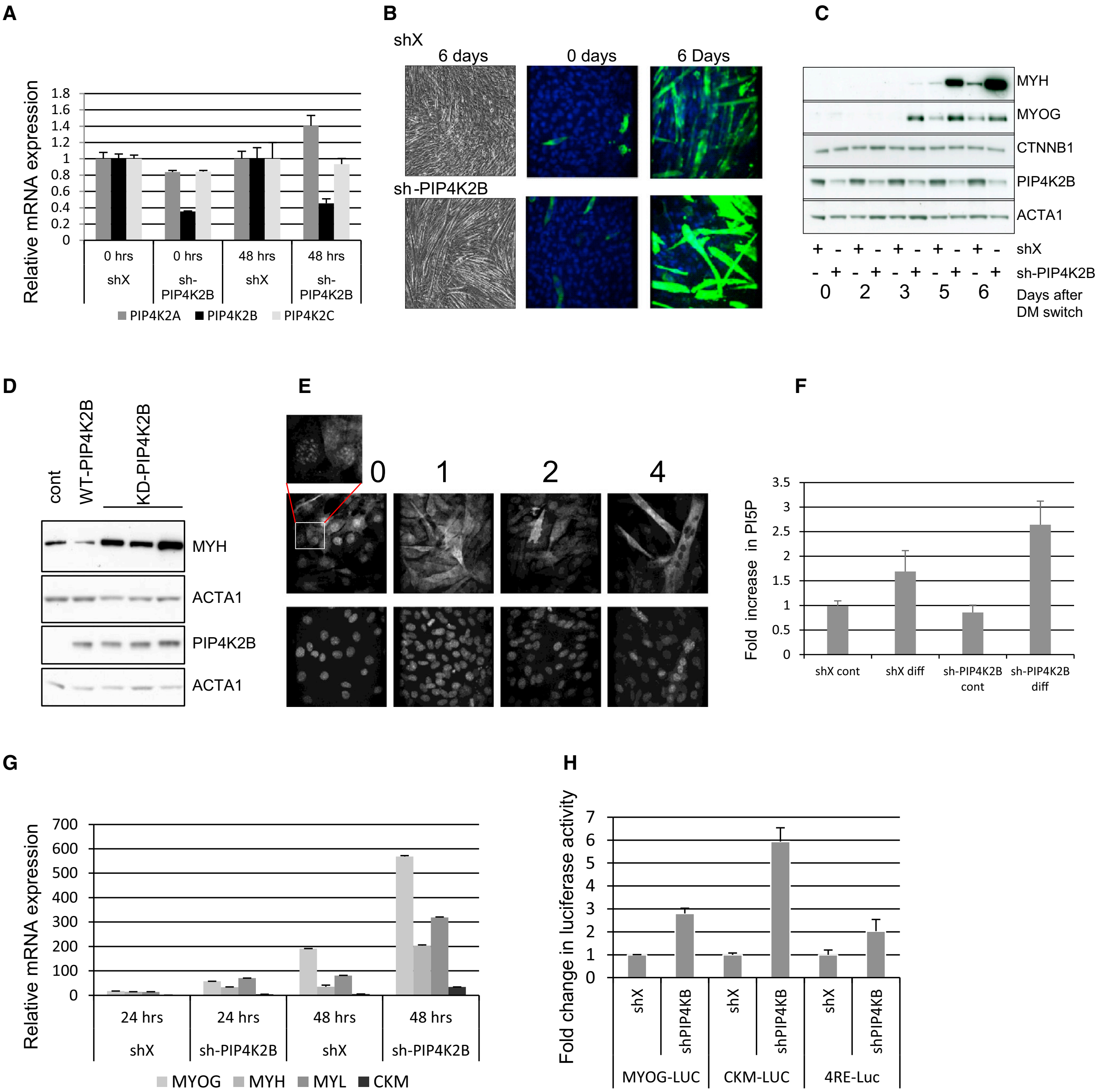


**Figure 1. PIP4K2B Regulates Myogenic Differentiation in C2C12 Myoblasts**

(A) Control (shX) and PIP4K2B knockdown (sh-PIP4K2B) C2C12 cells were differentiated for 0 hr or 48 hr and expression of PIP4K isoforms was determined by QRT-PCR.

(B) shX or sh-PIP4K2B C2C12 cells were differentiated for the indicated times, fixed, stained with an anti-MYH antibody (right four images), and myotube formation was depicted with by brightfield microscopy (left images).

(C) shX or sh-PIP4K2B C2C12 cells were differentiated for the times shown and the levels of proteins indicated were determined by immunoblotting.

(D) Control (Cont), wild-type PIP4K2B (WT-PIP4K2B), or kinase inactive PIP4K2B (KD-PIP4K2B) overexpressing C2C12 cells were differentiated for 4 days and lysed. Levels of proteins indicated were determined by immunoblotting.

(E) C2C12 cells overexpressing PIP4K2B were fixed at the indicated times (top). Top: localization of PIP4K2B as assessed by antibody staining. Bottom: nuclear staining (DAPI). The inset shows a higher magnification of the cells in the square.

(F) shX or sh-PIP4K2B C2C12 cells were differentiated for 2 days before isolation of nuclei by hypotonic lysis. PI5P levels were measured using a specific mass assay.

*(legend continued on next page)*

signaling (Jones et al., 2006) and affects p53 acetylation and gene transcription (Gozani et al., 2003).

PIP4K2A and 2B are isoforms within the PIP4K family of lipid kinases that phosphorylate and remove nuclear PI5P (Rameh et al., 1997; Bultsma et al., 2010; Wang et al., 2010; Clarke et al., 2007; Jones et al., 2006). PIP4K2A and 2B are deregulated in human cancers (Jude et al., 2014; Keune et al., 2013; Emerling et al., 2013), leading to expression changes of genes involved in cell-cycle progression, epithelial-mesenchymal transition (EMT), reactive oxygen accumulation and metabolism, ultimately affecting tumor growth. However, how PIP4K and PI5P directly influence specific gene transcription is yet unknown.

Nuclear PI regulate processes through their interaction with proteins containing PI-interacting domains (Lewis et al., 2011; Bidlingmaier and Liu, 2007; Bidlingmaier et al., 2011; Jungmichel et al., 2014) and their identification has linked nuclear PI to protein folding, DNA and chromatin regulation (Lewis et al., 2011), mRNA splicing and polyadenylation (Lewis et al., 2011; Mellman et al., 2008), and allosteric regulation of histone binding modules (Gelato et al., 2014). The PHD finger of Inhibitor of growth protein 2 (ING2) was one of the first convincingly shown to interact with nuclear PI, including PI5P (Gozani et al., 2003). PHD-fingers are present predominantly in nuclear proteins (Bienz, 2006) and are often mutated or deleted in human diseases (Musselman and Kutateladze, 2009). They are zinc-finger motifs facilitating protein/protein and protein/ligand interactions, for example by recognizing specific histone modifications (Pena et al., 2006a; Wysocka et al., 2006). The interaction of PHD fingers with both PI and histone tails suggests possible direct cross talk between a lipid signaling pathway and chromatin.

PIP4K2B is highly expressed in muscle tissue and, in this study, we show that it regulates the expression of muscle-specific genes as well as the extent of myoblast differentiation. We also show that the PHD finger is a common motif for PI binding, and that the basal transcription component TAF3 is a strong interactor of PI, particularly PI5P. Detailed studies revealed a pathway linking the PIP4K2B signal transduction pathway directly to the regulation of TAF3 and gene expression.

## RESULTS

### The Nuclear PI Kinase PIP4K2B Regulates Myoblast Differentiation

Because PIP4K2B is highly expressed in muscle tissue, we analyzed the role of PIP4K2B in myoblast differentiation using the C2C12 cell model. C2C12 myoblast cells can exit the cell cycle, synthesize muscle specific proteins, such as myogenin (MYOG) and myosin heavy chain (MYH), and finally fuse to form large multinucleate myotubes, resembling the differentiation of primary muscle myoblasts (Asp et al., 2011). We generated C2C12 cell lines stably depleted of PIP4K2B expression, without strongly affecting expression of the 2A or 2C PIP4K-isoforms (Figure 1A).

Although PIP4K2B knockdown did not significantly alter cell-cycle progression during differentiation (Figure S1A), it strongly increased myotube formation (Figure 1B) and significantly increased the myoblast fusion index (shX = 4.2 ± 1.2 [SD] and sh-PIP4K2B = 10.59 ± 3.0 [SD], $p < 0.000001$ Student's t test). PIP4K2B depletion increased the expression of late (MYH) (Figure 1B) and (early (MYOG) (Figure 1C) myogenic markers. Overexpression of wild-type kinase active PIP4K2B suppressed MYH expression whereas kinase-inactive mutants of PIP4K2B increased the expression of MYH in a similar manner to the knockdown of PIP4K2B (Figure 1D). These data show that PIP4K2B kinase activity is important in controlling the expression of muscle-specific proteins and the extent of C2C12 myoblasts differentiation.

PIP4K2B localizes in the nucleus in many cell types (Bultsma et al., 2010; Jones et al., 2006; Richardson et al., 2007; Boronenkov et al., 1998; Ciruela et al., 2000) and we thus assessed its localization in C2C12 cells undergoing differentiation. In 88% of undifferentiated C2C12 myoblasts, PIP4K2B was nuclear and showed defined punctate nuclear staining (Figure 1E and 0 days). After 1 day of differentiation, PIP4K2B staining became diffuse throughout the cell and after 4 days when myotubes were formed, PIP4K2B was excluded from the nuclei (Figure 1E, day 4). Loss of endogenous nuclear PIP4K2B was also observed by immunoblotting (Figure S1B). Correlating with decreased nuclear PIP4K2B during differentiation, we observed a 1.7-fold increase in nuclear PI5P levels. Depletion of PIP4K2B further increased nuclear PI5P levels (Figure 1F). Knockdown of PIP4K2B did not alter the nuclear levels of total PIP (predominantly PI4P) or $PIP_2$ (predominantly $PI(4,5)P_2$) as assessed by mass spectrometry. A small decrease in both phosphatidylserine and phosphatidylinositol was observed (Figure S1C).

Because PIP4K2B is nuclear in undifferentiated cells, we postulated that it might affect myoblast differentiation by regulating gene expression. Knockdown of PIP4K2B increased the expression of early and late myogenic marker genes including MYOG, MYH, myosin light chain (MYL), and muscle creatine kinase (CKM) (Figures 1G and S1D). Increased expression of myogenic markers was observed with three different sh-RNA constructs targeting PIP4K2B (Figure S1E) and all three increased myotube formation (Figure S1F). The increased expression of MYH and MYL could be partially rescued by overexpression of a PIP4K2B-PIP4K2A fusion protein (Figure S1G). Increased myogenic gene expression was not solely a consequence of increased numbers of cells undergoing differentiation as they were increased in isolated myotubes (Yoshida et al., 1998) depleted of PIP4K2B (Figure S1H). Increased myogenic transcription was implicated using luciferase expression driven by endogenous promoters of MYOG, CKM or from a synthetic MRF promoter (4RE) which revealed increased transcriptional activity upon PIP4K2B depletion (Figure 1H).

These data are consistent with a role for PIP4K2B in regulating nuclear PI5P levels that affect the expression of muscle-specific genes during myogenic differentiation.

(G) shX or sh-PIP4K2B C2C12 cells were differentiated for the times indicated (hours) and expression levels of indicated genes were determined with QRT-PCR.
(H) shX or sh-PIP4K2B C2C12 cells were transfected with luciferase constructs driven by the MYOG, CKM, or a synthetic MRF promoter (4RE) and differentiated for 48 hr. Luciferase activity was measured and normalized to firefly luciferase driven by a CMV promoter.
The values in (A), (F), (G), and (H) show fold changes compared to shX sample and represent the mean of triplicates +SD. See also Figure S1.

## PHD Finger Containing Proteins as Potential Downstream Targets of PIP4K2B

We reasoned that PHD finger containing proteins may be plausible downstream targets to couple changes in nuclear PI to gene expression (Gozani et al., 2003; Ndamukong et al., 2010). We cloned 32 different human PHD fingers and expressed them as GST fusion proteins in bacteria. Purified proteins (Figure 2A) were tested for interaction with PI using lipid dot blots (Figure 2B) and surface plasmon resonance (SPR) (Figures 2C and S2), together identified 17 PHD fingers as interactors of PI (Table S1). The specificity of PI interaction differed between the two lipid interaction assays, presumably reflecting differing presentation of the lipids.

PHD fingers also interact with methylated (Peña et al., 2006b; Shi et al., 2006) and non-methylated histone H3 tails (Rajakumara et al., 2011). Only three of the 32 PHD fingers preferentially interacted with a methylation modified H3 peptide (TAF3, ING3, and DIDO1), whereas many of the others preferred unmodified H3 peptide, which in some cases was reduced by methylation of the peptide at either K4 or K9 (Figure 2C and Table S1). For example, CXXC1 bound preferentially to the unmodified H3 peptide, while methylation of K9 but not K4 decreased its interaction (Figure 2C).

## The PHD Finger of the Basal Transcription Complex Component TAF3 Interacts with PI

Among the 17 PI interacting PHD fingers, we identified TAF3 (Figure 3A), a component of the basal transcription complex that interacts with H3K4me3 (Vermeulen et al., 2007) (Figure 3B) and is a regulator of C2C12 myoblast differentiation and muscle-specific gene transcription (Deato et al., 2008; Deato and Tjian, 2007).

We first determined how PI interacted with the PHD finger of TAF3. Mutation of conserved cysteine residues for zinc coordination ablated interaction with PI, demonstrating the requirement for an intact PHD finger (Figure S3A). Alignment of PI interacting PHD finger sequences suggested that a polybasic region (PBR) at the C terminus may be important for the interaction with PI (data not shown), which was confirmed by deletion of the PBR (Figures S3B–S3E). Combinatorial mutagenesis of lysine 922, 923, 925, 926 and 928, and arginine 927 completely ablated PI interaction of PHD-TAF3, while maintaining the specificity and interaction of the PHD finger with H3K4me3 peptide (Figures 3C and S3B–S3E). In contrast, mutagenesis of D890 and W891 residues disrupted the H3K4me3 interaction (Figure 3D, right) but did not significantly alter its interaction with PI (Figure 3D, left).

These data show that the PHD finger of TAF3 interacts independently with PI and histone methylation marks enabling the generation of PHD-TAF3 mutants that only maintain H3K4me3 (KK-TAF3) or PI interaction (DW-TAF3), which we used to further study the relationship between PI5P and TAF3 during myoblast differentiation.

## PI Interaction Regulates TAF3-Dependent Gene Expression during Myoblast Differentiation

Stable knockdown of TAF3 in C2C12 cells (Figures 4A and S4) did not alter their cell growth or the cessation of proliferation upon differentiation (Figure 4B), but severely attenuated C2C12 myoblast differentiation (Deato and Tjian, 2007) and expression of MYOG and MYH (Figure 4C). As expected, mRNA levels of specific myogenic markers (MYOG, CMK, MYL, and MYH) were reduced in TAF3 knockdown cells (Figures 4D and S4A).

TAF3-depleted cells were rescued using either murine wild-type (WT) TAF3 or mutants that only maintain H3K4me3 (KK-TAF3) or PI interaction (DW-TAF3), which were all similarly expressed (Figure 4E). These cell lines were differentiated and the expression of myogenic markers was determined. As expected, the expression of both MYH and MYOG was rescued by WT TAF3 (Figure 4F), whereas their expression was compromised by rescue with either KK-TAF3 or DW-TAF3 (Figures 4F and 4G). Late-stage myotube formation was also compromised in both the KK- and the DW-TAF3 mutant (Figure S4B).

These data show that PHD finger interactions with both PI and H3K4me3 are required for TAF3 to drive proper muscle-specific gene transcription during differentiation of C2C12 cells.

## PIP4K2B Regulates Specific Gene Expression through the PI Interacting Site of TAF3

We next explored the specific relationship between PIP4K2B expression and the TAF3-PI interaction site in the regulation of muscle differentiation. Specifically, we analyzed if myogenic genes that are regulated by PIP4K2B knockdown require the PI interaction site of TAF3. TAF3-depleted cells were rescued with either WT or KK-TAF3 and were additionally depleted of PIP4K2B (Figures 5A and S5A). QRT-PCR and immunoblotting showed that WT and KK-TAF3 (Figures S5A and S5B) were expressed to similar levels. After differentiation the expression levels of MYOG, MYH and MYL were rescued by the expression of WT-TAF3 and were further increased when PIP4K2B was depleted (Figure 5B, bars 3 and 4). KK-TAF3-expressing cells showed attenuated expression of myogenic markers that were not further induced by PIP4K2B depletion (Figures 5B, S5C, and S5D). In contrast, expression of the myogenic factor MYOD, which is dependent on TAF3 expression, was similarly expressed in both WT and KK-TAF3 rescued cells (Figures 5B, S5C, and S5D). These data directly link TAF3-PI interaction to the regulation of the expression of a subset of myogenic markers.

## PIP4K2B Positively and Negatively Regulates TAF3-Mediated Gene Expression

To gain further insight into the relationship between PIP4K2B and transcriptional regulation by TAF3, we carried out microarray gene expression analysis of the cell lines outlined in Figure 5A, which were either differentiated for 2 days or treated with etoposide for seven hours. Etoposide treatment increased nuclear PI5P in other cell types (Jones et al., 2006), allowing the study of TAF3-PI interaction in a setting unrelated to differentiation (accession number GSE66353). Principal component (PC) analysis (Figure S6A) of the top 500 most variable genes showed that the biological triplicate arrays for each genotype were highly coherent and that the variation within the data sets could cluster C2C12 cells based on their specific genotype. Gene set enrichment analysis (GSEA) (Subramanian et al., 2005) indicated that gene expression programmes upregulated either during myoblast differentiation (GSE19968) (van Oevelen et al., 2010) or by MYOD overexpression were highly enriched in cells with

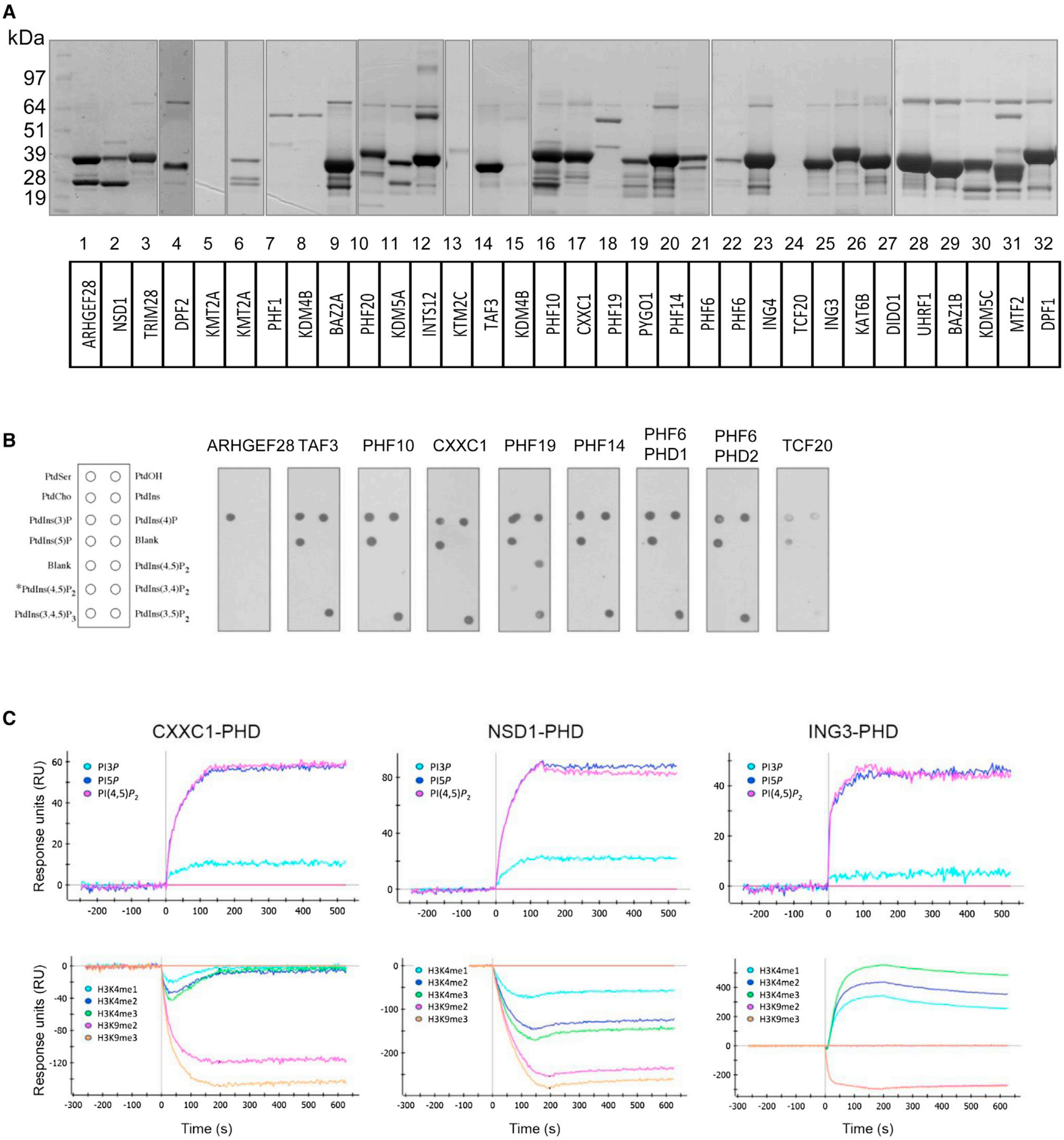


**Figure 2. A Small-Scale Screen Identifies PHD Fingers as PI Interactors**

(A) 32 PHD finger sequences were PCR-amplified, cloned into pGEX-4T1, and expressed and purified as GST-fusion proteins. Proteins were analyzed by SDS-PAGE and Coomassie blue staining. Numbers correspond to numbers in Table S1.

(B) Lipid dot blots (schematic left) were probed with GST-PHD fingers as indicated and interactions were visualized using an anti-GST antibody.

(C) GST-PHD fingers were analyzed for interaction with PI (top) and histone H3 peptides (bottom) by SPR. Positive controls are shown in Figure S2. The PI-PHD finger interaction was dependent on the presence of Zn in the analyte buffer, which could not be replaced by magnesium. ING3 strongly interacts with H3K4me3 peptide, whereas both CXXC1 and NSD1 interact best with unmodified H3 (as shown by negative SPR responses after referencing to H3-unmodified peptide). See also Figure S2 and Table S1.

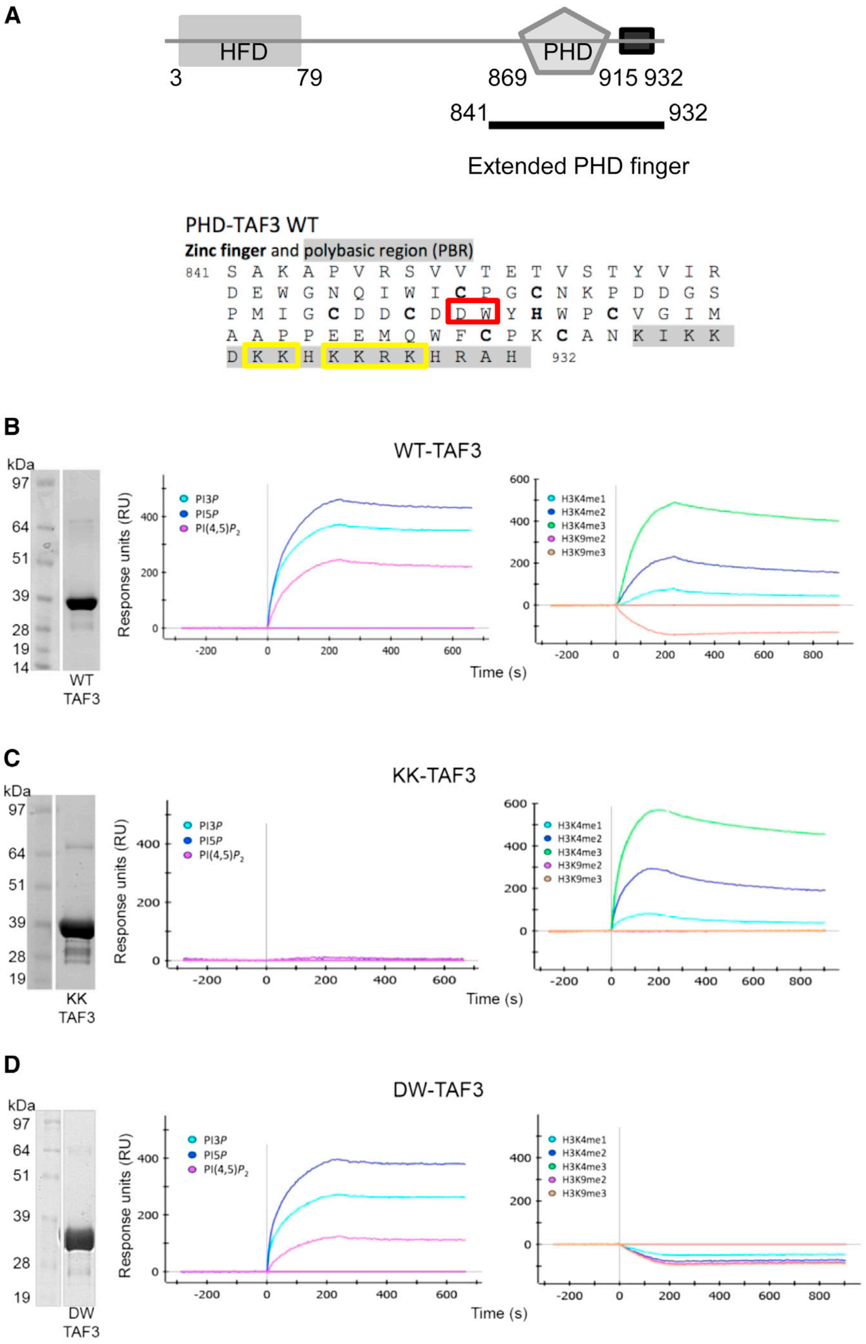


**Figure 3. The Extended PHD Finger of TAF3 Mediates Its Interaction with PI**

(A) Cartoon depicting the structure of TAF3 including the extended PHD. Amino acids in bold are required for the interaction with zinc, those boxed in red are required for the interaction with H3K4me3 and mutated in the DW-TAF3 mutant, and those boxed in yellow are mutated in the KK-TAF3 mutant to attenuate interaction with PI.

(B–D) Interaction of WT (B), KK mutant (C), and DW mutant (D) TAF3 PHD finger with PI (left) and modified histone peptides (right) assessed with SPR.

See also Figure S3.

PIP4K2B depletion and in cells expressing WT-TAF3 compared to KK-TAF3 (Figure S6B). However, depletion of PIP4K2B or expression of KK-TAF3 did not affect all aspects of differentiation because genes that are normally downregulated during differentiation (van Oevelen et al., 2010) were not enriched (data not shown).

Comparative expression analysis showed that 550 genes changed upon depletion of PIP4K2B ($p < 0.05$, >1.4-fold difference), of which 331 were upregulated. 202 genes were differentially expressed between WT and KK-TAF3 rescued C2C12 cells ($p < 0.05$, 1.4-fold cut off) (Figure S6C). Sixty-three genes were deregulated in both conditions (Figure S6B) and analysis of changes in the overlapping genes showed that those that are increased by PIP4K2B depletion are decreased in KK-expressing cells. Conversely, genes downregulated when PIP4K2B is depleted are more highly expressed in KK-TAF3 rescue cells (Figure 6B). GSEA also demonstrated that genes upregulated upon PIP4K2B knockdown (increase in nuclear PI5P) were highly enriched in WT-TAF3 expressing cells, whereas those downregulated in PIP4K2B knockdown cells were highly enriched in KK-TAF3 cells. These data suggest that PIP4K2B regulates gene transcriptional output by changing the levels of nuclear PI, which modulates TAF3 function by directly interacting with the PBR domain of TAF3.

To verify the microarray analysis we analyzed gene expression by QRT-PCR. Another six genes (CKM, Sprr2b, Tmem8C, Acta1,Trdn, and C1qTNF3) showed similar patterns of expression as MYOG, MYH, and MYL (Figures 5B and 6D). Their upregulation when PIP4K2B was depleted was suppressed in KK-TAF3 rescue cells (Figure 6D). We also identified another group of genes (Ncam2, Igfbp4, Dkk3, Ces2g, and Prelp) whose expression was partially decreased in PIP4K2B knockdown cells and strongly decreased in KK-TAF3 cells (Figure 6D).

In etoposide-treated cells, 236 genes were differentially expressed in PIP4K2B-depleted cells, whereas 127 genes were differentially expressed between WT-TAF3 and KK-TAF3 rescue cells, with a highly significant overlap of 30 genes (Figure S6D). These 30 genes again highlighted a co-regulated response. However, most of the genes in the overlap were downregulated in PIP4K2B depleted cells and correspondingly upregulated in KK-TAF3 rescue cells (Figure 6E). GSEA of the genes

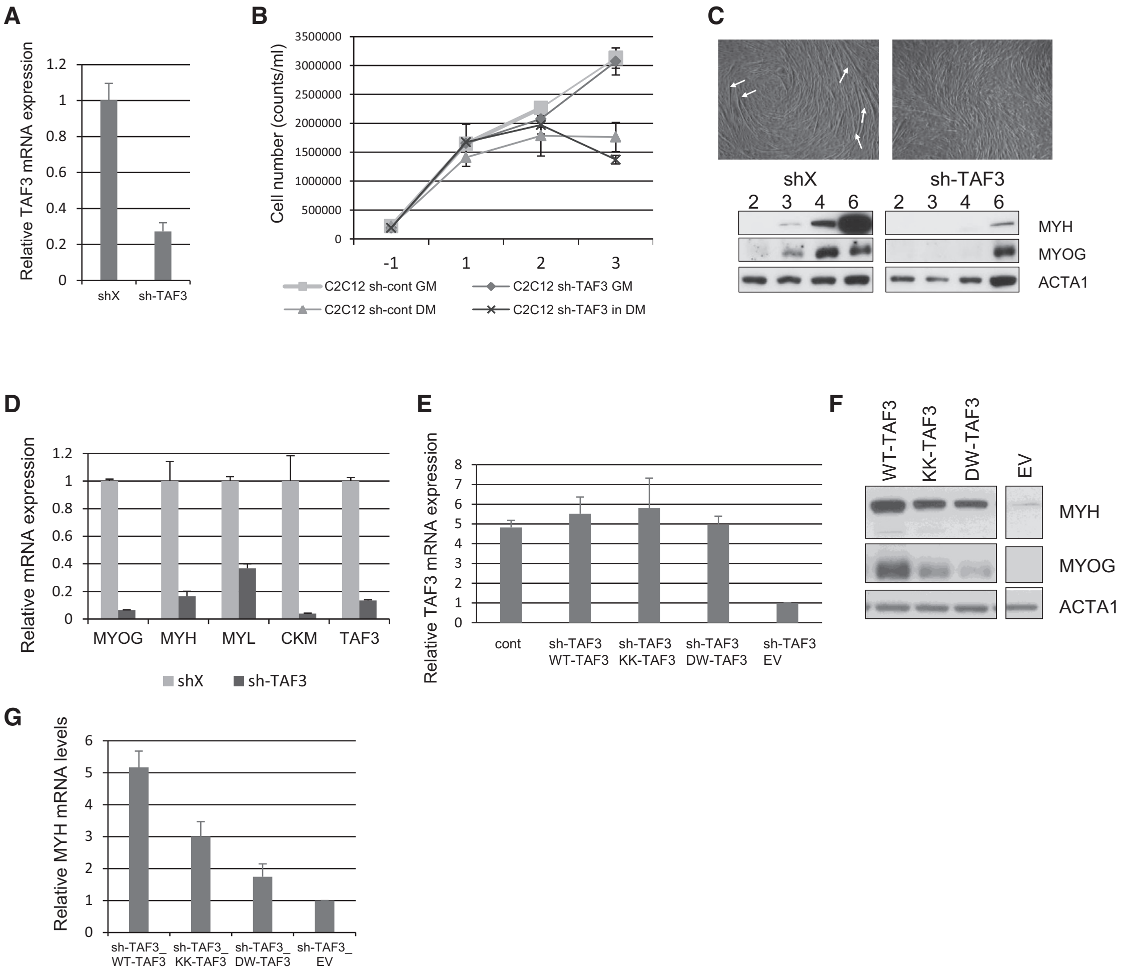


**Figure 4. TAF3-Mediated Gene Expression during Differentiation Requires the PI and H3K4me3 Interaction Sites**

(A) A combination of two sh-constructs targeting the 3′UTR of mouse TAF3 were used to generate C2C12 cells with stable TAF3 knockdown, and assessed for TAF3 expression compared to shX with QRT-PCR.

(B) Proliferation of shX or sh-TAF3 C2C12 cells in growth (GM) or differentiation medium (DM). The graph shows cell numbers and days of differentiation, and data represent the mean of triplicates + SD.

(C) shX or sh-TAF3 C2C12 cells were differentiated for 6 days and myotubes were depicted with brightfield microscopy. Cell lysates from cells differentiated for the days indicated were analyzed for protein expression by immunoblotting.

(D) shX or sh-TAF3 C2C12 cells were differentiated for 4 days and gene expression changes determined by QRT-PCR as indicated.

(E) TAF3-depleted C2C12 cells (sh-TAF3) were reconstituted with wild-type TAF3 (WT-TAF3), or mutants that only maintain H3K4me3 (KK-TAF3) or PI interaction (DW-TAF3) respectively, or with empty vector (EV). Control cells were not depleted of TAF3 (cont). TAF3 expression was determined by QRT-PCR.

(F) TAF3-depleted C2C12 cells were reconstituted with the indicated constructs and were differentiated for 4 days and levels of proteins indicated were determined by immunoblotting.

(G) TAF3-depleted C2C12 cells (sh-TAF3) were reconstituted as indicated and were differentiated for 4 days and MYH expression was determined by QRT-PCR.

The values in (A), (D), (E), and (G) show fold changes compared to control sample (shX for A and D; shTAF3-EV for E and G) and represent the mean of triplicates +SD. See also Figure S4.

downregulated by PIP4K2B knockdown (increase in PI5P) showed that they were more highly expressed in KK-TAF3 compared to WT-TAF3 rescue cells (normalized enrichment score −1.408, FWER p = 0.05 with 72 of 151 genes enriched) (Figure 6F). However, there was no enrichment of genes upregulated by PIP4K2B knockdown (data not shown). QRT-PCR showed a decrease in expression of Tspan7, Pdgf, IL33 v.2, and Decorin (Dcn) in response to PIP4K2B depletion in WT-TAF3 rescue cells, which was suppressed in KK-TAF3 rescue cells. EpH5 and Sprr2b expression increased upon PIP4K2B knockdown, which was suppressed in KK-TAF3 rescue cells. Expression of p21 was not effected by any of the conditions but as expected was upregulated in response to etoposide (Figure 6G).

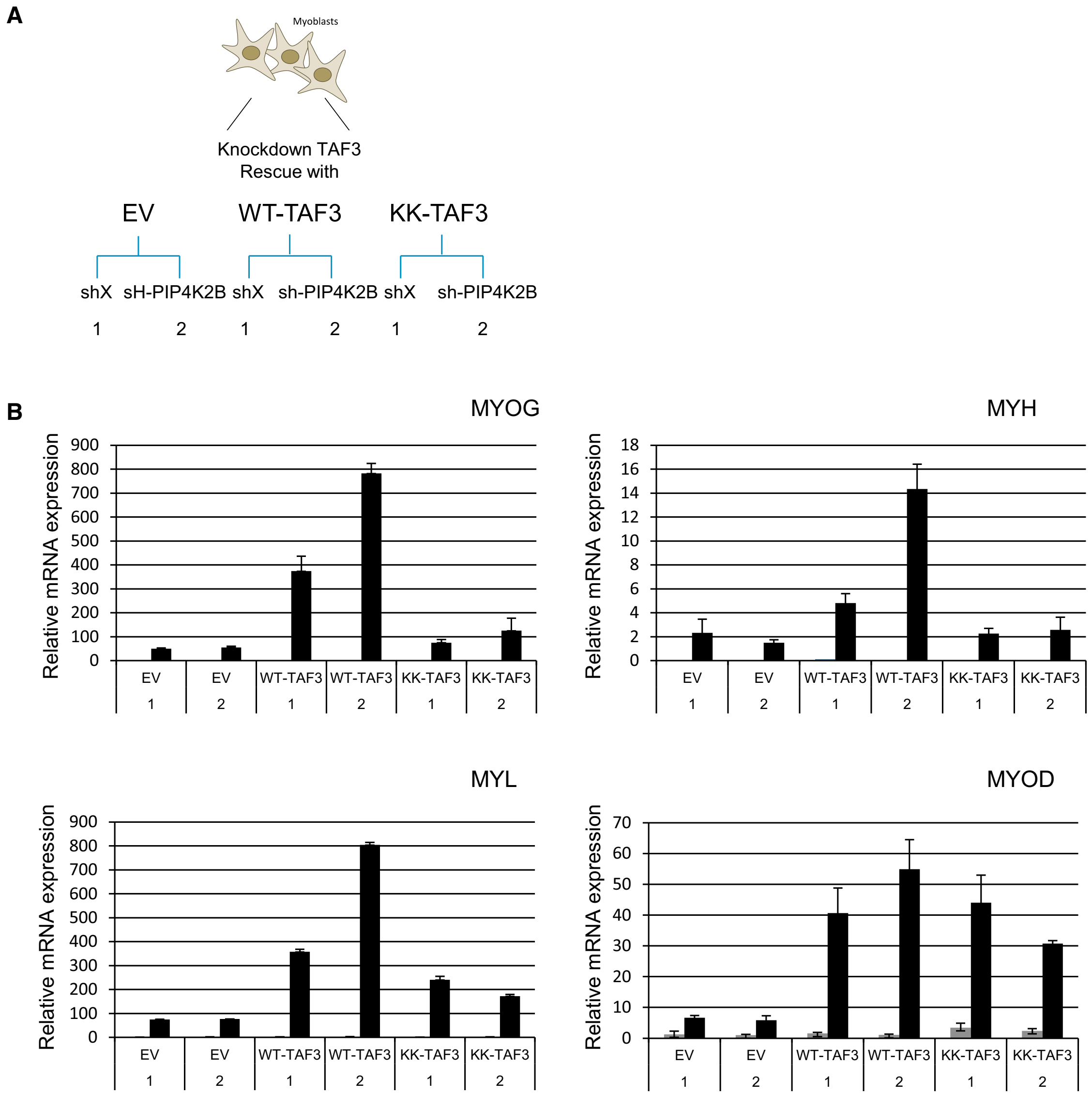


**Figure 5. PIP4K2B Increases Gene Expression during Differentiation through the PI Interaction Site of TAF3**

(A) Scheme depicting the cell types generated and used to study the relationship between PIP4K2B and TAF3.

(B) C2C12 cells depicted in Figure 5A were differentiated and gene expression determined by QRT-PCR as indicated. Expression of early myogenic markers (MYOG, MYOD) was assessed after 2 days of differentiation, whereas late myogenic marker expression (MYH, CKM) was assessed at 4 days. The full time course is shown in Figure S5B. 1 indicates control knockdown (shX) and 2 indicates knockdown of PIP4K2B (sh-PIP4K2B) in the respective TAF3 rescue cell lines. The data represent fold changes compared to the 0 hr shX sample (not shown) and represent the mean of triplicates + SD.

See also Figure S5.

The simplest interpretation of these data is that PIP4K2B-mediated changes in nuclear PI can stimulate and repress the expression of specific genes through the TAF3-PI interaction site.

## PI Interaction Modulates TAF3 PHD Finger Interaction with H3K4me3

How might TAF3-PI interaction mediate transcriptional regulation? TAF3 regulates gene expression through its interaction with H3K4me3 (Vermeulen et al., 2007), the myogenic transcription factor MyoD (Deato et al., 2008) and the TFIID complex. However, interaction with these transcriptional coponents was not altered in the KK-TAF3 mutant. Full-length WT-TAF3 and KK-TAF3 showed similar interactions with H3K4me3 peptides, while DW-TAF3 as expected was compromised in this interaction (Figure S7A). WT-TAF3, KK-TAF3, and DW-TAF3 similarly interacted with TAF10 (Figure S7B), which mediates the association of TAF3 with the canonical TFIID complex. Finally, both WT-TAF3 and KK-TAF3 interacted similarly with MyoD (Figure S7C) as assessed with co-immunoprecipitation (coIP).

Using fluorescence polarization, we next tested if PI modulates the conformation of the PHD finger. Measurements with labeled H3K4me3 peptide showed that WT-TAF3 PHD finger interacted with H3K4me3 (KD $\approx 1\ \mu M$, Figure 7A) but as expected not with non-methylated peptide (data not shown). Addition of a 5-fold molar excess of PI5P increased the KD to 3.5 $\mu M$ (Figure 7A). In contrast, the KK-TAF3 PHD finger exhibited a KD of 0.5 $\mu M$ and showed no change in KD upon addition of PI5P (Figure 7B). Interestingly, PI(4,5)$P_2$ which also interacted with TAF3 by SPR, also modulates the interaction of the PHD finger with H3K4me3 (Figure S7D). These data suggest that PI interaction can modulate the interaction of TAF3 with H3K4me3.

To determine if this occurs in vivo, we assessed the promoter occupancy of endogenous TAF3 using chromatin immunoprecipitation (ChIP). Upon differentiation, TAF3 was highly enriched at PIP4K2B regulated genes (MYOG and Sprr2b) and other genes (MYOD and GAPDH), and knockdown of TAF3 validated antibody specificity (Figure 7C). Differentiation modestly increased both RNA polymerase II (RNAPII) and TAF3 occupancy at the MYOG promoter and gene body as well as at the MyoD promoter. Occupancy of RNAPII and TAF3 after differentiation was not altered by PIP4K2B knockdown (Figure 7D). There was a modest significant increase in TAF3 occupancy at the MyoD and GAPDH promoter in undifferentiated conditions suggesting that nuclear PI might stimulate TAF3 binding in the absence of differentiation (Figure 7D). TAF3 and RNAPII occupancy was also determined at later points of differentiation at both the MYH (Figure 7E) and MYOG promoter (Figure S7E). RNAPII and TAF3 occupancy increased during differentiation and there was a modest increase in TAF3 binding in PIP4K2B depleted undifferentiated cells. Strikingly, 72 hr after differentiation, TAF3 occupancy was strongly decreased in PIP4K2B-depleted cells at both the MYH and the MYOG promoter, suggesting that PI negatively regulates chromatin interaction of TAF3 in vivo. To determine if PI interaction alone could regulate TAF3 in vivo, we assessed the occupancy of KK-TAF3 compared to WT-TAF3. KK-TAF3 occupancy was significantly increased at all genomic regions (Figure 7F), directly linking TAF3 chromatin occupancy with PI interaction.

### PIP4K Regulates TAF3-Mediated Gene Expression in Zebrafish

To determine whether a PIP4K/PI/TAF3 pathway is evolutionarily conserved, we analyzed its function in Mespa expression and on muscle morphology in zebrafish (*Danio rerio*). Mespa is an essential transcription factor in primitive hematopoiesis initiation and is a direct target of TAF3. TAF3 binds the promoter of the *mespa* gene and TAF3 depletion reduces *mespa* expression and attenuates hematopoiesis (Hart et al., 2009). Alignment of the sequence of *taf3* from different organisms showed that residues within the PBR region that are crucial for PI interaction are highly conserved (Figure S7F). Depletion of PIP4K in zebrafish embryos (Elouarrat et al., 2013) led to a developmental phenotype that was suppressed by the co-injection of mRNA coding for a kinase active PIP4K but not a kinase inactive enzyme (Figure S7G), suggesting that regulation of PI underlies in part the phenotypic defect of PIP4K depletion. At 1 day post fertilization (dpf), depletion of PIP4K did not induce gross morphological abnormalities (Figure 7G) nor did they affect TAF3 expression levels. PIP4K depletion however, significantly decreased the expression of *mespa*. The decrease in *mespa* was rescued by the co-expression of kinase active PIP4K but not by the inactive PIP4K, strongly suggesting that *mespa* expression is regulated by changes in PI (Figure 7G). The expression of early hematopoiesis markers (*scl* and *lmo2*) was also decreased in PIP4K-depleted zebrafish (Figure S7H) in line with decreased Mespa function.

Although TAF3 has been implicated in C2C12 myoblast differentiation, its role in muscle development in zebrafish is not known. We therefore depleted zebrafish TAF3 and assessed myosin filament architecture by immunostaining. TAF3 knockdown led to a significant disruption of myofibril alignment, somite boundaries, and shape as monitored by MYH staining. The phenotype could be rescued by co-injection of human WT-TAF3 (Figure 7H) but not by the mutants that only maintain H3K4me3 (KK-TAF3) or PI interaction (DW-TAF3). In fact, expression of the KK-TAF3 appeared to enhance the disorganization of myosin filaments. A similar disorganization of myofibril alignment was observed upon depletion of PIP4K in zebrafish (Figure S7I). Furthermore, mutation of *mespa* leads to muscle phenotypes that resemble those for TAF3-depleted embryos (D.H., unpublished data). These data support a role for a PIP4K/TAF3/mespa pathway in the regulation of zebrafish muscle development and strongly implicate nuclear PI in transcriptional regulation during development in vivo.

## DISCUSSION

Levels of nuclear PI change in response to cell-cycle progression (Clarke et al., 2001), differentiation (Divecha et al., 1995), and in response to stress signaling (Jones et al., 2006) in both animals and plants (Meijer et al., 2001; Ndamukong et al., 2010) and are transduced into functional outputs by their interaction with proteins. Their interactors have implicated roles for nuclear PI in regulating histone modification (Ndamukong et al., 2010), chromatin binding (Gozani et al., 2003; Jones et al., 2006; Gelato et al., 2014), mRNA polyadenylation (Mellman et al., 2008), and topoisomerase activity (Lewis et al., 2011). Here, we show that PIP4K2B modulates C2C12 differentiation and muscle-specific gene expression. Detailed analysis revealed a pathway that directly links PIP4K2B and nuclear PI to the regulation of TAF3, a component of the basal transcription complex.

The effect of PIP4K2B knockdown on C2C12 myoblast differentiation is notable because PIP4K2B is highly expressed in muscle tissue. C2C12 cells resemble activated satellite like cells that are poised to differentiate to repair muscle after injury. This might implicate the PIP4K2B/TAF3 pathway in regulating satellite cell function and muscle biology in vivo. In zebrafish, we show that compromising the interaction of TAF3 with either H3K4me3 or PI and depleting PIP4K affects muscle fiber alignment in vivo. We suggest that this may occur through the regulation of *mespa* because both TAF3 and PIP4K regulate its expression and mutating *mespa* confers a similar phenotype (D.H., unpublished data).

Previous studies in human cells demonstrated that while 43% (11,000 genes) of protein-coding genes are bound by TAF3, the

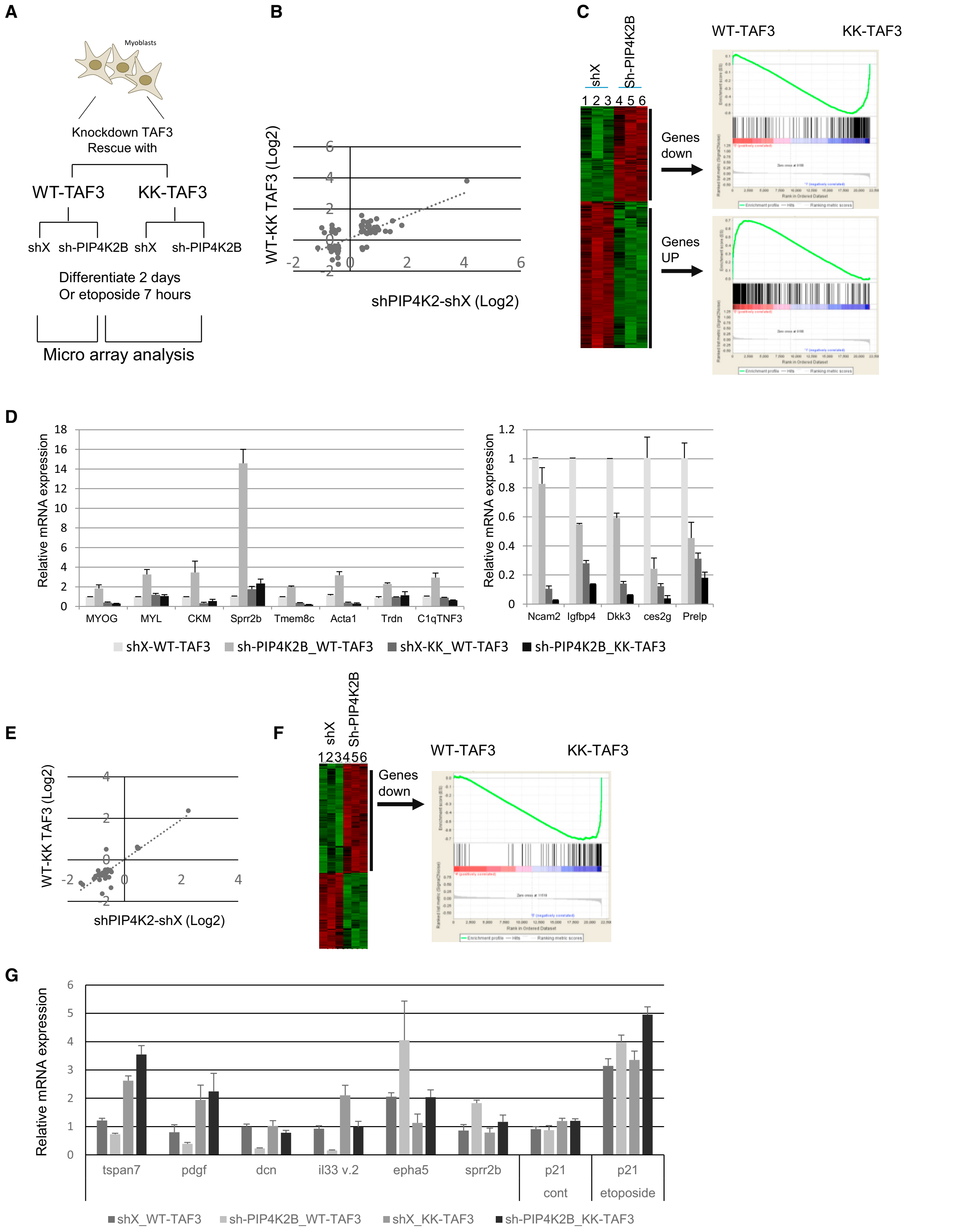


*(legend on next page)*

transcription of only 119 genes is affected by TAF3 knockdown, whereas after doxorubicin treatment thousands of genes are affected (Lauberth et al., 2013). A similar effect was observed during differentiation (Liu et al., 2011). This strongly suggests that signal-induced regulation of TAF3 is essential for TAF3 to modulate gene transcription. While changes in H3K4me3 are clearly important for TAF3 signaling, we propose that changes in nuclear PI are another such relevant signal. Our studies in C2C12 cells reveal at least four different groups of genes that are regulated by the PI interaction site of TAF3: group 1 increase on PIP4K2B depletion and is suppressed by loss of the TAF3 PI interaction site (e.g., MYOG); group 2 is downregulated by PIP4K2B depletion and increased by loss of the PI interaction site (e.g., Tspan7); group 3 is partly decreased by PIP4K2B depletion and more strongly suppressed by loss of the PI interaction site (e.g., Igfbp4), and group 4 is not regulated by PIP4K2B depletion but is regulated by PI interaction. A simple hypothesis that might give account for these groups is that 1 and 2 are regulated by PI5P, the substrate of PIP4K2B, whereas group 3 is positively regulated by PI(4,5)$P_2$, the product of PIP4K2B activity. Because the pool of PI(4,5)$P_2$ regulated by PIP4K2B is likely to be small compared to total nuclear PI(4,5)$P_2$ this would likely require additional mechanisms to target TAF3 to this small pool such as its interaction with PIP4K2B. Group 4 is likely to be regulated by other nuclear PI that are not influenced by PIP4K2B depletion such as PI4P. Groups 1 and 2 suggest that nuclear PI can both positively and negatively affect TAF3-mediated gene expression through a single interaction site.

PI modulates the interaction of TAF3 PHD finger with H3K4me3 in vitro and in vivo. However, chromatin occupancy cannot be the sole driver of PI-regulated gene expression because KK-TAF3 has increased occupancy at promoters but actually shows decreased transcriptional output. The interaction of TAF3 with H3K4me3 is also affected by other histone signals such as H3R2 methylation, H3K9 acetylation (Vermeulen et al., 2007), and H3T3 phosphorylation (Varier et al., 2010), and PI interaction may differentially affect how TAF3 interacts with these combinatorial modifications. The observed switch between predominantly negative regulation of TAF3-mediated transcription (during etoposide treatment) to both positive and negative regulation during myoblast differentiation might implicate a role for PI in regulating different TAF3 complexes. Myoblast differentiation switches TAF3-mediated transcription from the canonical TFIID complex to a simpler TAF3/TRF3 complex (Deato and Tjian, 2007). TAF3 also regulates transcription through modulating chromatin looping. Thus, positive and negative effects of nuclear PI on gene transcription might reflect their differential influence on different TAF3 complexes. Furthermore, we suggest that nuclear PI exist as proteolipid signaling platforms (Shah et al., 2013; Blind et al., 2012) that function as organizing centers to recruit chromatin regulators and other enzymes that coordinate pathway specific gene transcription. Therefore, TAF3-mediated changes in transcriptional output in response to changes in PI may depend on the context of other PI interacting chromatin regulators. Nuclear PI platforms may also explain why not all TAF3-dependent genes are influenced by nuclear PI. For example, MyoD is strongly regulated by TAF3 but its expression levels are not influenced by either PIP4K2B depletion or by loss of the PI interaction site on TAF3. This group of genes may not be localized within a nuclear PI platform and thus might not be influenced by TAF3-PI interaction.

PIP4K2B depletion deregulated more genes than the loss of the PI interaction site of TAF3, suggesting that nuclear PI can also regulate gene expression independently of TAF3. 17 of 32 PHD fingers showed PI interaction, implicating nuclear PI in regulation and interpretation of histone modifications (e.g., PHF6, NSD1, MYST4, BAZ1B, TAF3, CXXC1, ING3, ING4, and UHRF1). Furthermore, PI-PHD finger interaction may explain the requirement for developmental switches in PHD finger proteins (Lessard et al., 2007) as well as the role of mutations in cancers. For example, PHF6 is mutated in the human disease Börjeson–Forssman–Lehman syndrome (Lower et al., 2002) and in human leukemia (Van Vlierberghe et al., 2010). In both conditions, nonsense mutations downstream of the second PHD finger have been found that are unlikely to disrupt the PHD finger structure but would delete a PBR. We predict that these

**Figure 6. PIP4K2B and TAF3 Coordinate Both Positive and Negative Regulation of Gene Expression during Differentiation and Etoposide Treatment**

(A) Scheme depicting cells used for microarray gene expression analysis. Comparisons were aimed at identifying genes regulated by PIP4K2B knockdown and PI-TAF3 interaction during differentiation or etoposide treatment of C2C12 cells.

(B) Correlation plot of changes in the expression of the 63 overlapping genes (Figure S6C) regulated by PIP4K2B depletion and expression of KK-TAF3 after differentiation for 2 days.

(C) Left: heatmap showing expression of genes in shX (1, 2, and 3) and sh-PIP4K2B (4, 5, and 6) knockdown C2C12 cells differentiated for 2 days. Defined gene sets that were up- or downregulated were used for GSEA to probe a ranked list of gene expression changes between WT-TAF3- and KK-TAF3-expressing cells. GSEA demonstrated that genes upregulated upon PIP4K2B knockdown (increased nuclear PI5P) are highly enriched in WT-TAF3 (interacts with PI5P) expressing cells (normalized enrichment score 3.22 and FWER $p = 0.0$ with 156 of 335 enriched) whereas downregulated genes are highly enriched in KK-TAF3 (does not interact with PI5P) (normalized enrichment score $-3.21$ FWER $p = 0.0$ with 112 of 215 genes enriched).

(D) Gene expression was assessed by QRT-PCR as indicated in C2C12 cells depicted in Figure 6A after differentiation for 2 days.

(E) Correlation plot of changes in the expression of the 30 overlapping genes (Figure S6D) regulated by PIP4K2B depletion and expression of KK-TAF3 after etoposide treatment.

(F) Left: heatmap showing expression of genes in triplicate shX (1, 2, and 3) and sh-PIP4K2B (4, 5, and 6) C2C12 cells after etoposide treatment. A gene set that was decreased upon PIP4K2B depletion was used for GSEA to probe a ranked list of gene expression changes between WT-TAF3- and KK-TAF3-expressing cells. Genes downregulated by PIP4K2B depletion (increased nuclear PI5P) are more highly expressed in cells expressing KK-TAF3 compared to WT-TAF3.

(G) Gene expression was assessed by QRT-PCR as indicated in C2C12 cells depicted in Figure 6A after etoposide treatment for 7 hr.

The values in (D) and (G) represent fold changes compared to the shX-WT-TAF3 sample and represent the mean of triplicates +SD. See also Figure S6.

CellPress

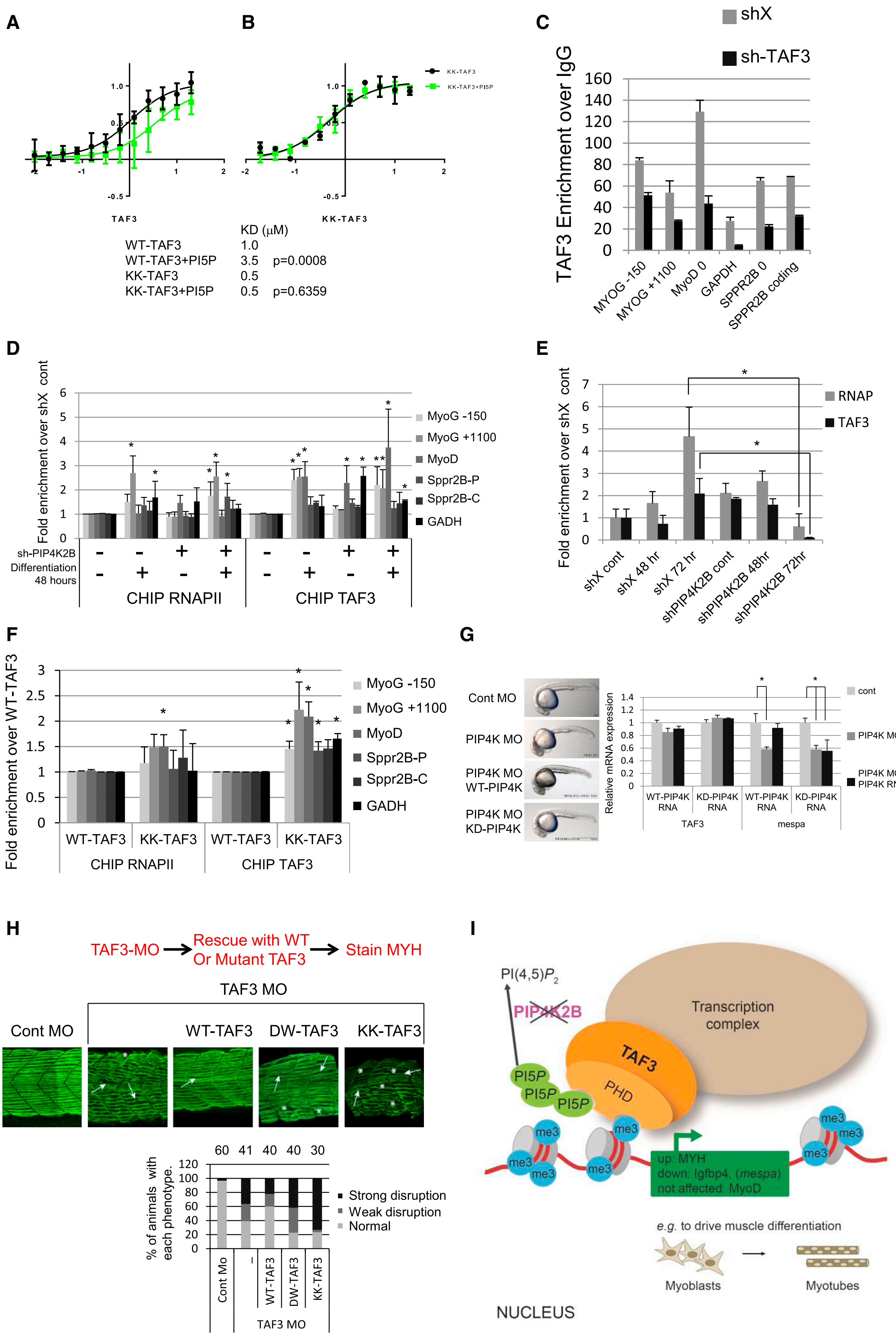


(legend on next page)

mutations would also disrupt interaction with phosphoinositides observed in this study.

In conclusion, this study reveals a pathway linking the regulation of nuclear PI directly to a core promoter component of the transcriptional machinery and further highlights how TAF3 can act to transduce signaling inputs to modulate transcriptional output.

## EXPERIMENTAL PROCEDURES

### C2C12 Maintenance and Differentiation

C2C12 cells were routinely cultured in F12-HAMS medium in 10% fetal calf serum. RNAi knockdown was accomplished using either retroviral (pRetrosuper) or lentiviral transduction (PLKO1 or 2). Cells overexpressing kinase active or inactive PIP4K2B were generated by retroviral transduction (pBabe); 300,000 cells were plated in six-well plates, and the next day differentiation was initiated by switching the cells into F12-HAMS medium containing 2% horse serum. Medium was replaced every 2 days. Cells were washed free of medium and either used in immunofluorescence studies, lysed in SDS-loading buffer for analysis by western blotting or RNA was isolated using RNAeasy and used for QRT-PCR and microarray analysis (accession number GSE66353).

### Phosphoinositide Analysis

Nuclei were isolated from control and differentiated cells and PI5P was analyzed as previously described (Jones et al., 2013) and its levels were normalized to the total amount of nuclear phospholipid phosphate. Other PIs were measured using mass spectrometry (Clark et al., 2011).

PHD fingers were identified using the SMART module and fragments were amplified with Phusion polymerase using specific primers (available on request) and cloned in frame with GST (pGEX-4T1). Proteins were purified and assessed for lipid interaction and for interaction with histone tails. Purified WT and mutant TAF3 PHD finger was also analyzed for interaction with H3K4me3 in the presence and absence of different PI using fluorescence polarization (Gelato et al., 2014).

### ChIP Analysis

TAF3 and RNAPII ChIP assays were performed either as described elsewhere (Lauberth et al., 2007) or using the Diagenode high cell ChIP kit. Immunocomplexes were eluted, crosslinks reversed, and DNA was purified using DNA spin columns. qPCR was performed to measure relative amounts of ChIP DNA (primer details available on request).

### Zebrafish Studies

Zebrafish were handled in compliance with local animal welfare regulations and were maintained according to standard protocols (http://zfin.org). The culture was approved by the local animal welfare committee of the University of Leiden and all protocols adhered to the international guidelines specified by the EU Animal Protection Directive 2010/63/EU or using standard methods at the UCSF CVRI zebrafish facility conducted in conformity with UCSF IACUC and AAALAC guidelines.

The use of the morpholino (3.5 ng MO2) and characterization of the phenotypes associated with knockdown of PIP4K has been previously described (Elouarrat et al., 2013). RNA was isolated using RNA easy kit (QIAGEN) and analyzed by qRT-PCR using suitable primers (available on request). Morpholinos designed to inhibit translation of zebrafish TAF3 and PIP4K have been previously described (Elouarrat et al., 2013; Hart et al., 2009). Morpholinos were microinjected into one cell stage WT zebrafish embryos, 3.5 ng per embryo of PIP4K MO, and 2.5 ng per embryo of TAF3 MO. One nanoliter of 50 ng/ul working solutions of DNA constructs were injected per embryo in rescue experiments.

Immunohistochemistry was performed in whole embryos according to standard protocols. Antibodies used were mouse anti-MyHC F59 (1:30, DSHB), mouse anti-MyHC, MF-20 (1:20, DSHB), and Alexa fluor 488 goat anti-mouse IgG (Life Technologies, 1:400) (secondary). Microscopy on fixed and stained zebrafish was carried out using a Leica DM 5500 microscope.

## ACCESSION NUMBERS

The Gene Expression Omnibus accession number for the microarray expression data is GSE66353.

## SUPPLEMENTAL INFORMATION

Supplemental Information includes Supplemental Experimental Procedures, seven figures, and one table and can be found with this article online at http://dx.doi.org/10.1016/j.molcel.2015.03.009.

**Figure 7. PI Modulates the Interaction of TAF3 PHD Finger with H3K4me3 and the Role of a Conserved PIP4K/PI5P/TAF3 in Zebrafish**

(A and B) Interaction of increasing concentrations of WT-TAF3 (A) and KK-TAF3 (B) PHD finger with fluorescent H3K4me3 peptide in the absence (black line) and presence (green line) of PI5P. The table indicates apparent KD values for the interactions. PI5P significantly (p = 0.0008) changed the KD of the interaction of WT-TAF3 but not KK-TAF3 PHD finger with H3K4me3 peptide.

(C) TAF3 ChIP analysis at the genes indicated of shX or sh-TAF3 C2C12 cells. The data are shown as enrichment over the IgG control and represent the mean of triplicates + SD.

(D) RNAPII and TAF3 ChIP analysis at the genes indicated of control (−) or sh-PIP4K2B (+) cells before and after differentiation for 48 hr. Data are presented as mean +SEM (RNAPII n = 5 and TAF3 n = 2). The data were analyzed by a one way ANOVA with a post Hoc Dunnets test to compare conditions to the control sample (undifferentiated SHX). *p < 0.05.

(E) RNAPII and TAF3 ChIP analysis at the promoter of the MYH gene of shX or shPIP4K2B C2C12 cells before and after differentiation for 48 hr and 72 hr. Data are presented as means +SEM (n = 2). Data are presented as mean +SEM (RNAPII n = 5 and TAF3 n = 2). The data were analyzed with one-way ANOVA. *p < 0.05.

(F) C2C12 cells depicted in Figure 6A were analyzed by ChIP for the presence of RNAPII and TAF3 at the genomic regions indicated after differentiation for 48 hr. The data are fold changes over the WT-TAF3 sample and are presented as means +SEM (n = 2). The data were analyzed with one-way ANOVA. *p < 0.05.

(G) Zebrafish embryos injected with control MO or PIP4K targeting MO. PIP4K MO injected embryos were also co-injected with RNA encoding the wild-type human PIP4K2A (WT-PIP4K) or the kinase inactive enzyme (KD-PIP4K). Embryos were collected 24 hr post-fertilization. Left: representative images are shown. Right: QRT-PCR analysis of mRNA isolated from injected embryos for TAF3 or *mespa* (a direct TAF3 target) expression. The data represent fold changes compared to control and represent the mean of triplicates +SD, and were normalized to GAPDH.

(H) Zebrafish embryos injected with control MO or TAF3 targeting MO. TAF3 MO injected embryos were also co-injected with RNA encoding human WT-TAF3, or mutant TAF3 constructs unable to interact with PI (KK-TAF3) and methylated histone H3 (DW-TAF3). Embryos were collected 24 hr post-fertilization and stained using F59 (MYHC). Representative images of the disruption of the myosin filament architecture by the indicated injections are shown. The severity of the phenotypes was categorized into strong and weak and presented graphically. The number of injected embryos is indicated above each graph.

(I) Schematic showing that interaction of TAF3 with nuclear PI regulated by PIP4K2B modulates transcriptional output. PIP4K2B phosphorylates and regulates the levels of nuclear PI5P and a small pool of $PI(4,5)P_2$. Knockdown of PIP4K2B increases PI5P levels that interact with and regulate TAF3 transcriptional complexes. PI interaction with TAF3 can lead to both upregulation and downregulation of specific genes, which eventually affects cell fates such as myoblast differentiation.

See also Figure S7.

## AUTHOR CONTRIBUTIONS

S.-B.Y. performed the initial PHD interaction screen, experiments to determine the role of PIP4K2B and its relationship with TAF3 in myoblast differentiation, and the TAF3 ChIP. L.S. performed SPR analysis of PHD fingers, characterized the interaction of TAF3 with PI and its role in differentiation, and edited the manuscript. M.T., K.A.G., Z.H., and W.F. analyzed the interaction of TAF3 with H3K4me3. M.B. and D.H. analyzed the role of TAF3 in Zebrafish, and P.G., J.v.P., S.-B.Y., and A.-P.G.H. analyzed the role of PIP4K in mespa regulation. H.R., C.T., and S.M.L. provided the TAF3 antibody and carried out the ChIP experiments. K.E.A., D.R.J., and P.H. analyzed nuclear PtdIns levels. The laboratories of W.F., A.-P.G.H., D.H., and S.M.L., contributed equally to this study. N.D. carried out the initial PHD screens, devised and analyzed the experiments, and wrote the manuscript.

## ACKNOWLEDGMENTS

We are grateful to the K.W.F. (Netherlands), CRUK (grant no. C5759/A12328), and Southampton University for funding and Prof Timmers (Utrecht University) for reagents and discussion. We would also like to thank colleagues from the Somervaille lab (PICR), the department of biological sciences (U.O.S.) and Y. Yong (PICR) for useful discussions. The work in the lab of W.F. was funded by the Max Planck Society and the Deutsche Forschungsgemeinschaft (grant no. 564967). A.-P.G.H. was funded by the Dutch Cancer Society (KWF) (grant UL 2012-5395). This work was supported in part by the American Cancer Society ACS-IRG #70-002 (S.M.L.). K.A. and P.H. acknowledge support from Biotechnology and Biological Research Council (ISPG BB/J004456/1).

Received: August 9, 2014
Revised: January 20, 2015
Accepted: March 6, 2015
Published: April 9, 2015

## REFERENCES

Asp, P., Blum, R., Vethantham, V., Parisi, F., Micsinai, M., Cheng, J., Bowman, C., Kluger, Y., and Dynlacht, B.D. (2011). Genome-wide remodeling of the epigenetic landscape during myogenic differentiation. Proc. Natl. Acad. Sci. USA *108*, E149–E158.

Bidlingmaier, S., and Liu, B. (2007). Interrogating yeast surface-displayed human proteome to identify small molecule-binding proteins. Mol. Cell. Proteomics *6*, 2012–2020.

Bidlingmaier, S., Wang, Y., Liu, Y., Zhang, N., and Liu, B. (2011). Comprehensive analysis of yeast surface displayed cDNA library selection outputs by exon microarray to identify novel protein-ligand interactions. Mol. Cell. Proteomics *10*, 005116.

Bienz, M. (2006). The PHD finger, a nuclear protein-interaction domain. Trends Biochem. Sci. *31*, 35–40.

Blind, R.D., Suzawa, M., and Ingraham, H.A. (2012). Direct modification and activation of a nuclear receptor-$PIP_2$ complex by the inositol lipid kinase IPMK. Sci. Signal. *5*, ra44.

Boronenkov, I.V., Loijens, J.C., Umeda, M., and Anderson, R.A. (1998). Phosphoinositide signaling pathways in nuclei are associated with nuclear speckles containing pre-mRNA processing factors. Mol. Biol. Cell *9*, 3547–3560.

Bultsma, Y., Keune, W.J., and Divecha, N. (2010). PIP4Kbeta interacts with and modulates nuclear localization of the high-activity PtdIns5P-4-kinase isoform PIP4Kalpha. Biochem. J. *430*, 223–235.

Burley, S.K., and Roeder, R.G. (1996). Biochemistry and structural biology of transcription factor IID (TFIID). Annu. Rev. Biochem. *65*, 769–799.

Ciruela, A., Hinchliffe, K.A., Divecha, N., and Irvine, R.F. (2000). Nuclear targeting of the beta isoform of type II phosphatidylinositol phosphate kinase (phosphatidylinositol 5-phosphate 4-kinase) by its alpha-helix 7. Biochem. J. *346*, 587–591.

Clark, J., Anderson, K.E., Juvin, V., Smith, T.S., Karpe, F., Wakelam, M.J., Stephens, L.R., and Hawkins, P.T. (2011). Quantification of PtdInsP3 molecular species in cells and tissues by mass spectrometry. Nat. Methods *8*, 267–272.

Clarke, J.H., Letcher, A.J., D'santos, C.S., Halstead, J.R., Irvine, R.F., and Divecha, N. (2001). Inositol lipids are regulated during cell cycle progression in the nuclei of murine erythroleukaemia cells. Biochem. J. *357*, 905–910.

Clarke, J.H., Richardson, J.P., Hinchliffe, K.A., and Irvine, R.F. (2007). Type II PtdInsP kinases: location, regulation and function. Biochem. Soc. Symp. *74*, 149–159.

Deato, M.D., and Tjian, R. (2007). Switching of the core transcription machinery during myogenesis. Genes Dev. *21*, 2137–2149.

Deato, M.D., and Tjian, R. (2008). An unexpected role of TAFs and TRFs in skeletal muscle differentiation: switching core promoter complexes. Cold Spring Harb. Symp. Quant. Biol. *73*, 217–225.

Deato, M.D., Marr, M.T., Sottero, T., Inouye, C., Hu, P., and Tjian, R. (2008). MyoD targets TAF3/TRF3 to activate myogenin transcription. Mol. Cell *32*, 96–105.

Divecha, N., Banfić, H., and Irvine, R.F. (1991). The polyphosphoinositide cycle exists in the nuclei of Swiss 3T3 cells under the control of a receptor (for IGF-I) in the plasma membrane, and stimulation of the cycle increases nuclear diacylglycerol and apparently induces translocation of protein kinase C to the nucleus. EMBO J. *10*, 3207–3214.

Divecha, N., Letcher, A.J., Banfic, H.H., Rhee, S.G., and Irvine, R.F. (1995). Changes in the components of a nuclear inositide cycle during differentiation in murine erythroleukaemia cells. Biochem. J. *312*, 63–67.

Elouarrat, D., van der Velden, Y.U., Jones, D.R., Moolenaar, W.H., Divecha, N., and Haramis, A.P. (2013). Role of phosphatidylinositol 5-phosphate 4-kinase $\alpha$ in zebrafish development. Int. J. Biochem. Cell Biol. *45*, 1293–1301.

Emerling, B.M., Hurov, J.B., Poulogiannis, G., Tsukazawa, K.S., Choo-Wing, R., Wulf, G.M., Bell, E.L., Shim, H.S., Lamia, K.A., Rameh, L.E., et al. (2013). Depletion of a putatively druggable class of phosphatidylinositol kinases inhibits growth of p53-null tumors. Cell *155*, 844–857.

Gelato, K.A., Tauber, M., Ong, M.S., Winter, S., Hiragami-Hamada, K., Sindlinger, J., Lemak, A., Bultsma, Y., Houliston, S., Schwarzer, D., et al. (2014). Accessibility of different histone H3-binding domains of UHRF1 is allosterically regulated by phosphatidylinositol 5-phosphate. Mol. Cell *54*, 905–919.

Gozani, O., Karuman, P., Jones, D.R., Ivanov, D., Cha, J., Lugovskoy, A.A., Baird, C.L., Zhu, H., Field, S.J., Lessnick, S.L., et al. (2003). The PHD finger of the chromatin-associated protein ING2 functions as a nuclear phosphoinositide receptor. Cell *114*, 99–111.

Hart, D.O., Santra, M.K., Raha, T., and Green, M.R. (2009). Selective interaction between Trf3 and Taf3 required for early development and hematopoiesis. Dev. Dyn. *238*, 2540–2549.

Hochheimer, A., and Tjian, R. (2003). Diversified transcription initiation complexes expand promoter selectivity and tissue-specific gene expression. Genes Dev. *17*, 1309–1320.

Jones, D.R., Bultsma, Y., Keune, W.J., Halstead, J.R., Elouarrat, D., Mohammed, S., Heck, A.J., D'Santos, C.S., and Divecha, N. (2006). Nuclear PtdIns5P as a transducer of stress signaling: an in vivo role for PIP4Kbeta. Mol. Cell *23*, 685–695.

Jones, D.R., Ramirez, I.B., Lowe, M., and Divecha, N. (2013). Measurement of phosphoinositides in the zebrafish Danio rerio. Nat. Protoc. *8*, 1058–1072.

Jude, J.G., Spencer, G.J., Huang, X., Somerville, T.D., Jones, D.R., Divecha, N., and Somervaille, T.C. (2014). A targeted knockdown screen of genes coding for phosphoinositide modulators identifies PIP4K2A as required for acute myeloid leukemia cell proliferation and survival. Oncogene.

Jungmichel, S., Sylvestersen, K.B., Choudhary, C., Nguyen, S., Mann, M., and Nielsen, M.L. (2014). Specificity and commonality of the phosphoinositide-binding proteome analyzed by quantitative mass spectrometry. Cell Rep. *6*, 578–591.

Keune, W.J., Sims, A.H., Jones, D.R., Bultsma, Y., Lynch, J.T., Jirström, K., Landberg, G., and Divecha, N. (2013). Low PIP4K2B expression in human breast tumors correlates with reduced patient survival: A role for PIP4K2B in the regulation of E-cadherin expression. Cancer Res. *73*, 6913–6925.

Lauberth, S.M., Bilyeu, A.C., Firulli, B.A., Kroll, K.L., and Rauchman, M. (2007). A phosphomimetic mutation in the Sall1 repression motif disrupts recruitment of the nucleosome remodeling and deacetylase complex and repression of Gbx2. J. Biol. Chem. *282*, 34858–34868.

Lauberth, S.M., Nakayama, T., Wu, X., Ferris, A.L., Tang, Z., Hughes, S.H., and Roeder, R.G. (2013). H3K4me3 interactions with TAF3 regulate preinitiation complex assembly and selective gene activation. Cell *152*, 1021–1036.

Lessard, J., Wu, J.I., Ranish, J.A., Wan, M., Winslow, M.M., Staahl, B.T., Wu, H., Aebersold, R., Graef, I.A., and Crabtree, G.R. (2007). An essential switch in subunit composition of a chromatin remodeling complex during neural development. Neuron *55*, 201–215.

Lewis, A.E., Sommer, L., Arntzen, M.O., Strahm, Y., Morrice, N.A., Divecha, N., and D'Santos, C.S. (2011). Identification of nuclear phosphatidylinositol 4,5-bisphosphate-interacting proteins by neomycin extraction. Mol. Cell. Proteomics *10*, 003376.

Lindsay, Y., McCoull, D., Davidson, L., Leslie, N.R., Fairservice, A., Gray, A., Lucocq, J., and Downes, C.P. (2006). Localization of agonist-sensitive PtdIns(3,4,5)P3 reveals a nuclear pool that is insensitive to PTEN expression. J. Cell Sci. *119*, 5160–5168.

Liu, Z., Scannell, D.R., Eisen, M.B., and Tjian, R. (2011). Control of embryonic stem cell lineage commitment by core promoter factor, TAF3. Cell *146*, 720–731.

Lower, K.M., Turner, G., Kerr, B.A., Mathews, K.D., Shaw, M.A., Gedeon, A.K., Schelley, S., Hoyme, H.E., White, S.M., Delatycki, M.B., et al. (2002). Mutations in PHF6 are associated with Börjeson-Forssman-Lehmann syndrome. Nat. Genet. *32*, 661–665.

Maffucci, T. (2012). An introduction to phosphoinositides. Curr. Top. Microbiol. Immunol. *362*, 1–42.

Maston, G.A., Zhu, L.J., Chamberlain, L., Lin, L., Fang, M., and Green, M.R. (2012). Non-canonical TAF complexes regulate active promoters in human embryonic stem cells. eLife *1*, e00068.

Meijer, H.J., Berrie, C.P., Iurisci, C., Divecha, N., Musgrave, A., and Munnik, T. (2001). Identification of a new polyphosphoinositide in plants, phosphatidylinositol 5-monophosphate (PtdIns5P), and its accumulation upon osmotic stress. Biochem. J. *360*, 491–498.

Mellman, D.L., Gonzales, M.L., Song, C., Barlow, C.A., Wang, P., Kendziorski, C., and Anderson, R.A. (2008). A PtdIns4,5P2-regulated nuclear poly(A) polymerase controls expression of select mRNAs. Nature *451*, 1013–1017.

Musselman, C.A., and Kutateladze, T.G. (2009). PHD fingers: epigenetic effectors and potential drug targets. Mol. Interv. *9*, 314–323.

Ndamukong, I., Jones, D.R., Lapko, H., Divecha, N., and Avramova, Z. (2010). Phosphatidylinositol 5-phosphate links dehydration stress to the activity of arabidopsis trithorax-like factor ATX1. PLoS ONE *5*, e13396.

Pena, P.V., Davrazou, F., Shi, X., Walter, K.L., Verkhusha, V.V., Gozani, O., Zhao, R., and Kutateladze, T.G. (2006a). Molecular mechanism of histone H3K4me3 recognition by plant homeodomain of ING2. Nature *442*, 100–103.

Peña, P.V., Davrazou, F., Shi, X., Walter, K.L., Verkhusha, V.V., Gozani, O., Zhao, R., and Kutateladze, T.G. (2006b). Molecular mechanism of histone H3K4me3 recognition by plant homeodomain of ING2. Nature *442*, 100–103.

Rajakumara, E., Wang, Z., Ma, H., Hu, L., Chen, H., Lin, Y., Guo, R., Wu, F., Li, H., Lan, F., et al. (2011). PHD finger recognition of unmodified histone H3R2 links UHRF1 to regulation of euchromatic gene expression. Mol. Cell *43*, 275–284.

Rameh, L.E., Tolias, K.F., Duckworth, B.C., and Cantley, L.C. (1997). A new pathway for synthesis of phosphatidylinositol-4,5-bisphosphate. Nature *390*, 192–196.

Richardson, J.P., Wang, M., Clarke, J.H., Patel, K.J., and Irvine, R.F. (2007). Genomic tagging of endogenous type IIbeta phosphatidylinositol 5-phosphate 4-kinase in DT40 cells reveals a nuclear localisation. Cell. Signal. *19*, 1309–1314.

Shah, Z.H., Jones, D.R., Sommer, L., Foulger, R., Bultsma, Y., D'Santos, C., and Divecha, N. (2013). Nuclear phosphoinositides and their impact on nuclear functions. FEBS J. *280*, 6295–6310.

Shi, X., Hong, T., Walter, K.L., Ewalt, M., Michishita, E., Hung, T., Carney, D., Peña, P., Lan, F., Kaadige, M.R., et al. (2006). ING2 PHD domain links histone H3 lysine 4 methylation to active gene repression. Nature *442*, 96–99.

Subramanian, A., Tamayo, P., Mootha, V.K., Mukherjee, S., Ebert, B.L., Gillette, M.A., Paulovich, A., Pomeroy, S.L., Golub, T.R., Lander, E.S., and Mesirov, J.P. (2005). Gene set enrichment analysis: a knowledge-based approach for interpreting genome-wide expression profiles. Proc. Natl. Acad. Sci. USA *102*, 15545–15550.

Thomas, M.C., and Chiang, C.M. (2006). The general transcription machinery and general cofactors. Crit. Rev. Biochem. Mol. Biol. *41*, 105–178.

van den Bout, I., and Divecha, N. (2009). PIP5K-driven PtdIns(4,5)P2 synthesis: regulation and cellular functions. J. Cell Sci. *122*, 3837–3850.

van Oevelen, C., Bowman, C., Pellegrino, J., Asp, P., Cheng, J., Parisi, F., Micsinai, M., Kluger, Y., Chu, A., Blais, A., et al. (2010). The mammalian Sin3 proteins are required for muscle development and sarcomere specification. Mol. Cell. Biol. *30*, 5686–5697.

Van Vlierberghe, P., Palomero, T., Khiabanian, H., Van der Meulen, J., Castillo, M., Van Roy, N., De Moerloose, B., Philippé, J., González-García, S., Toribio, M.L., et al. (2010). PHF6 mutations in T-cell acute lymphoblastic leukemia. Nat. Genet. *42*, 338–342.

Varier, R.A., Outchkourov, N.S., de Graaf, P., van Schaik, F.M., Ensing, H.J., Wang, F., Higgins, J.M., Kops, G.J., and Timmers, H.T. (2010). A phospho/methyl switch at histone H3 regulates TFIID association with mitotic chromosomes. EMBO J. *29*, 3967–3978.

Vermeulen, M., Mulder, K.W., Denissov, S., Pijnappel, W.W., van Schaik, F.M., Varier, R.A., Baltissen, M.P., Stunnenberg, H.G., Mann, M., and Timmers, H.T. (2007). Selective anchoring of TFIID to nucleosomes by trimethylation of histone H3 lysine 4. Cell *131*, 58–69.

Wang, M., Bond, N.J., Letcher, A.J., Richardson, J.P., Lilley, K.S., Irvine, R.F., and Clarke, J.H. (2010). Genomic tagging reveals a random association of endogenous PtdIns5P 4-kinases IIalpha and IIbeta and a partial nuclear localization of the IIalpha isoform. Biochem. J. *430*, 215–221.

Watt, S.A., Kimber, W.A., Fleming, I.N., Leslie, N.R., Downes, C.P., and Lucocq, J.M. (2004). Detection of novel intracellular agonist responsive pools of phosphatidylinositol 3,4-bisphosphate using the TAPP1 pleckstrin homology domain in immunoelectron microscopy. Biochem. J. *377*, 653–663.

Wysocka, J., Swigut, T., Xiao, H., Milne, T.A., Kwon, S.Y., Landry, J., Kauer, M., Tackett, A.J., Chait, B.T., Badenhorst, P., et al. (2006). A PHD finger of NURF couples histone H3 lysine 4 trimethylation with chromatin remodelling. Nature *442*, 86–90.

Yoshida, N., Yoshida, S., Koishi, K., Masuda, K., and Nabeshima, Y. (1998). Cell heterogeneity upon myogenic differentiation: down-regulation of MyoD and Myf-5 generates 'reserve cells'. J. Cell Sci. *111*, 769–779.

**Molecular Cell, Volume *58***

## Supplemental Information

**The Basal Transcription Complex Component TAF3 Transduces Changes in Nuclear Phosphoinositides into Transcriptional Output**

Yvette Stijf-Bultsma, Lilly Sommer, Maria Tauber, Mai Baalbaki, Panagiota Giardoglou, David R. Jones, Kathy A. Gelato, Jason van Pelt, Zahid Shah, Homa Rahnamoun, Clara Toma, Karen E. Anderson, Philip Hawkins, Shannon M. Lauberth, Anna-Pavlina G. Haramis, Daniel Hart, Wolfgang Fischle, and Nullin Divecha

# figure S1 A

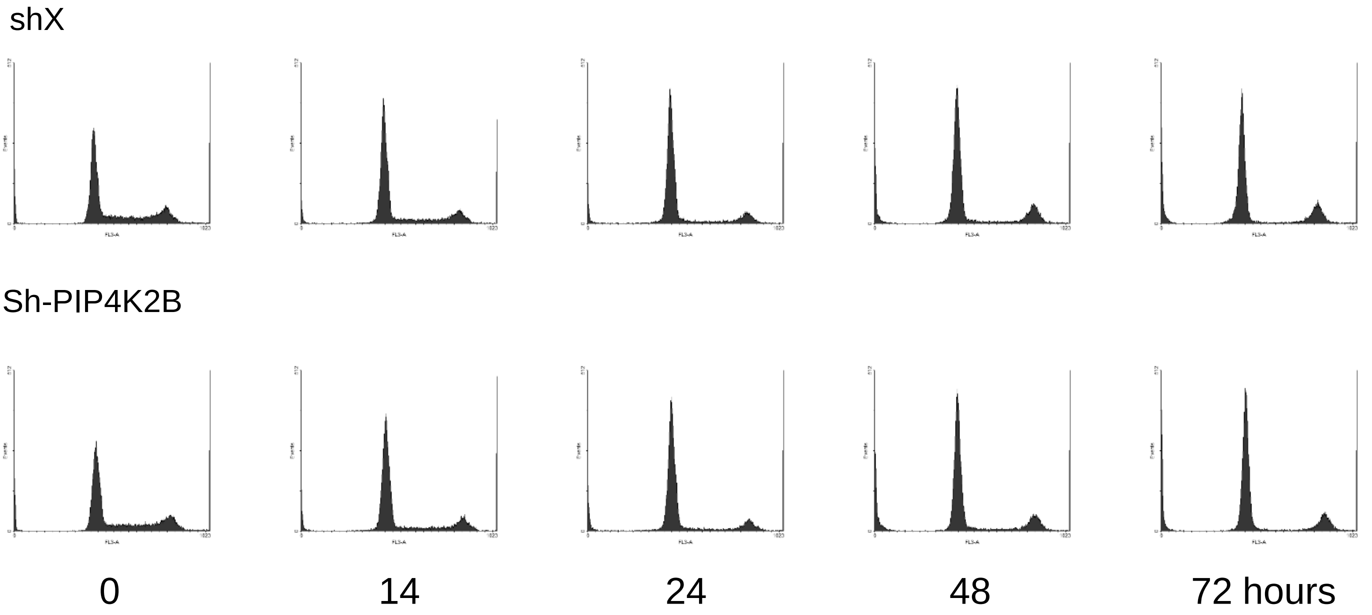


# figure S1 B

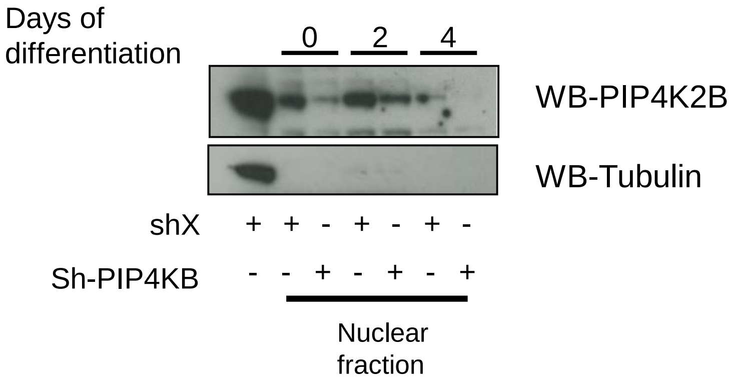


# figure S1 C

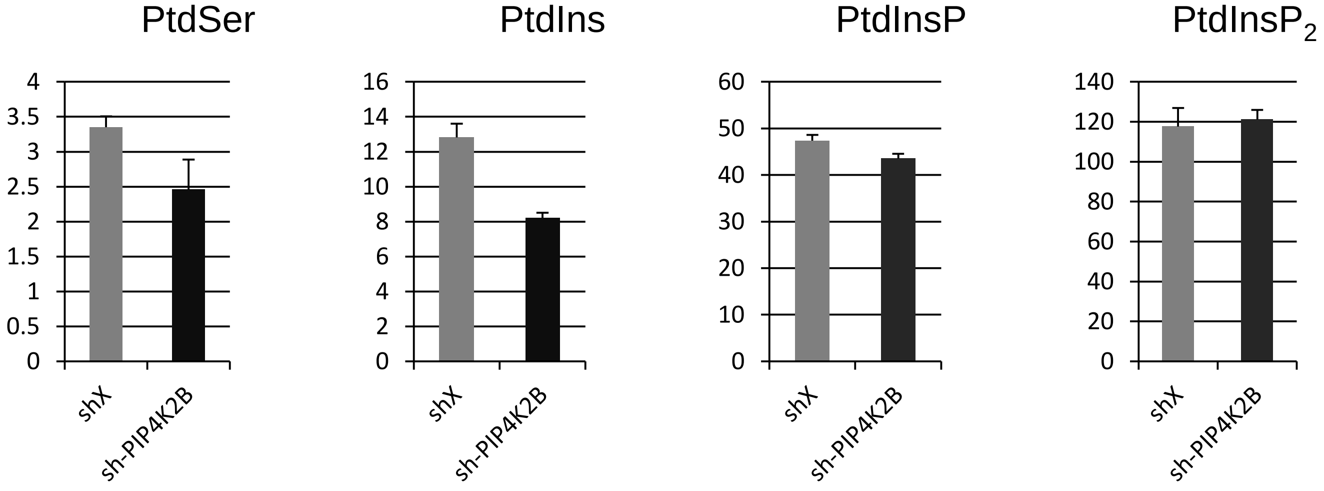

# figure S1 D

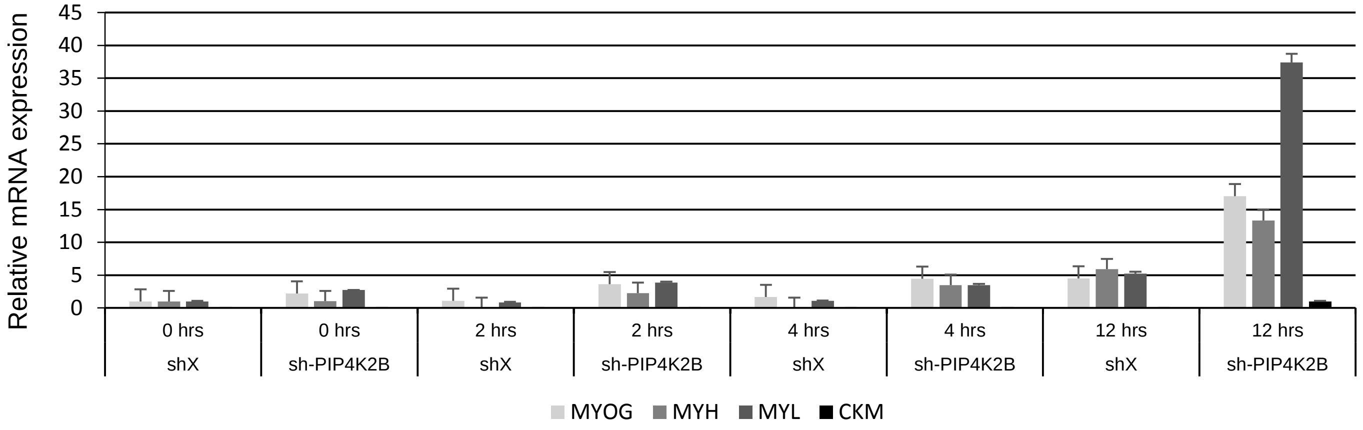


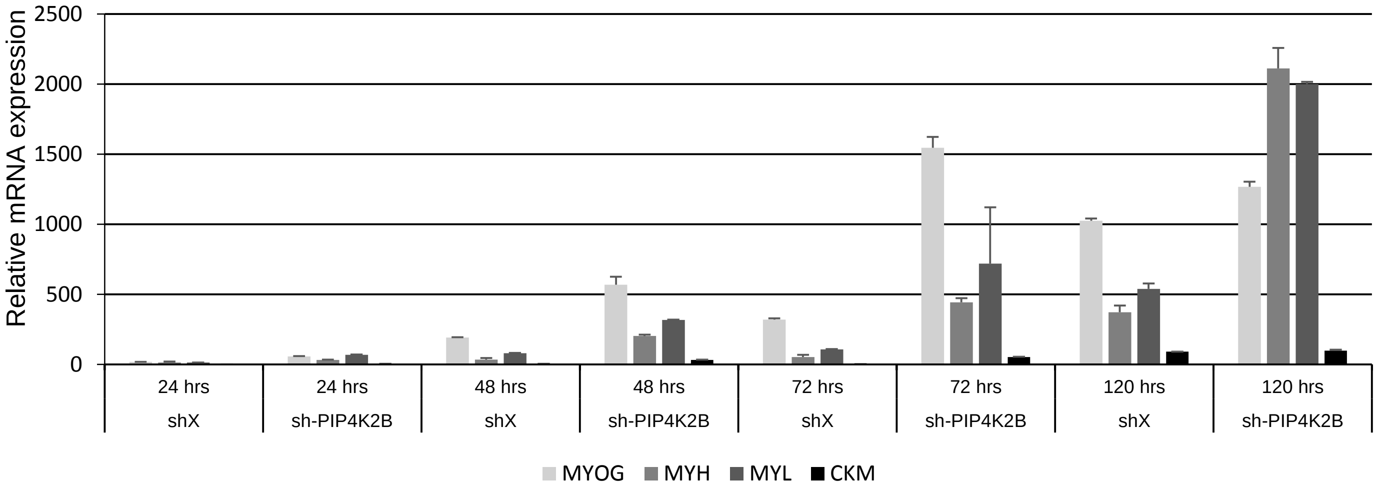


# figure S1 E

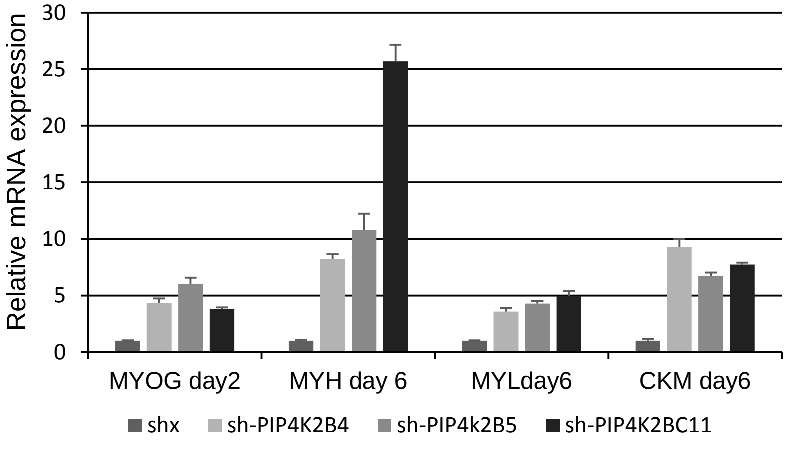


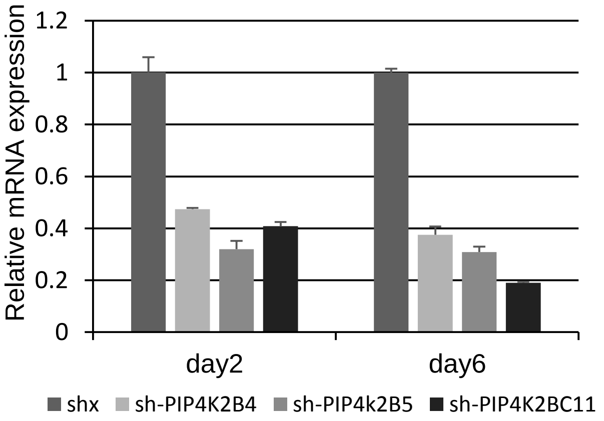

# figure S1 F

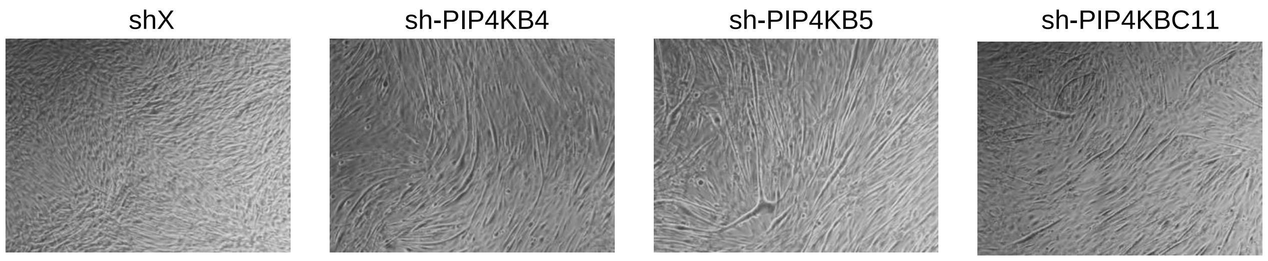


# figure S1 G

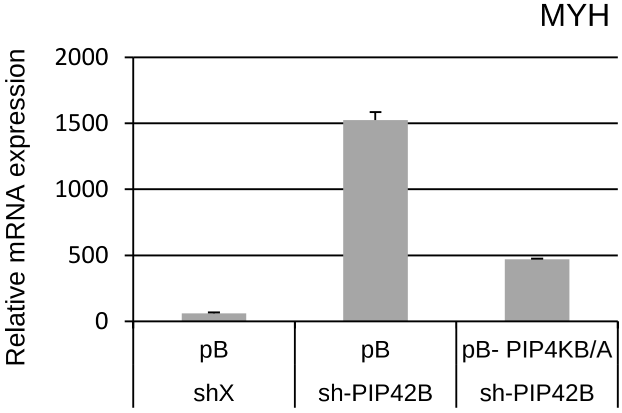


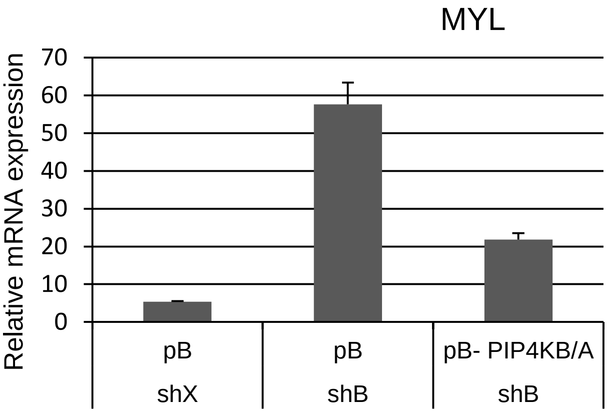


# figure S1 H

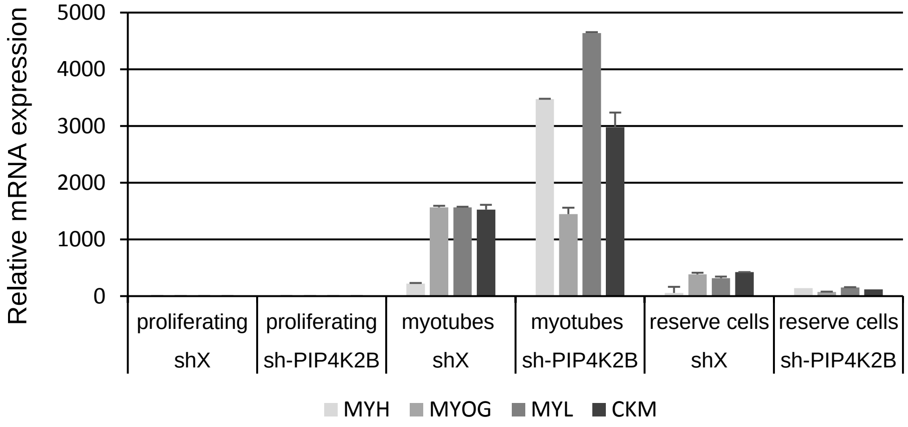

# Figure S2

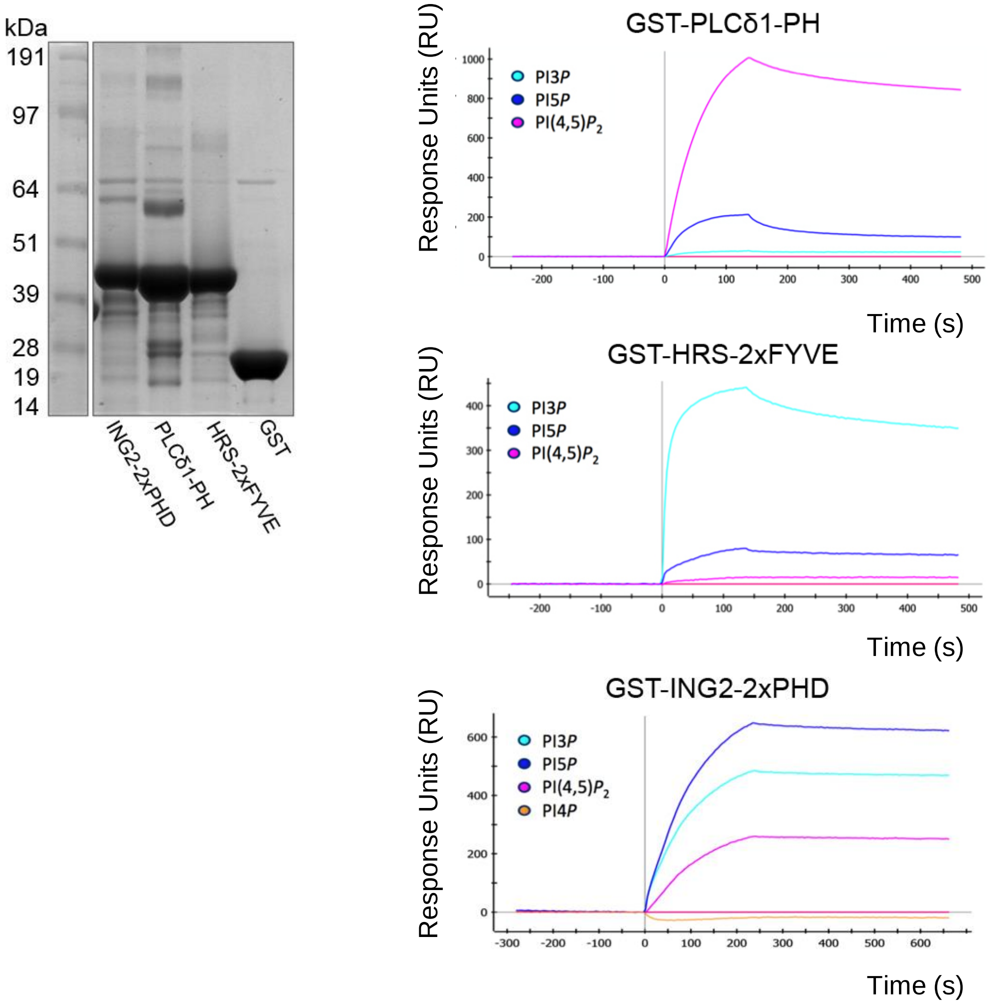
kDa
191
97
64
51
39
28
19
14
ING2-2xPHD
PLCδ1-PH
HRS-2xFYVE
GST
GST-PLCδ1-PH
Response Units (RU)
PI3P
PI5P
PI(4,5)P2
Time (s)
GST-HRS-2xFYVE
Response Units (RU)
PI3P
PI5P
PI(4,5)P2
Time (s)
GST-ING2-2xPHD
Response Units (RU)
PI3P
PI5P
PI(4,5)P2
PI4P
Time (s)

# Figure S2A

| | PHD-TAF3 | PBR |
|---|---|---|
| Zinc-finger mutant (C2/H5/C4/C7) | SAKAPVRSVVTETVSTYVIRDEWGNQIWICPGSNKPDDGSPMIGCDDSDDWYSWPCVGIMAAPP EEMQWFCPKSAN | KIKKDKKHKKRKHRAH |

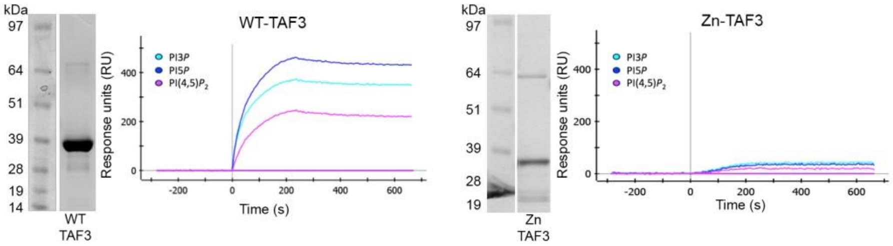


# Figure S3 B

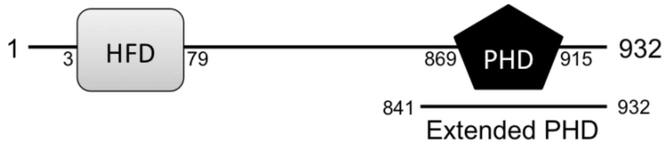


PHD-TAF3 WT

**Zinc finger** and polybasic region (PBR)

```
841 S A K A P V R S V V T E T V S T Y V I R
    D E W G N Q I W I C P G C N K P D D G S
    P M I G C D D C D D W Y H W P C V G I M
    A A P P E E M Q W F C P K C A N K I K K
    D K K H K K R K H R A H   932
```

# Figure S3 C

Deletion 1

```
841 S A K A P V R S V V T E T V S T Y V I R
    D E W G N Q I W I C P G C N K P D D G S
    P M I G C D D C D D W Y H W P C V G I M
    A A P P E E M Q W F C P K C A N   916
```

Deletion 2

```
841 S A K A P V R S V V T E T V S T Y V I R
    D E W G N Q I W I C P G C N K P D D G S
    P M I G C D D C D D W Y H W P C V G I M
    A A P P E E M Q W F C P K C A N K I K K   920
```

Deletion 3

```
841 S A K A P V R S V V T E T V S T Y V I R
    D E W G N Q I W I C P G C N K P D D G S
    P M I G C D D C D D W Y H W P C V G I M
    A A P P E E M Q W F C P K C A N K I K K
    D K K H   924
```

Deletion 4

```
841 S A K A P V R S V V T E T V S T Y V I R
    D E W G N Q I W I C P G C N K P D D G S
    P M I G C D D C D D W Y H W P C V G I M
    A A P P E E M Q W F C P K C A N K I K K
    D K K H K K R K   928
```

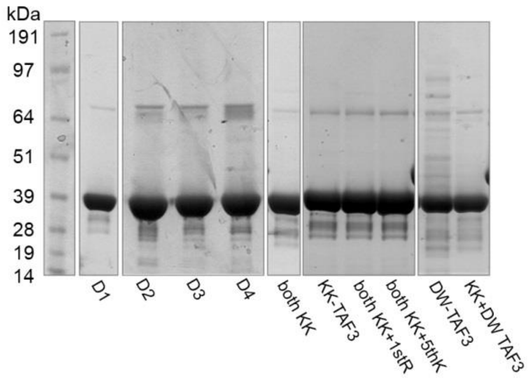


both KK

```
841 S A K A P V R S V V T E T V S T Y V I R
    D E W G N Q I W I C P G C N K P D D G S
    P M I G C D D C D D W Y H W P C V G I M
    A A P P E E M Q W F C P K C A N K I K K
    D A A H A A R K H R A H   932
```

both KK+5th K

```
841 S A K A P V R S V V T E T V S T Y V I R
    D E W G N Q I W I C P G C N K P D D G S
    P M I G C D D C D D W Y H W P C V G I M
    A A P P E E M Q W F C P K C A N K I K K
    D A A H A A R A H R A H   932
```

both KK+1st R

```
841 S A K A P V R S V V T E T V S T Y V I R
    D E W G N Q I W I C P G C N K P D D G S
    P M I G C D D C D D W Y H W P C V G I M
    A A P P E E M Q W F C P K C A N K I K K
    D A A H A A A K H R A H   932
```

PI binding mutant (KK-TAF3)
both KK+1stR+5thK

```
841 S A K A P V R S V V T E T V S T Y V I R
    D E W G N Q I W I C P G C N K P D D G S
    P M I G C D D C D D W Y H W P C V G I M
    A A P P E E M Q W F C P K C A N K I K K
    D A A H A A A A H R A H   932
```

Histone binding mutant (DW-TAF3)
D890A/W891A

```
841 S A K A P V R S V V T E T V S T Y V I R
    D E W G N Q I W I C P G C N K P D D G S
    P M I G C D D C D A A Y H W P C V G I M
    A A P P E E M Q W F C P K C A N K I K K
    D K K H K K R K H R A H   932
```

PI- and Histone binding mutant (KK+DW TAF3)
both KK+1stR+5thK+D890A/W891A

```
841 S A K A P V R S V V T E T V S T Y V I R
    D E W G N Q I W I C P G C N K P D D G S
    P M I G C D D C D A A Y H W P C V G I M
    A A P P E E M Q W F C P K C A N K I K K
    D A A H A A A A H R A H   932
```

# Figure S3 D

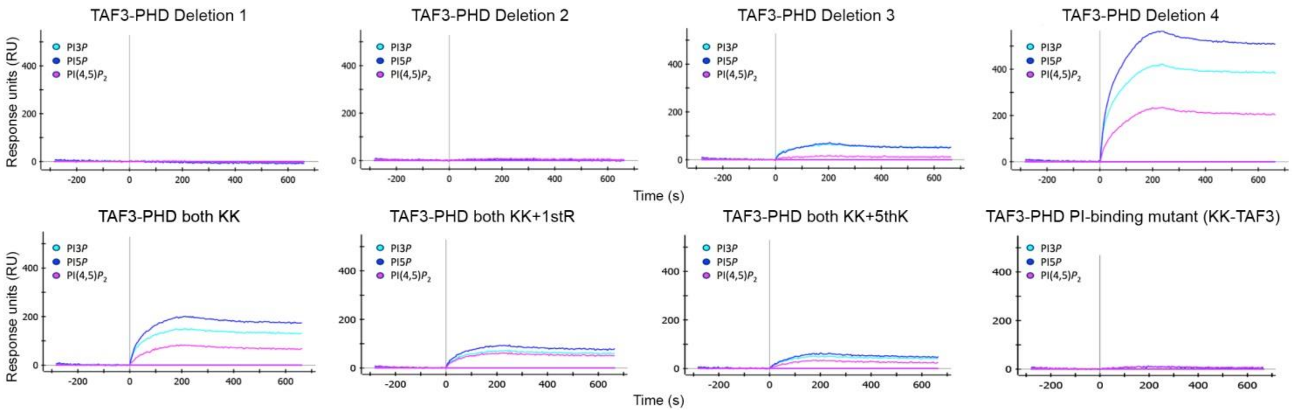


# Figure S3 E

## GST-PHD-TAF3 proteins

| Sequence description | Binding to phosphoinositides (as tested by SPR) | Binding to methylated histone peptides (as tested by SPR) |
|---|---|---|
| | **referenced to PC** | **referenced to unmodified H3 (methylation specific binding to H3 peptides)** |
| PHD wild-type | ✓✓✓ (very good binding) | ✓✓✓ binding to H3K4me3, followed by H3K4me2 and H3K4me1 |
| PHD Deletion 1 | ✗ (no binding) | ✓✓✓ |
| PHD Deletion 2 | ✗ | ✓✓✓ |
| PHD Deletion 3 | ✓ (binding) | ✓✓✓ |
| PHD Deletion 4 | ✓✓✓ | ✓✓✓ |
| PHD 1st KK mt | ✓✓✓ | ✓✓✓ |
| PHD 2nd KK mt | ✓✓✓ | ✓✓✓ |
| PHD both KK mt | ✓✓ (good binding) | ✓✓✓ |
| PHD 1stKK+4thK mt | ✓✓ | ✓✓✓ |
| PHD 2nd KK+1stK mt | ✓✓ | ✓✓✓ |
| PHD both KK+5thK mt | ✓ | ✓✓✓ |
| PHD both KK+1stR mt | ✓ | ✓✓✓ |
| PHD both KK+5thK+1stR mt = PI-binding mutant | ✗ | ✓✓✓ |
| PHD M882A | ✓✓✓ | ✗ (no binding) |
| PHD D886A | ✓✓✓ | ✗ |
| PHD D890A/W891A = Histone-binding mutant | ✓✓✓ | ✗ |
| PHD both KK+5thK+1stR+D890A/W891A = PI- and Histone-binding mutant | ✗ | ✗ |

# Figure S4 A

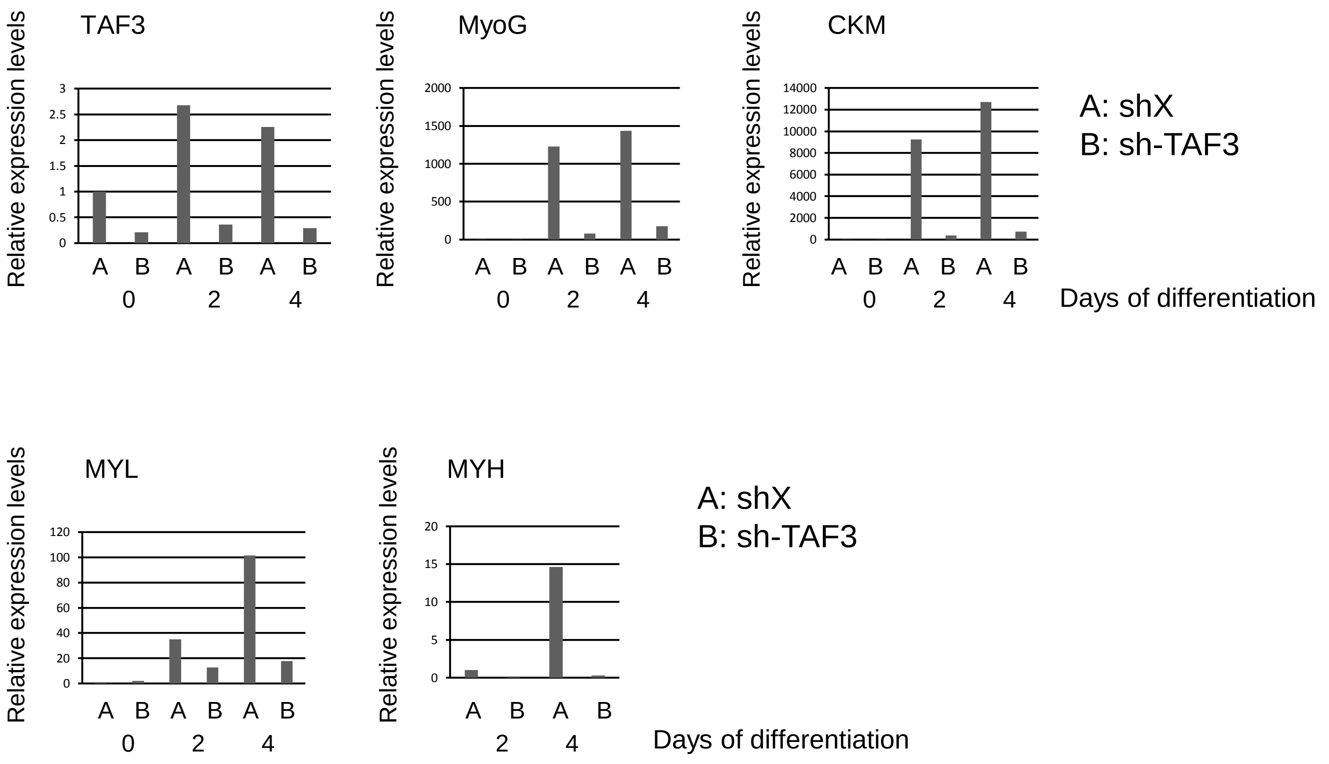


# Figure S4 B

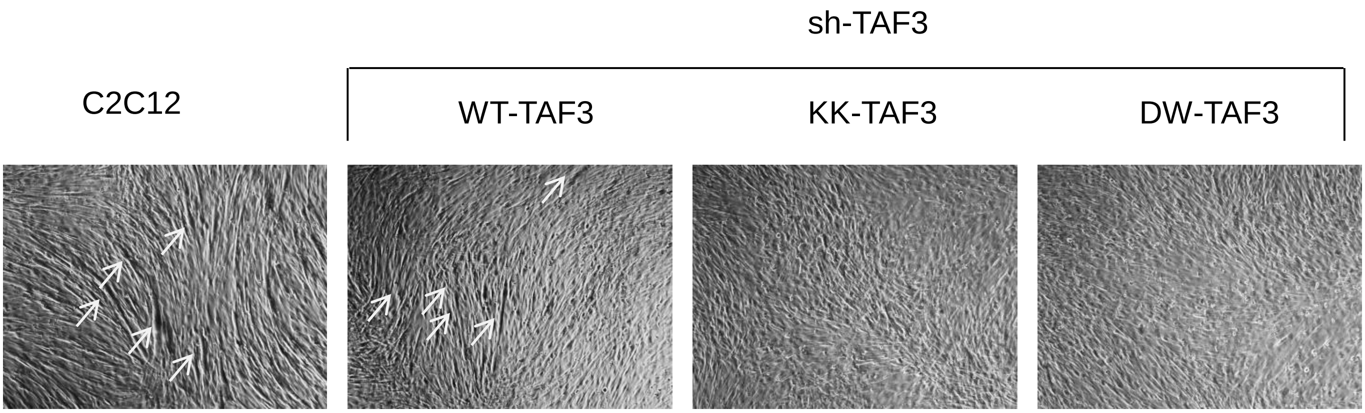

figure S5 A

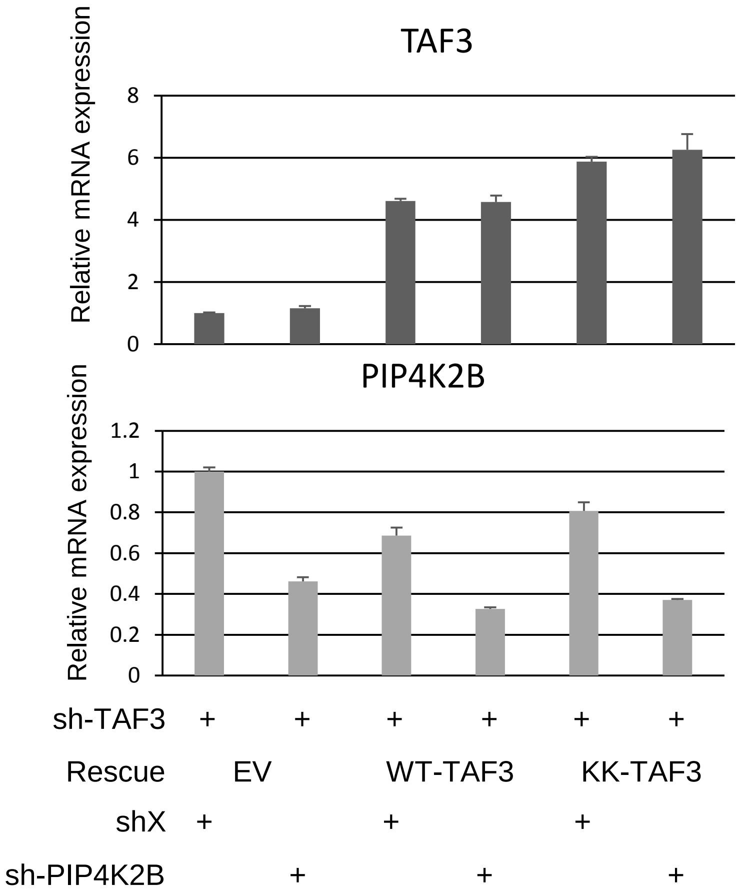


figure S5 B

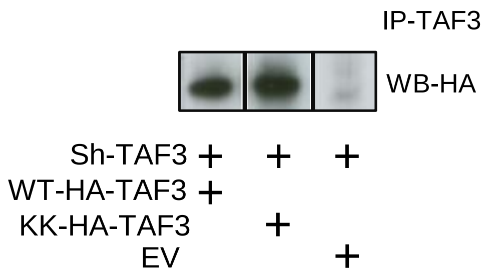


figure S5 C

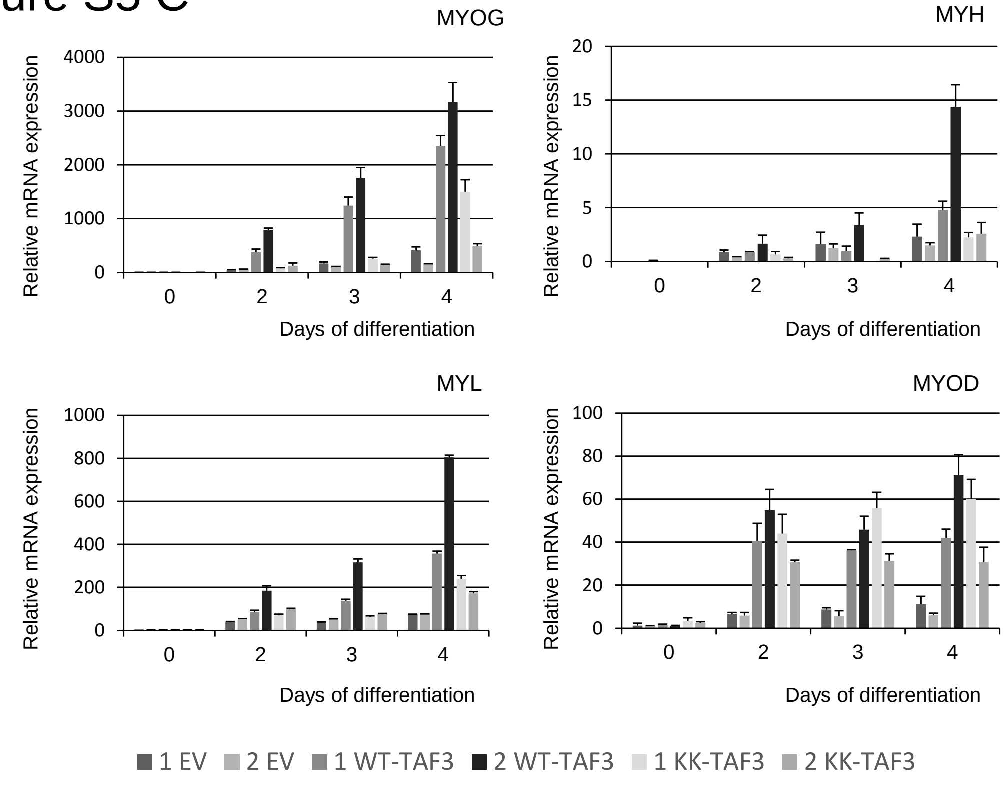

# figure S5 D

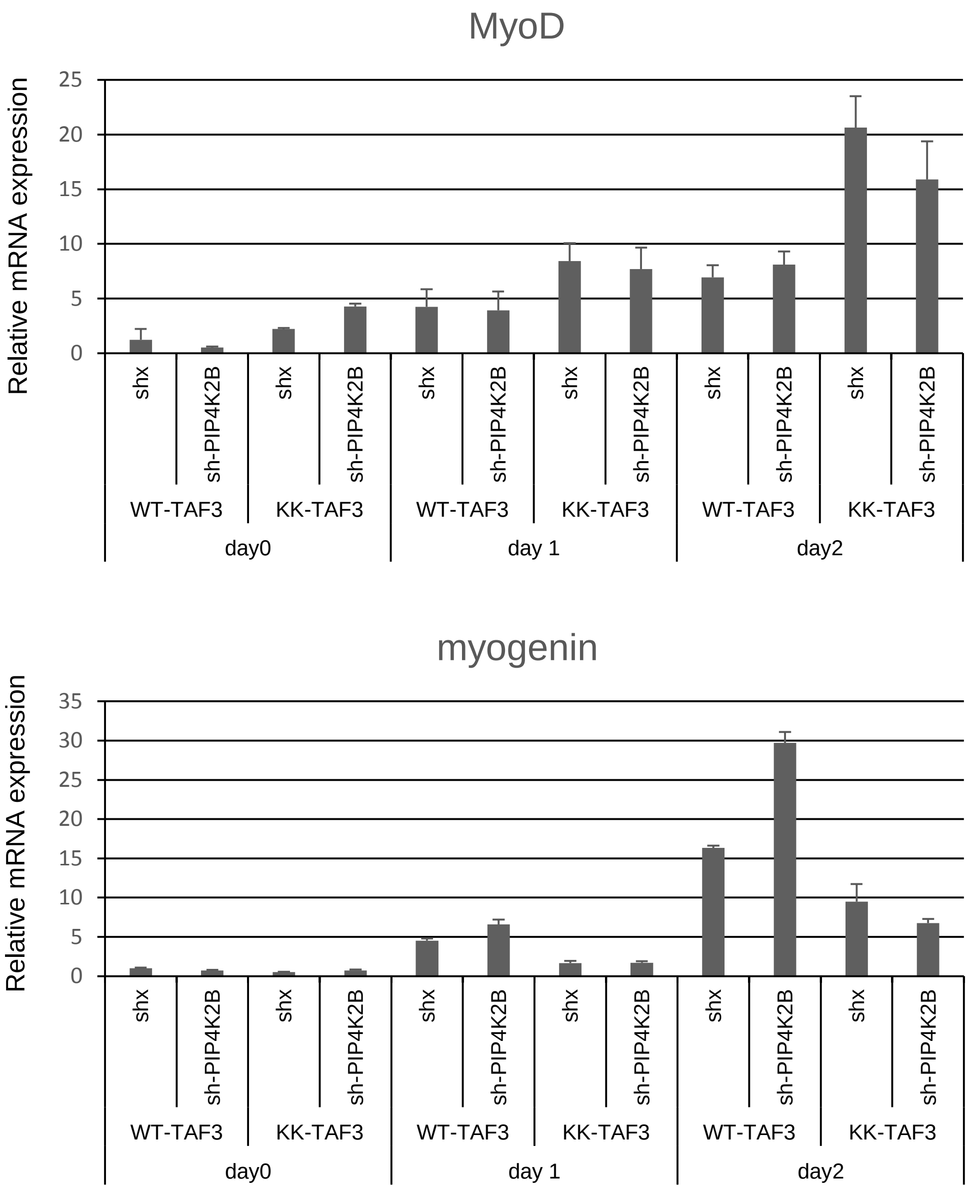
MyoD
Relative mRNA expression
25
20
15
10
5
0
shx
sh-PIP4K2B
WT-TAF3
KK-TAF3
day0
day 1
day2
myogenin
Relative mRNA expression
35
30
25
20
15
10
5
0
shx
sh-PIP4K2B
WT-TAF3
KK-TAF3
day0
day 1
day2

# figure S6A

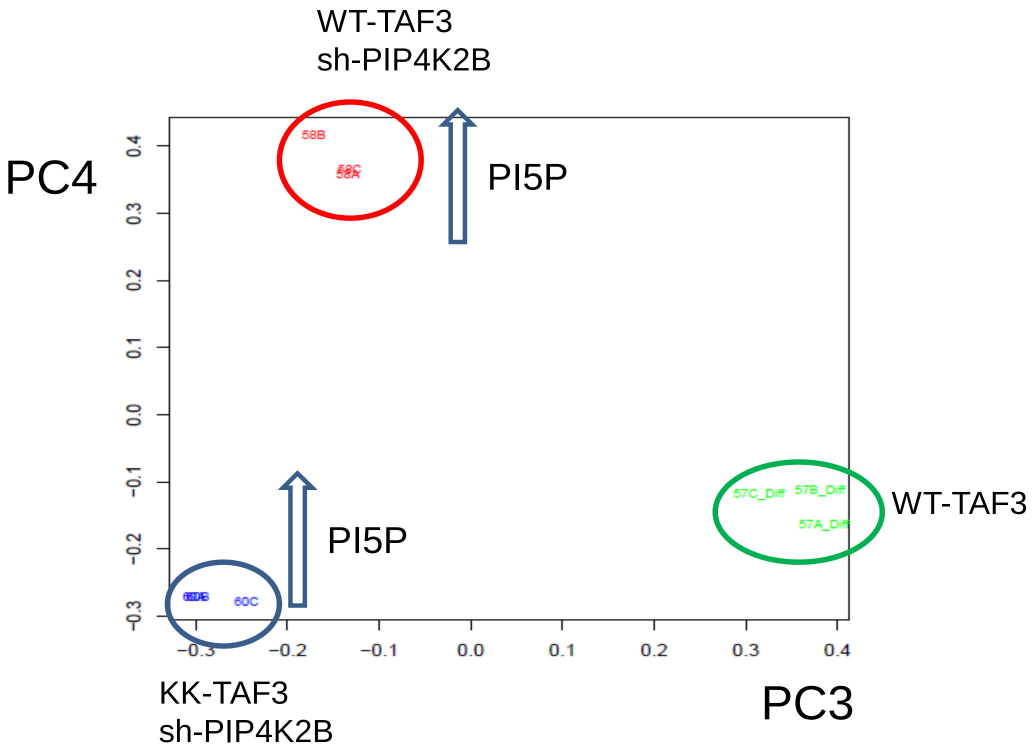


# figure S6B

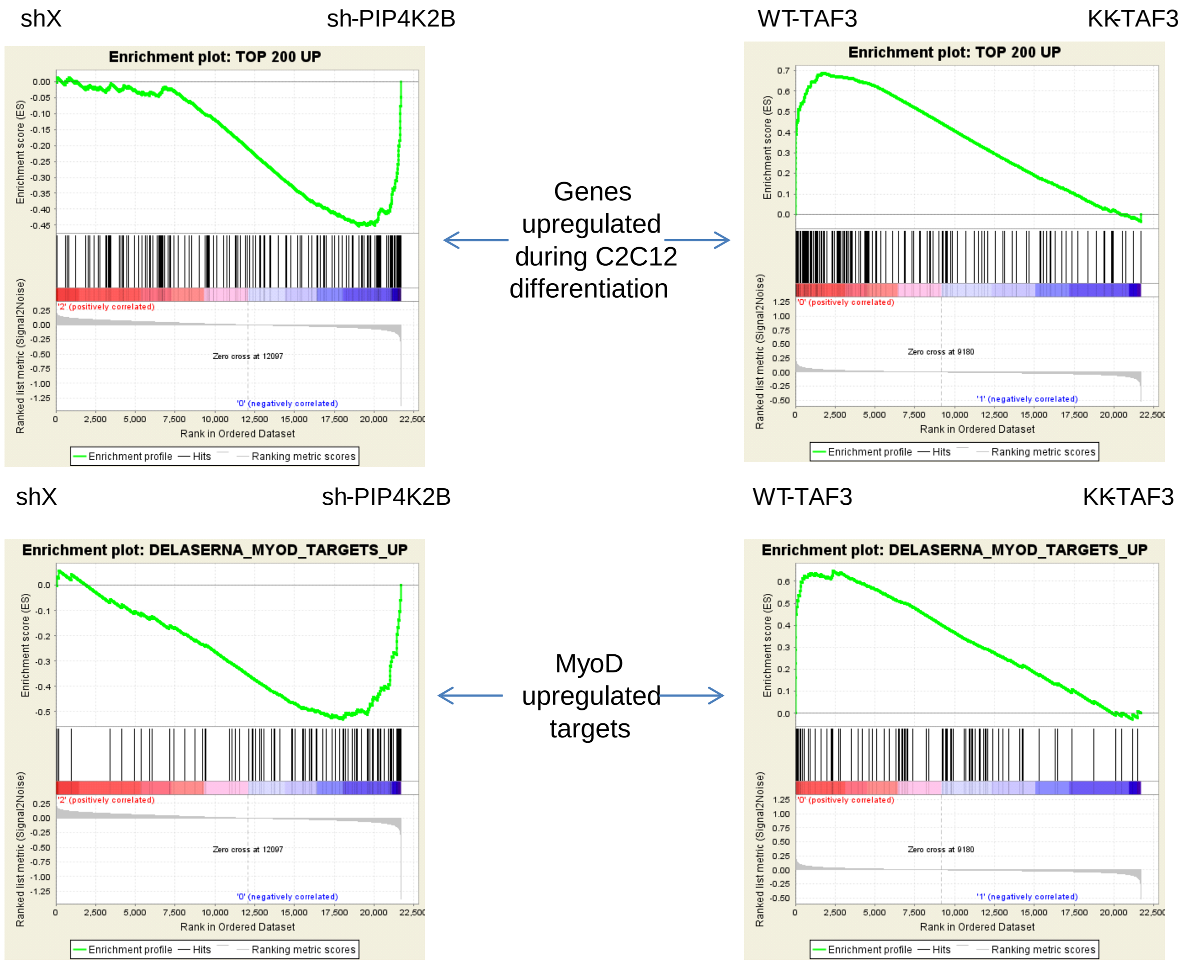

## figure S6 C

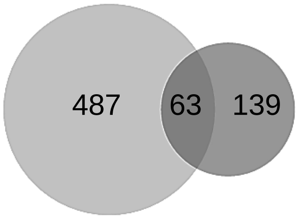


shX v sh-PIP4K2B WT-TAF3 v KK-TAF3

Myoblast differentiation

## figure S6 D

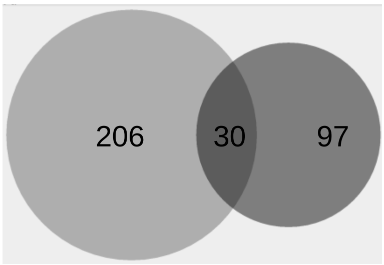


shX v sh-PIP4K2B WT-TAF3 v KK-TAF3

Etoposide

# figure S7 A

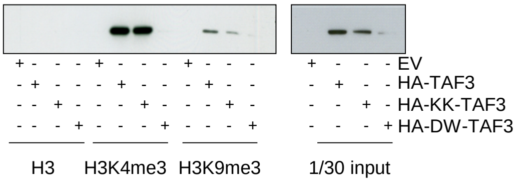


# figure S7 B

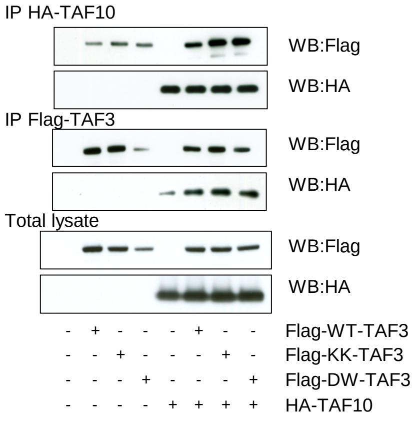


# figure S7 C

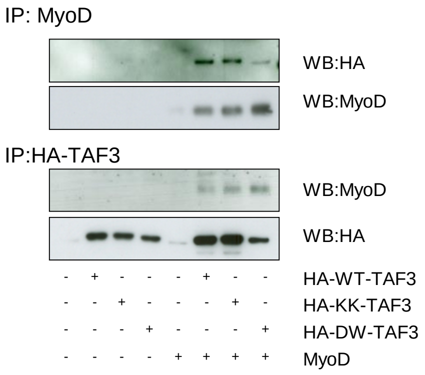


# figure S7 D

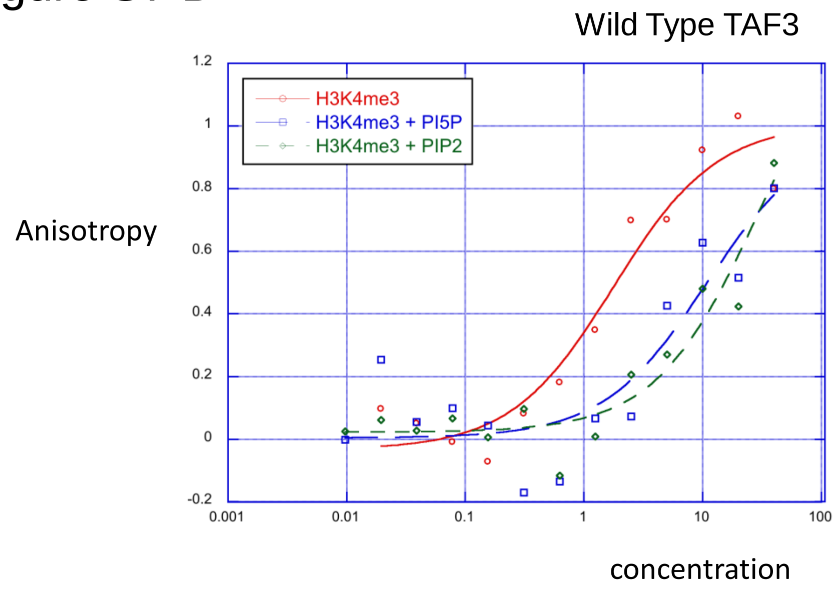


# figure S7 E

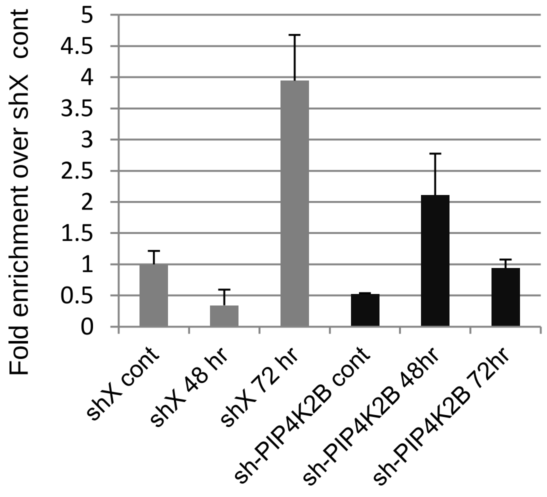

# figure S7 F

PBR of TAF3

| | |
|---|---|
| *Danio rerio* | 882 AGKKKDKKTKKRKRKAH 898 |
| *Mus Musculus* | 915 ANKKKDKKHKKRKHRAH 932 |
| *Homo sapiens* | 913 ANKKKDKKHKKRKHRAH 929 |
| *Xenopus laevis* | 913 ESKKKDKKHKKRKHKAH 929 |

# figure S7 G

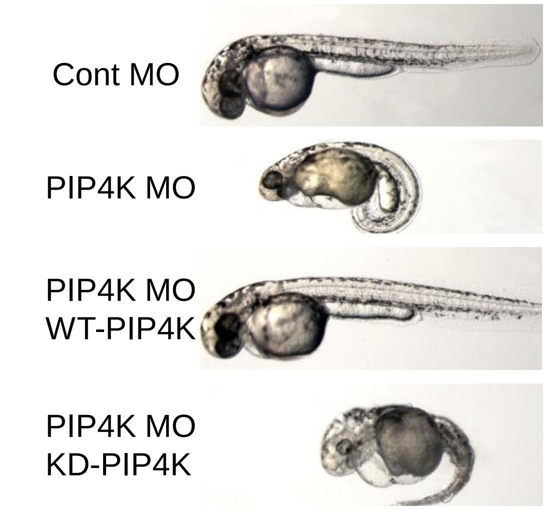


# figure S7 H

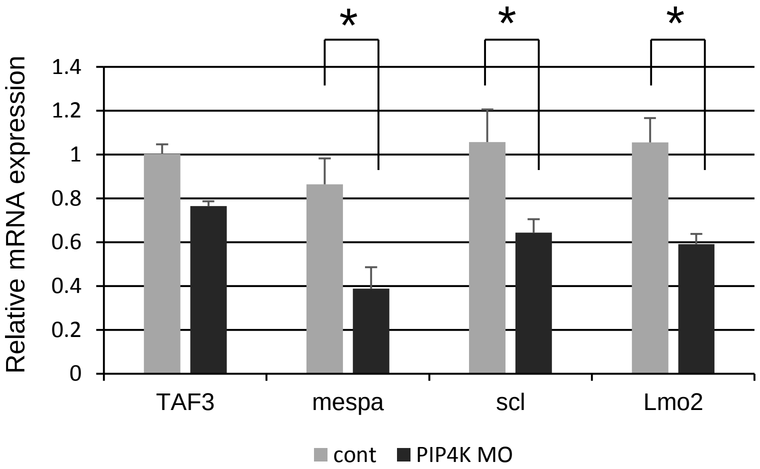


# figure S7 I

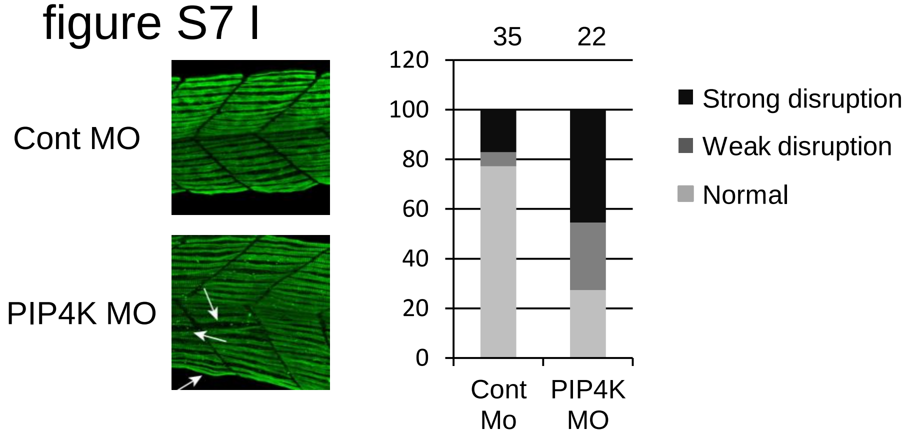

# Table S1 Characterisation of the interaction of PHD fingers with phosphoinositides and modified histone tails

| Column1 | gene name | Function | Domain | expression | interaction with lipid blot | lipid SPR | Preferential Histone tail SPR |
|---|---|---|---|---|---|---|---|
| 1 | ARHGEF28 | Rho guanine nucleotide exchange factor (GEF) 28 | phorbal/dag | | x | 2 | H3 unmodified |
| 2 | NSD1 | Histone methyltransferase. Preferentially methylates 'Lys-36' of histone H3 and 'Lys-20' of histone H4 | PHD4 | | | 1 | H3 unmodified |
| 3 | TRIM28 | transcriptional repressor | PHD | | | 0 | 0 |
| 4 | DPF2 | transcriptional regulation in the BRG1 complex | PHD1 and PH2 | | | 0 | 0 |
| 5 | KMT2A | Histone methyltransferase | PHD1,2 3 | Low | | 0 | H4K4me3 |
| 6 | KMT2A | Histone methyltransferase | PHD4 | Low | | 0 | 0 |
| 7 | PHF1 | component of a histone H3 lysine-27 (H3K27)-specific methyl- transferase complex | PHD1,2 | | | 0 | 0 |
| 8 | KDM4B | Histone demethylase that specifically demethylates 'Lys-9' and 'Lys-36' residues of histone H3 | PHD1,2 | | | 0 | 0 |
| 9 | BAZ2A | Essential component of the NoRC (nucleolar remodeling complex) complex | PHD | | | 1 | H3 unmodified |
| 10 | PHF20 | component of the MOF histone acetyltransferase protein complex | PHD | | | 0 | 0 |
| 11 | KDM5A | Histone demethylase that specifically demethylates 'Lys-4' of histone H3 | PHD1 | | | 0 | 0 |
| 12 | INTS12 | subunit of the Integrator complex, which associates with the C-terminal domain of RNA polymerase II | PHD | | | 1 | H3 unmodified |
| 13 | KTM2C | myeloid/lymphoid or mixed-lineage leukemia (MLL) family. Histone methyltransferase. Methylates 'Lys-4' of histone H3 | PHD4,5,6 | Low | | 0 | 0 |
| 14 | TAF3 | Component of the basal transcription complex TFIID | PHD | | x | 2 | H3K4me3 |
| 15 | KDM4B | Histone demethylase that specifically demethylates 'Lys-9' of histone H3 | PHD2 | Low | | 0 | 0 |
| 16 | PHF10 | component of the neural progenitors-specific chromatin remodeling complex (npBAF complex) | PHD1,2 | | x | 2 | H3 unmodified |
| 17 | CXXC1 | interacts specificity with unmethylated CpG motifs and is a component of the SET1A and B methyltransferase complex | PHD | | x | 1 | equal H3 and H3K4me1,2,3 inhibited by K9 di and trimethylation |
| 18 | PHF19 | Polycomb group (PcG) that specifically binds histone H3 trimethylated at 'Lys-36' (H3K36me3) | PHD1,2 | Low | x | 0 | 0 |
| 19 | PYGO1 | involved inWNT signalling | PHD | | | 0 | 0 |
| 20 | PHF14 | unknown | PHD2 | | x | 2 | H3 unmodified |
| 21 | PHF6 | mutated in Borjeson-Forssman-Lehmann syndrome (BFLS) and in AML and All | PHD1 | | x | 2 | H3 unmodified |
| 22 | PHF6 | mutated in Borjeson-Forssman-Lehmann syndrome (BFLS) and in AML and All | PHD2 | Low | x | 0 | 0 |
| 23 | ING4 | Component of the HBO1 complex which has a histone H4-specific acetyltransferase activity | PHD | | | 1 | H3 unmodified |
| 24 | TCF20 | Transcriptional activator stimulates the activity of various transcriptional activators such as JUN, SP1, PAX6 and ETS1 | PHD (ATYPICAL) | Low | x | 0 | 0 |
| 25 | ING3 | a tumor suppressor protein that can interact with TP53 and is a component of the NuA4 histone acetyltransferase (HAT) complex | PHD | | | 1 | H3K4me3 |
| 26 | KAT6B | Histone acetyltransferase component of the MOZ/MORF protein complex | PHD1,2 | | | 2 | H3 unmodified |
| 27 | DIDO1 | Putative transcription factor, weakly pro-apoptotic when overexpressed | PHD | | | 0 | H3K4me3 |
| 28 | UHRF1 | member of a subfamily of RING-finger type E3 ubiquitin ligases, binds hemimethylated DNA regulates transcription possibly by impacting DNA methylation and histone modifications. | PHD | | | 1 | H3 unmodified inhibited by k4 more than k9 methylation |
| 29 | BAZ1B | Atypical tyrosine-protein kinase phosphorylating 'Tyr-142' of histone H2AX and plays a central role in chromatin remodeling | PHD | | | 1 | 0 |
| 30 | KDM5C | Histone demethylase that specifically demethylates 'Lys-4' of histone H3 | PHD1 | | | 0 | 0 |
| 31 | MTF2 | Polycomb group (PcG) that specifically binds histone H3 trimethylated at 'Lys-36' (H3K36me3) | PHD1,2 | | | 0 | H3 unmodified |
| 32 | DPF1 | Belongs to the neuron-specific chromatin remodeling complex (nBAF complex) | PHD1,2 | | | 1 | H3 unmodified |

**Supplemental Figure legends.**

**Figure S1 related to Figure 1**

A. shX or sh-PIP4K2B C2C12 cells were differentiated for the number of hours indicated. The cells were fixed and stained with propidium iodide and analysed by flow cytometry.
B. shX or sh-PIP4K2B C2C12 cells were differentiated for the times indicated after which nuclei were isolated and analysed by immunoblotting with the indicated antibodies. The first lane shows the presence of the proteins in a total cell lysate.
C. shX or sh-PIP4K2B C2C12 cells were differentiated for two days after which nuclei were isolated and the mass of lipids was determined by mass spectrometry. The data are normalised to an internal standard and represent the mean of triplicates +SD (arbitrary units).
D. shX or sh-PIP4K2B C2C12 cells were differentiated for the times shown and gene expression levels were determined by QRT-PCR as indicated. The data represent fold changes compared to the 0 hour shX sample and represent the mean of triplicates +SD.
E. shX or sh-PIP4K2B C2C12 cells were differentiated for the times shown and gene expression levels were determined by QRT-PCR as indicated. The graph on the right shows the expression of PIP4K2B in the cell lines at the times indicated after differentiation. The data represent fold changes compared to the shX sample and represent the mean of triplicates +SD.
F. C2C12 were depleted of PIP4K2B using three different targeting constructs and representative images of myotube formation are shown. The knockdown efficiency is shown in Figure S1E.
G. shX or sh-PIP4K2B C2C12 cells were transduced with control vector (PB) or with a rescue vector encoding a fusion protein of PIP4K2B /2A. The cells were differentiated for 48h before expression of MYH and MYL was quantitated by QRT-PCR. The data represent fold changes compared to the shX sample and represent the mean of triplicates +SD.
H. shX or sh-PIP4K2B C2C12 cells were differentiated for six days after which myotubes and reserve cell populations were isolated by limited trypsinisation and replating. RNA was isolated and the expression of the indicated genes was determined by QRT-PCR. The data represent fold changes compared to the proliferating shX sample and represent the mean of triplicates +SD.

**Figure S2 related to Figure 2**

GST fusion proteins with characterised lipid interaction domains were purified and used to determine their interactions with PI presented on a micellar SPR chip surface. GST-PLC$\delta$1 PH domain interacted strongly with PI(4,5)$P_2$ and the 2XFYVE domain interacted strongly with PI3P as expected. The 2XPHD finger of ING2 showed interaction in this assay with PI5P, PI3P and PI(4,5)$P_2$.

**Figure S3 related to Figure 3**

A. The cysteine zinc finger mutant of the PHD finger of TAF3 was analysed for its interaction with PI. Wild type TAF3 PHD finger interacted strongly with PI (left panel) while the zinc finger mutant interaction was severely compromised (right panel). The zinc finger mutant

was also compromised in its interaction with H3K4me3 (data not shown). Residues in red indicate cysteine or histidine that were converted to serine residues.

B. Cartoon depicting the structure of TAF3 and the sequence of the extended PHD finger including the PBR region (highlighted). Bold residues indicate amino acids that constitute the canonical zinc finger interaction residues.
C. Sequences depicting the deletion mutants and mutants within the PBR used to determine exactly how TAF3 PHD finger interacts with PI. The gels show purified protein of the various mutants.
D. Upper panel shows the interaction of the deletion mutants with PI while the lower panel shows a subset of the PBR point mutants and their interaction with PI.
E. The interaction of various mutants with either PI or with histone peptides is summarised in the table. Increasing number of ticks refers to increased strength of interaction, while an X indicates no interaction.

**Figure S4 related to Figure 4**

A. Control cells (A) or cells with TAF3 knocked down (B) were differentiated for the days indicated after which RNA was extracted and the expressions of the indicated genes (above) were determined using QRT-PCR. The data represent fold changes compared to the 0 shX (A) sample and represent the mean of triplicates +SD.
B. Wildtype or sh-TAF3 C2C12 cells were rescued with wildtype (WT-TAF3) or mutants which only maintain H3K4me3 (KK-TAF3) or PI interaction (DW-TAF3), respectively. Cells were differentiated for 5 days and analysed by widefield microscopy. Arrows denote easily observable myotubes.

**Figure S5 related to Figure 5**

A. sh-TAF3 C2C12 cells were rescued with either empty vector,WT- or KK-TAF3 and then transduced with a control sh-RNA (shX) or with an sh-RNA targeting PIP4K2B (sh-PIP4K2B). Cells were then differentiated for 3 days before TAF3 or PIP4K2B expression (as indicated) was assessed by QRT-PCR. The data represent fold changes compared to the EV-shX (EV 1) sample and represent the mean of triplicates +SD.
B. sh-TAF3 C2C12 cells were rescued with empty vector (EV) or with WT- or KK-Ha-tagged TAF3. Cell lysates were immunoprecipitated using an antibody against TAF3 and immunoblotted using an antibody against HA.
C. sh-TAF3 C2C12 cells were rescued with either empty vector (EV), WT- or KK-TAF3 and then transduced with a control shX vector (1) or with a sh-RNA targeting PIP4K2B (2). Cells were differentiated for the days indicated before gene expression levels were assessed by QRT-PCR as indicated. The data represent fold changes compared to the 0 day EV-shX sample and represent the mean of triplicates +SD.
D. sh-TAF3 C2C12 cells were rescued with either WT (wt) or KK-TAF3 (kk) and then transduced with a control RNAi vector (shX) or one targeting PIP4K2B (sh-PIP4K2B ). Cells were differentiated for the number of days indicated, and expression levels of MYOD or PIP4K2B were analysed by QRT-PCR. The data represent fold changes compared to the 0 day shX sample and represent the mean of triplicates +SD.

**Figure S6 related to Figure 6**

A. Principal component analysis of the microarray gene expression data using the top 500 most variable genes. Each point represents a single array from sh-TAF3 C2C12 cells rescued with WT-TAF3 (green), WT-TAF3 and depleted of PIP4K2B (red) or KK-TAF3 and depleted of PIP4K2B (blue) (Figure 6A). The analysis shows a high degree of coherence between biological replicates and clearly shows separation of cells based on their genotype. For example, PC3 describes variation dependent on the expression of PIP4K2B but not dependent on the PI interaction site of TAF3. PC4 defines variability that depends on both expression of PIP4K2B and the interaction of TAF3 with PI.
B. Gene set enrichment analysis (GSEA) was used to identify gene expression programmes that are differentially regulated in two different conditions. Enrichment of gene sets is observed as a polarised presence of members of the gene set in a ranked list of expression differences. A gene set containing genes upregulated after myogenic differentiation of C2C12 cells (top panel) and genes induced by MyoD overexpression (bottom panel) were highly enriched in sh-PIP4K2B compared to shX C2C12 cells (left panel) and in WT- compared to KK-TAF3 C2C12 cells (right panel). This data illustrates that knockdown of PIP4K2B compared to control or expression of WT- compared to KK-TAF3 enhance the myogenic gene expression programme during differentiation.
C. Comparative expression analysis identified genes that were regulated by PIP4K2B knockdown or by the expression of KK- compared to WT-TAF3 in sh-TAF3 C2C12 rescue cells after differentiation for two days. The expression levels of 550 genes changed upon knockdown of PIP4K2B ($p<0.05$ and more than 1.4 fold difference), of which 331 were up regulated. 202 genes were differentially expressed in WT- compared to KK-TAF3 sh-TAF3 C2C12 rescue cells (1.4 fold cut off and $p<0.05$). A highly significant number of 63 genes formed the overlap between PIP4K2B- and KK-regulated gene sets (representation factor 11.9 and $p<3.79e^{-50}$). A representation factor above 1 suggests more than the expected overlap. The probability factor is based on exact hypergeometric probability with normal distribution http://nemates.org/MA/progs/representation.stats.html).
D. Comparative expression analysis identified genes that were regulated by PIP4K2B knockdown or by the expression of KK- compared to WT-TAF3 in sh-TAF3 C2C12 rescue cells after etoposide treatment. 236 genes were differentially expressed upon PIP4K2B knockdown, while 127 genes were differently expressed in WT- compared to KK-TAF3 sh-TAF3 C2C12 rescue cells, with a highly significant overlap of 30 genes between the two gene sets (representation factor 21, $p<2.89e^{-31}$).

**Figure S7 related to Figure 7**

A. HEK293 cells were transfected with TAF3 PHD finger constructs indicated (right) and lysates were used for affinity chromatography with beads coupled to unmodified histone H3 peptide (H3) or modified by trimethylation at lysine 4 (H3K4me3) or at lysine 9 (H3K4me3). Bound proteins were separated by SDS-PAGE and assessed by immunoblotting using an anti-HA antibody. The right panel depicts the input of the various TAF3 proteins.
B. HEK293 cells were transfected as indicated (bottom) and then immunoprecipitated with the antibodies indicated on the left and bound proteins were assessed using SDS-PAGE and immunoblotting with the antibodies indicated on the right. The lower panel shows the expression of the various proteins in the input lysates.

C. HEK293 cells were transfected as indicated (bottom) and immunoprecipitated with the antibodies shown on the left. Bound proteins were assessed by SDS-PAGE and immunoblotting with the indicated antibodies (right).
D. Increasing concentrations of WT-TAF3 PHD finger was assessed for its interaction with fluorescent H3K4me3 peptide in the absence (red line) and presence of PI5P (blue line) and PI(4,5)$P_2$ (green line). The data demonstrate that both PI5P and PI(4,5)$P_2$ decrease the interaction between TAF3-PHD finger and H3K4me3.
E. TAF3 Chip analysis at the promoter of the MYOG gene of shX or sh-PIP4K2B C2C12 cells before and after myoblast differentiation for 48h or 72h. The data is represented as fold enrichment over the shX control and are mean+SEM (n=2).
F. An alignment showing the strong evolutionary conservation of the PBR of the TAF3 PHD finger from various organisms.
G. Zebrafish embryos were injected with either a control morpholino (MO) or a PIP4K targeting morpholino (PIP4K MO) with or without RNA encoding the wild type human PIP4K2A (WT-PIP4K) or the kinase inactive enzyme (KD-PIP4K) and were collected 48h post fertilisation. PIP4K MO induces a developmental phenotype observed in approximately 70% of fish. The rescue was observed in 80% of injected fish with WT-PIP4K RNA but only in approximately 10% injected with the mutant kinase inactive PIP4K RNA.
H. Control zebrafish embryos (cont) or embryos injected with 3.5ng PIP4K MO were collected 24h post fertilisation and assessed for the expression of the TAF3 dependent transcription factor *mespa* and for direct Mespa downstream gene targets *scl* and *lmo2*. The data represent fold changes compared to the control samples and represent the mean +SD of triplicates. The data were normalised to the housekeeping gene GAPDH.
I. Zebrafish embryos were injected with a control- or PIP4K MO. Embryos were collected 24h post fertilisation and stained using F59 (MYHC). Representative images of the disruption of the myosin filament architecture by the indicated injections are shown. The severity of the phenotypes were categorised into strong and weak and presented graphically. The number of injected embryos is indicated above the graph.

**Table S1 related to figure 2.**

Table S1 summarises the interaction of the various PHD fingers with PI as assessed by either lipid dot blot or SPR analysis as well as their interaction with unmodified and methylated histone h3 tails. Column 1 numbers corresponds to numbers under the gel picture in Figure 2A. Of the 32 proteins tested by lipid dot blots, nine interacted strongly with one or more PI and in general interacted with PI3P, PI4P, PI5P and PI(3,5)$P_2$ (Figure 2B). SPR confirmed the interaction of six of the nine proteins identified using lipid dot blots and identified an additional 17 PHD fingers as PI interactors. None of the PHD fingers interacted with PI(4,5)$P_2$ when assessed by lipid dot blots but did when assessed using SPR. The expression of some proteins was very low (Low) indicating that they might be unfolded. Interaction assessed by SPR was characterised as strong (2) medium (1) or weak/non-interacting (0). The interaction of PHD fingers with histone tails was referenced to interaction with unmodified H3 tail peptide. H3 unmodified indicates that the PHD finger interacted more strongly with the unmodified H3 tail compared to methylated tails. H3K4me3 indicates preferential interaction with H3K4me3. 0 indicates that very little or no interaction was observed.

Supplemental experimental methods

Quantitative RT-PCR and Microarray analysis. QRT-PCR was carried out using specific primers (available on request) either using internal probes or using SYBR-Green. In the latter case specificity was determined using melting curves. Changes in expression were determined by the $\delta\delta$CT method with samples normalised to the level of GAPDH. Data are presented as the mean +SD of triplicates and all experiments were carried out at least twice. Global gene expression changes were analysed by microarray hybridisation analysis using an Affymetrix 2.0 mouse chips (CRUK Manchester Institute array facility). Data was normalised and gene expression differences were determined using a 1.4 fold threshold and a $p<0.05$.

sh-RNA mediated knockdown: sh-RNA sequences targeting TAF3 were cloned into pRetroSuper and retroviral particles were generated in either Phoenix-ECO or Plat-E cells and used to infect C2C12 cells in the presence of polybrene (5μg/ml) overnight. Cells were washed free of virus and selected with puromycin (5μg/ml). For rescue experiments full length TAF3 (WT, KK or DW) or PIP4K2B were cloned into pBABE and used to generate retroviral particles in either phoenix-ECO or Plat-E cells. C2C12 cells were infected as above and selected accordingly.

Lentiviral sh-RNA: sh-RNA targeting PIP4K2B or a control sequence (shX) cloned in the pLKO 1 or 2 vector were purchased from SIGMA (St Louis, USA). Viral particles were generated in HEK293FT cells using pLKO-based vectors and plasmids encoding GAG-Pol and VSVG (4:2:1, respectively). C2C12 cells were transduced in the presence of polybrene (5μg/ml) and selected using puromycin (5μg/ml).

Immunofluorescence. Staining was carried out using the appropriate antibodies diluted 1:100 after fixation with 4% formaldehyde. Cells were permeabilised using 0.1% TX-100-PBS and non specific sites were blocked using 3% BSA-PBS. Primary antibody incubation was carried out in the 1% BSA-PBS for 1 hour (h) after which cells were washed and incubated in the appropriate secondary antibody diluted 1:200 in 1% BSA-PBS for 1h. After washing, cells were stained with DAPI and mounted using antifade reagent. Immunofluorescence was visualised using a spinning disk confocal microscope (Olympus IX81).

Luciferase assays. C2C12 cells were transfected with luciferase constructs driven by the indicated promoters together with renilla luciferase driven by the thymidine kinase promoter as a control. Cells were differentiated for two days after which the cells were lysed in passive lysis buffer (Promega) and luciferase activities were analysed using the Stop and Glo reagents (Promega). Firefly luciferase counts (promoter specific) were normalised for the renilla counts (control plasmid).

Cloning and purification of GST fusion proteins. PHD fingers were identified using the SMART module and fragments were amplified with Phusion polymerase using specific primers (available on request). Fragments were T-tailed with Taq polymerase and directly cloned into the Sma1 site (previously A-tailed). Successful cloning was assessed by scoring single colonies for protein production and subsequently each clone was verified by sequencing (PHD finger sequences available on request).

Single bacterial colonies were grown in 10ml LB overnight, diluted 20 fold in the morning and grown for another 3h at 37°C. Fusion proteins were induced by the addition of 50μM IPTG and 10μM $ZnSO_4$ and grown overnight or for 4h at 25°C. Bacterial pellets were washed once in PBS before lysis by sonication in 1% TX-100-PBS containing complete EDTA free Roche protease inhibitors. GST proteins were isolated by incubation with glutathione beads (GE Healthcare) for 2h, washed and eluted using 50mM TRIS pH 8.0, 300mM NaCl, 10mM glutathione, 10mM DTT, 10μM $ZnSO_4$ . Protein concentration was assessed using BioRad reagent.

Lipid Dot blots. 50pmoles of PI lipid dissolved in chloroform (2µl) were spotted onto nitrocellulose membranes, which were blocked in 3% BSA-TBS before incubation with GST-proteins (0.2µg/ml diluted in 3% BSA-TBS) for 2h. After washing, blots were incubated with an anti-GST-antibody and interaction was visualised using an anti-mouse antibody conjugated to HRP and enhanced chemiluminescence (Super Signal Pierce).

SPR analysis.
Lipid interaction: The sensor surface was conditioned by vertical injections of 0.5% SDS, followed by 50 mM NaOH and 100 mM HCl at 30 µl/min for 60s after which undecylamine was coupled using NHS/EDC to prepare a hydrophobic capture SPR sensor chip. PI (1nmole) or phosphatidylcholine (1nmole) were sonicated into 100µl of 10mM Tris pH 7.4 and diluted in 50mM acetate pH 5.5. Approximately 500RU units were captured on the SPR chip. If necessary, lipids were reloaded to achieve equal loading levels. Recombinant GST-tagged proteins (analyte) were diluted in the respective SPR buffer to the desired concentration (usually 100nM to 1000nM). Analyte injections were generally performed at a flow rate of 50 µl/min (sometimes 100 µl/min) and 25°C sensor chip temperature. Association of analyte molecules to the lipid-ligands was measured for 120s to 240s and dissociation of analyte molecules was measured for 240s to 600s. Following each analyte injection, the sensor chip surface was regenerated with two injections 50mM NaOH at 100 µl/min for 18s. Analysis was carried out with at least two separate protein purifications, each being analysed by multiple injections.
Histone tail interaction: An NLC (BioRad) sensor surface was conditioned by vertical injections of 1M NaCl, 50mM NaOH, 1M NaCl and 50mM NaOH at 30 µl/min for 60s. Six different types of biotinylated histone peptides were captured onto the NeutrAvidin surface at 30 µl/min for 90s to 200s at 25°C. The sensor surface was post-conditioned with two vertical, followed by two horizontal injections of 10mM HCl at 100 µl/min for 18s. Analyte injection were carried out as described for lipid interaction.

Fluorescence polarization: Experiments were performed in FP buffer (PBS; 10µM $ZnSO_4$) using a HYDEX Chameleon plate reader at 4°C as previously described (Fischle et al., 2008). The association of N-terminally fluorescein-labelled peptides H3 (aa 1-15) unmodified and K4me3 with purified recombinant WT, KK and DW-TAF3 GST-fusion protein was measured in the absence and presence of 5-fold molar excess of PI5P Di-C4. Fluorescence anisotropy (FA) was calculated from measured intensities in the parallel and orthogonal plane. Raw anisotropy data were analyzed implying a one site binding model using the equation $A = A_f + (A_b – A_f)([R]/(K_d – [R]))$ with A, Anisotropy; $A_b$, anisotropy of bound state; $A_f$, anisotropy of unbound state; $K_d$, dissociation constant; [R], protein concentration. Curves were fit by least square fitting. Anisotropy readings were converted to fraction bound using the equation $F_b = (A - A_b)/(A_b - A_f)$; with $F_b$, fraction bound. Readings from multiple independent (minimum: 3) measurements were averaged after normalisation and blotted. Data was analysed using Prism graph pad 6. Analyses are derived from two separate protein purifications.

CHIP analysis

TAF3 and RNAPII ChIP assays were performed either as described (Lauberth et al., 2007) or using the Diagenode high cell ChIP kit. Cells were reversibly crosslinked in 1% (final) formaldehyde (Sigma) for 10min at room temperature and quenched by adding 125 mM (final) glycine (Sigma). Isolated chromatin was fragmented to an average size of 200-600bp with a biorupter (Diagenode). Precleared chromatin was immunoprecipitated overnight at 4°C and immunocomplexes were

collected with Protein A or G agarose coupled with salmon sperm DNA for 1.5h at 4°C. The immunocomplexes were eluted, crosslinks were reversed at 65 ºC for 4hr, and DNA was purified using QIA-quick spin columns according to manufacturer's instructions. CHiP DNA was analysed using qPCR.

Reference List

Fischle,W., Franz,H., Jacobs,S.A., Allis,C.D., and Khorasanizadeh,S. (2008). Specificity of the chromodomain Y chromosome family of chromodomains for lysine-methylated ARK(S/T) motifs. J. Biol. Chem. *283*, 19626-19635.

Lauberth,S.M., Bilyeu,A.C., Firulli,B.A., Kroll,K.L., and Rauchman,M. (2007). A phosphomimetic mutation in the Sall1 repression motif disrupts recruitment of the nucleosome remodeling and deacetylase complex and repression of Gbx2. J. Biol. Chem. *282*, 34858-34868.
